# Robust Motion Regression of Resting-State Data Using a Convolutional Neural Network Model

**DOI:** 10.3389/fnins.2019.00169

**Published:** 2019-02-28

**Authors:** Zhengshi Yang, Xiaowei Zhuang, Karthik Sreenivasan, Virendra Mishra, Dietmar Cordes, the Alzheimer’s Disease Neuroimaging Initiative

**Affiliations:** ^1^Cleveland Clinic Lou Ruvo Center for Brain Health, Las Vegas, NV, United States; ^2^Department of Psychology and Neuroscience, University of Colorado, Boulder, Boulder, CO, United States

**Keywords:** fMRI, denoising, convolutional neural network, motion artifact, nuisance regression

## Abstract

Resting-state functional magnetic resonance imaging (rs-fMRI) based on the blood-oxygen-level-dependent (BOLD) signal has been widely used in healthy individuals and patients to investigate brain functions when the subjects are in a resting or task-negative state. Head motion considerably confounds the interpretation of rs-fMRI data. Nuisance regression is commonly used to reduce motion-related artifacts with six motion parameters estimated from rigid-body realignment as regressors. To further compensate for the effect of head movement, the first-order temporal derivatives of motion parameters and squared motion parameters were proposed previously as possible motion regressors. However, these additional regressors may not be sufficient to model the impact of head motion because of the complexity of motion artifacts. In addition, while using more motion-related regressors could explain more variance in the data, the neural signal may also be removed with increasing number of motion regressors. To better model how in-scanner motion affects rs-fMRI data, a robust and automated convolutional neural network (CNN) model is developed in this study to obtain optimal motion regressors. The CNN network consists of two temporal convolutional layers and the output from the network are the derived motion regressors used in the following nuisance regression. The temporal convolutional layer in the network can non-parametrically model the prolonged effect of head motion. The set of regressors derived from the neural network is compared with the same number of regressors used in a traditional nuisance regression approach. It is demonstrated that the CNN-derived regressors can more effectively reduce motion-related artifacts.

## Introduction

Resting-state functional magnetic resonance imaging (rs-fMRI) based on the blood-oxygen-level-dependent (BOLD) signal has been widely used to investigate brain functions when the subject is in a resting or task-negative state. The BOLD signal, however, is contaminated by multiple noise sources, including head motion, cardiac and respiratory motion, thermal motion inherent to electrical circuits, instrumental drift, and changes in blood pressure and cerebral autoregulation mechanisms, which may severely corrupt BOLD fMRI time series (Murphy et al., 2013). A few recent studies have demonstrated that head motion can significantly confound the analysis of rs-fMRI data (Power et al., 2012; Satterthwaite et al., 2012; Van Dijk et al., 2012). These studies came to a consensus that motion overall tends to increase short-range correlations to nearby voxels, causing functional connectivity (FC) to vary with distance between regions. Even small amounts of motion can have considerable influence on connectivity measurement (Yan et al., 2013).

The origin of motion-related signal changes can be explained in terms of three interrelated aspects (Caballero-Gaudes and Reynolds, 2017). First, any alteration in tissue composition due to head motion can cause a change in net magnetization and thus proportionally change the amplitude of the signal in a voxel. Second, the number of excited spins depends on the position of a voxel at the current time point and previous time points. Head movement alters the timing between successive spin excitations in the voxel, potentially generating spin history artifacts and thus impacting the signal even beyond the instantaneous time points. Third, the inhomogeneous magnetic field induced by head movement changes the spatial distribution of the local magnetic susceptibility gradients and exacerbates distortions and signal dropouts in regions sensitive to these effects (Jiang et al., 1995).

In the last decade, nuisance regression has been a popular preprocessing strategy to remove motion artifact in rs-fMRI data. A set of motion regressors, referred as nuisance regressors, is first specified to characterize motion-related fluctuations in the data. The denoised data is then obtained by regressing out the contributions from the motion regressors from the original data.

The selection of nuisance regressors is a critical factor influencing the performance of nuisance regression. Inappropriate regressors may have negligible effect or even be detrimental to the analysis. For example, inclusion of the global signal (i.e., the average fMRI signal across the whole brain) as a nuisance regressor has been heavily debated in the past. Multiple studies have shown that global signal regression (GSR) may introduce a negative bias in the estimated BOLD response (Macey et al., 2004; Saad et al., 2012), artificially generate anti-correlation between brain regions (Murphy et al., 2009), and strengthen the relationship between motion-connectivity correlation and regional Euclidian distance (Satterthwaite et al., 2013). The most common motion regressors are simply the six head motion parameters (R = [X Y Z pitch yaw roll]) estimated from the fMRI rigid-body realignment pre-processing step. To further reduce motion-induced spin history artifacts, 12, 24, or even 36 motion-related regressors are used in recent studies, which incorporate original motion parameters, their first-order derivatives, their squared functions, motion parameters with one or two temporal shifts or average tissue-based [gray matter (GM), white matter (WM), cerebrospinal fluid (CSF)] regressors (Friston et al., 1996; Power et al., 2012; Van Dijk et al., 2012; Satterthwaite et al., 2013; Yan et al., 2013). An alternative strategy for carrying out motion correction is to scrub contaminated volumes from fMRI data prior to data analysis (Lemieux et al., 2007; Power et al., 2011, 2012). Typically, time points are first identified as motion-induced artifacts by thresholding certain motion measurements, e.g., framewise displacement, then spike regressors are created with a single non-zero value at each identified time point as well as its neighboring time points, and finally these spikes are regressed out to generate spike-free data. This scrubbing strategy can be treated as excluding contaminated time points from subsequent analysis. The combination of scrubbing and motion regression was shown to have the greatest reduction in motion-related artifacts (Satterthwaite et al., 2013). However, there is a tradeoff between the data quality and remaining time points. Similar to general nuisance regression, including more motion regressors can be detrimental to the following analysis since it is unclear whether significant amount of the neuronal-related BOLD signal is also removed. In addition, scrubbing has the potential limitation of removing a large proportion of time series from a single subject, leading to significant variation in the number of remaining time points from one subject to another (Yan et al., 2013).

While there are other approaches to reduce motion-related artifacts such as slice-wise motion correction (Beall and Lowe, 2014), acquiring data with multi-echo EPI sequences (Kundu et al., 2012) and ICA-based motion correction approaches (Griffanti et al., 2014; Pruim et al., 2015), this study focuses on using the head motion parameters estimated from rigid-body realignment to derive optimal motion-related fluctuations in rs-fMRI data. The relationship between head motion and the resulting change in the MR signal remains unclear, the realignment parameters and their temporal derivatives or squared functions may not be sufficient to model the non-linear MR signal change in the data. We have developed a robust and automated convolutional neural network (CNN) model to derive improved motion regressors. In the recent past, CNN networks achieved classification accuracy record with ImageNet data (Krizhevsky et al., 2012) and have been successfully applied in different fields such as object recognition and sentence classification (Kim, 2014; Liang and Hu, 2015). In our proposed CNN model, the motion parameters estimated from rigid-body realignment are the input to the network. Considering that voxels within white matter and CSF share similar motion-related artifacts as the voxels within GM but do not have neural contributions, time series from WM and CSF but not GM are used for optimizing model parameters to avoid reducing neural activations.

The CNN network consists of two temporal convolutional layers and the output data from the network are the optimized motion regressors used in a subsequent motion regression. The temporal convolutional layer in the network is particularly useful for non-parametrically modeling the prolonged effect of head motion (Power et al., 2014). The regressors derived from the neural network are compared with the same number of regressors used in a traditional motion regression approach. A comprehensive comparison of these two methods of motion regression is presented using fMRI data from a publicly available database.

## Materials and Methods

### Subjects

The structural MRI and rs-fMRI data used in this study were downloaded from the publicly available ADNI database^1^. The ADNI was launched in 2003 as a public-private partnership, led by Principal Investigator Michael W. Weiner, MD. The primary goal of ADNI has been to test whether serial MRI, positron emission tomography, other biological markers, and clinical and neuropsychological assessment can be combined to measure the progression of mild cognitive impairment and early Alzheimer’s disease.

Only the subjects identified as normal controls by site investigators were used in this study. All subjects were scanned on a 3.0-Tesla Philips MRI scanner. All data were downloaded from the ADNI database before September 2016, and 76 subjects (age 74.1 ± 6.6 years, MMSE 28.9 ± 1.3, handedness 67 right/9 left, gender 33 male/43 female) were found satisfying the conditions described above. The subject ID, scanning parameters and demographical information can be found in Supplementary Table S1. The magnetization prepared rapid acquisition gradient echo (MP-RAGE) sequence was used to acquire T1-weighted structural images by the investigators of the ADNI consortium. The structural MRI scans were collected with a 24 cm field of view and a resolution of 256 × 256 × 170 to yield a voxel size of 1 mm × 1 mm × 1.2 mm. The rs-fMRI data were acquired using an echo-planar imaging sequence with parameters: 140 time points; TR/TE = 3000/30 ms; flip angle = 80 degrees; 48 slices; spatial resolution = 3.3 mm × 3.3 mm × 3.3 mm and imaging matrix = 64 × 64. Details of the ADNI MRI protocol can be found on the ADNI website^2^. If a subject had multiple MRI/fMRI scans satisfying the requirements specified above, the first available MRI/fMRI data set was used for analysis.

### General fMRI Preprocessing

Functional and structural MRI imaging data are processed using the SPM^3^ and ANTs^4^ toolbox. The first five volumes of rs-fMRI data are discarded to avoid data with unsaturated T1 signal. Before motion regression, the following fMRI preprocessing steps are applied: (i) slice timing correction; (ii) rigid-body head motion correction to the mean EPI image using 7th order B-Spline interpolation to estimate realignment parameters; (iii) co-registration to the skull-stripped structural image; (iv) standard space normalization to the MNI152 2 mm template; (v) spatial smoothing with 6 mm full width at half maximum; (vi) linear detrending. Motion regression is applied after these general fMRI preprocessing steps are completed. Temporal filtering is a preprocessing step commonly used after motion regression (Satterthwaite et al., 2013; Power et al., 2014). Since we aimed to develop an automated method modeling motion fluctuation and compare it with traditional motion regressors, temporal filtering is not used to give a direct comparison of motion-corrected fMRI data.

### Deep Neural Network for Denoising

**FIGURE 1:**
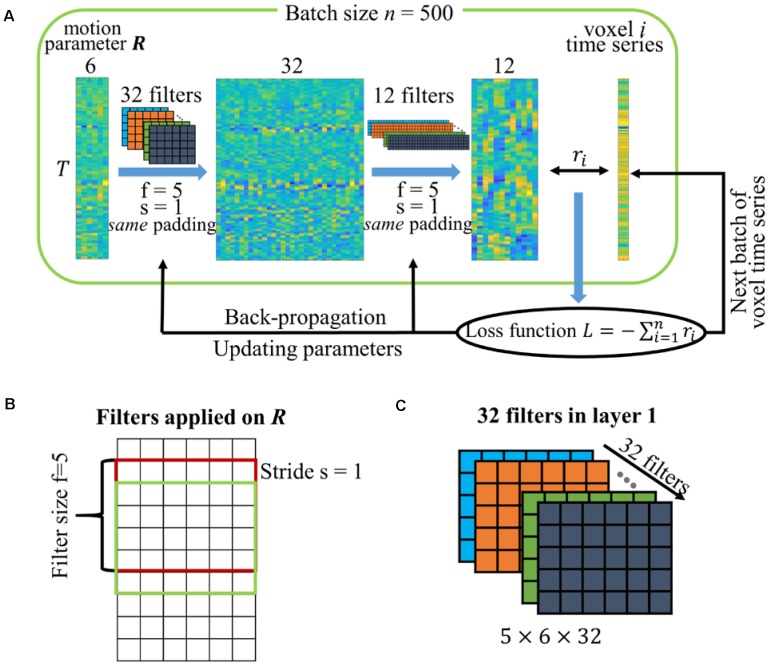
**(A)** Schematic diagram of the CNN network. The network has two temporal convolutional layers with 32 and 12 filters. The filters are specified with filter size *f* = 5, stride length *s* = 1 and the *same padding* so that the output length is the same as the original input. The motion parameters are replicated n times to match the number of voxels in the batch. The correlations between voxel time series and output regressors are used to calculate the loss function for model optimization. **(B)** Graphical explanation of filter size and stride. **(C)** Dimension of the 32 filters in the first convolutional layer.

The CNN denoising network is implemented using Keras^5^ with Theano^6^ as backend. The schematic diagram of the CNN network is shown in Figure 1A. The two sequential layers used in the CNN network are a 1-dimensional convolutional layer along the temporal direction. Previous studies showed that motion can have a prolonged and varying effect in the data (Patel et al., 2014; Power et al., 2014) and small amounts of movement could have substantial impact on the BOLD signal in fMRI data (Yan et al., 2013). The CNN network is proposed to learn the influence from the data without manual interference. Both layers have filter size *f* = 5, stride length *s* = 1 and *same* padding so that the output has the same length as the original input. The filter size is defined as the number of neighboring time points included when performing the convolution, and the stride length *s* = 1 means that the filter convolves the input volume by shifting one unit at a time. Figure 1B shows how the filters in the first convolutional layer are applied on the input with filter size and stride length defined and more detailed explanation about these hyperparameters (i.e., filter size, stride, and *same* padding) can be found on the Keras website. In these two convolutional layers, 32 temporal filters (filter dimension 5 × 6 × 32 as shown in Figure 1C with bias vector 32 × 1) are specified for the first one, and 12 temporal filters (filter dimension 5 × 32 × 12 with bias vector 12 × 1) are specified for the second one to match the number of traditional motion regressors used in this study, leading to 2,924 parameters in total in the neural network. We have also applied the network with different hyperparameters, including filter size and the number of temporal filters for the first layer. The setting described above achieved the least validation error and is selected in this study.

The realignment parameters ***R*** ∈ R^T×6^ are the only input data to our constructed CNN network, where *T* is the number of time points. The realignment parameters ***R*** are replicated to match the number of voxels within WM and CSF masks. Each replicate is linked with different time series within WM and CSF masks to make each pair unique. Naturally, thousands of WM and CSF time series paired with the duplicates of realignment parameters are the large number of samples required to optimize designed network, and each pair can be treated as a sample. With the assumption that WM and CSF voxels share similar motion-related artifacts as GM voxels but are not likely to have neural signals, voxels limited to non-GM (i.e., WM and CSF) are used to derive optimal motion regressors without erroneously modeling neural signals. Many (if not all) standard denoising techniques (Behzadi et al., 2007; Griffanti et al., 2014; Pruim et al., 2015) have used this assumption to reduce motion artifacts or physiological noise. While a few studies showed activation also in white matter (Gawryluk et al., 2014; Courtemanche et al., 2018), the question whether there is BOLD signal in white matter is debatable because of the lack of neurons in white matter.

These non-GM voxels are randomly assigned to a set of batches with batch size *n* = 500. In each batch, the input motion parameters ***R*** are replicated n times to match the number of voxels in the batch. These duplicate samples become unique and meaningful when they are linked to different voxel time series. In detail, the replicated motion parameters are forward-propagated through the convolutional layers and the output with dimension n × T × 12 is obtained for this batch. Naturally, each “sample” has the same output regressor with dimension $\tilde{R}$ ∈ R^T×12^. The correlation *r*_i_ between voxel time series and the 12 output regressors is calculated and the sum of correlation across all voxels in the batch with a minus sign is defined as the loss function to be minimized, namely $L=-\sum_{i=1}^{n}r_{i}$.

There are two choices to calculate the correlation between time series and the regressors $\tilde{R}$. The first one is by applying the general linear model (GLM) to fit the time series ***y***_i_ from voxel *i* to the regressors $\tilde{R}$ and then calculating the correlation between ***y***_i_ with the estimated time series ${\hat{y}}_{i}$ = GLM($\tilde{R}$, ***y***_i_), namely,

(1) $$\mathrm{choice}\;1:r_{i}=\mathrm{corr}(y_{i},\;{\hat{y}}_{i})\;\mathrm{and}\;{\hat{y}}_{i}=\mathrm{GLM}(\tilde{R},\;y_{i})=\tilde{R}\;{\tilde{R}}^{+}y_{i}.$$

The second choice is by calculating the maximal correlation between ***y***_i_ and each single regressor in $\tilde{R}$ with sign ignored, namely,

(2) $$\mathrm{choice}\;2:r_{i}=\max_{j}|\mathrm{corr}(y_{i},\;{\tilde{R}}_{j})|,\;\;j=1,\;\ldots ,\;12.$$

Considering that the pseudoinverse of output regressor matrix $\tilde{R}$, namely ${\tilde{R}}^{+}$, is required for choice 1 and needs to be updated for each batch, choice 2 is more computational efficient and is used to compute the loss function in this study. Once the loss function is obtained, its gradients are computed for updating the model parameters by back-propagation and the current batch of time series is replaced with another batch for the next iteration. Running through all batches once is called one epoch. The CNN network converges in less than 40 epochs for the fMRI data with 135 time points. The computational time for each subject is less than 2 min on a Tesla K40c GPU with 2,880 cores and approximately 10 min per subject with GPU disabled.

While all subjects share the same network architecture, the CNN network is optimized for each subject separately to achieve subject-specific model (the same architecture but different parameters). During the optimization, 90% of voxels are assigned to update model parameters and the remaining 10% of voxels are assigned to monitor whether the network suffers from over-fitting or under-fitting leading to high bias or variance, respectively. The initial parameters are randomly sampled from the *Xavier uniform initializer* (Glorot and Bengio, 2010). The parameters are updated with the *Adam* stochastic gradient-based optimization algorithm (Kingma and Ba, 2015), which adapts the parameter learning rates by taking advantage of both the average first moment (mean) and the average of the second moments of the gradients (uncentered variance). The *Adam* optimizer is parameterized with learning rate *η* = 0.01, learning rate decay *γ* = 0.05, exponential decay rate for the first moment estimates β_1_ = 0.9 and exponential decay rate for the second moments estimates β_2_ = 0.999. The neural network is tested with different activation functions including linear, sigmoid and rectified linear units (ReLU) (Nair and Hinton, 2010) to derive motion regressors. Linear and sigmoid activation functions have comparable performance, but ReLU sometimes leads to invalid loss function due to numerical instabilities. The result obtained with linear activation function is shown in the current study. The subject-specific optimal output regressors are applied on the same subject for reducing motion-related fluctuation.

### White Matter and Cerebrospinal Fluid Mask

The segmentation of the T1 image is carried out in the native space of each individual subject and the resultant tissue masks are normalized to the standard MNI152 space. The WM and CSF masks are eroded to reduce partial volume effects from neighboring GM voxels. Eroding the masks is crucial in our study because of the following two aspects. First, because non-GM time series are used in the CNN network to train the parameters in the model, the output regressors can account for some of the variance of BOLD signal if masks are not eroded. Second, the average time series of WM and CSF are used as nuisance regressors in our analysis and these two tissue-based regressors within un-eroded masks can also contain some BOLD signal. Inclusion of BOLD signal in nuisance regression has the potential of reduce the statistical power of fMRI data in the subsequent analysis. WM and CSF masks are eroded by the SPM *spm_erode.m* function. The CSF mask is eroded once as suggested in Power et al. (2014). To have enough WM voxels to train the CNN network and also minimize partial volume effects, the WM mask is eroded multiple times but contains at least 10,000 voxels. Both the non-GM time series used in the neural network and the average tissue-based regressors are extracted based on eroded masks.

### Motion Regressors

The CNN network designed above has 12 output regressors, referred as *cnn12* in the following. Unless explicitly specified, the input data to *cnn12* are the motion parameters ***R***. The *cnn12* regressors for all subjects can be found in the Supplementary Material. The motion parameters ***R*** and their temporal backward derivative ***R’***, referred to as *mot12*, are used in traditional motion modeling. The [***R R’***] motion regressors in *mot12* are equivalent to another set of 12 motion regressors [***R R*_t-1_**] used in other studies (Friston et al., 1996; Yan et al., 2013), where *t*-1 refers to the immediately preceding time point and the first row for regressors ***R_t-1_*** for *t* = 1 is traditionally filled with zeros. While previous studies employed varying number of regressors, including 6 regressors (***R***), 12 regressors ([***R***, **R**’]), 24 regressors ([***R*** ***R***^2^ ***R***_t_***_-_***_1_ ***R***_t_***_-_***_1_^2^]) and 36 regressors ([***R*** ***R***^2^ ***R***_t_***_-_***_1_ ***R***_t_***_-_***_1_^2^ ***R***_t_***_-_***_2_ ***R***_t_***_-_***_2_^2^]) (Friston et al., 1996; Power et al., 2012, 2014; Satterthwaite et al., 2013; Wilke, 2012; Yan et al., 2013), only *mot12* is compared in detail with *cnn12* in this study. Tissue-based signals are also used as nuisance regressors in part of our analysis and computed as the average signal across the voxels within either eroded WM or eroded CSF masks as described in the previous section. The inclusion of GSR has been heavily debated in the recent past (Murphy et al., 2009; Weissenbacher et al., 2009; Satterthwaite et al., 2013; Power et al., 2014, 2018), hence GSR is not used in this study. Unless explicitly specified, the functional atlas with 264 regions of interest (ROIs) (Power et al., 2011) is used to compute FC.

### Motion Measurements

Framewise displacement (FD) (Power et al., 2012), root-mean-square framewise displacement (rmsFD) (Satterthwaite et al., 2013), and DVARS, where D is referring to temporal derivative of time courses and VARS referring to root-mean-square of the variance over voxels (Smyser et al., 2010), are the measurements defined to provide a single estimated head motion parameter for each time point. We also use mean whole-brain standard deviation and modularity quality (Q) to provide a single measurement for each subject.

The motion measurements FD and rmsFD are derived from rigid-body realignment parameters, including three translational and 3 rotational parameters specified by ***R*** = [X Y Z yaw pitch roll]. The value of FD is defined as the sum of absolute derivatives of these six parameters with the three rotational parameters converted to distance by multiplying with a radius of 50 mm. The value of rmsFD is defined as the root mean square of relative displacement of two neighboring volumes. The subjects having mean FD ≥ 0.25 mm are assigned to the high-motion group (41 subjects, age 74.9 ± 7.2 years, MMSE 28.7 ± 1.6, handedness 36 right/5 left, gender 20 male/19 female) and the subjects having mean FD < 0.25 mm are assigned to the low-motion group (35 subjects, age 73.2 ± 5.8 years, MMSE 29.1 ± 0.9, handedness 31 right/4 left, gender 13 male/22 female). Unlike FD and rmsFD that are derived from estimated motion parameters, DVARS (Smyser et al., 2010) and mean whole-brain variance are computed based on fMRI data itself. DVARS is defined as the root mean square of the temporal change of the fMRI voxel-wise signal at each time point. Mean whole-brain variance for one subject is computed by first converting fMRI time series to percent signal change and then calculating the mean of the variance of all voxels across the entire brain. In this study we used modularity quality Q to evaluate whether BOLD signal is removed in addition to motion-related fluctuation. The *Q*-value is determined by applying community detection on each subject’s functional network using the Louvain heuristic (Blondel et al., 2008), which maximizes the *Q*-value as the criterion to partition the functional connectome into sub-networks. Subject motion has been shown to be negatively correlated to the *Q*-value in Satterthwaite et al. (2012), and the *Q*-value is expected to decline if the signal is removed (Ciric et al., 2017). An increased *Q*-value would indicate that the denoising method effectively reduces noise in the data without changing the signal.

## Results

**FIGURE 2:**
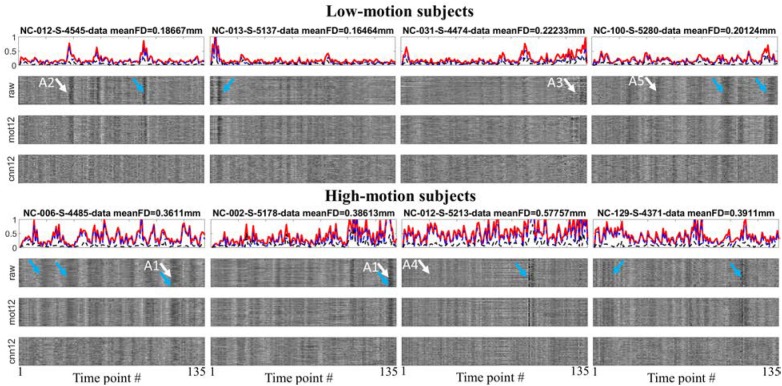
Time-dependent whole brain motion measurements and different preprocessed time series within the GM mask for 4 low-motion and 4 high-motion subjects. White arrows are used to point effects of motion on fMRI signal. Blue arrows are used to point out the performance difference between *cnn12* and *mot12*. The motion measurements include FD (red), sum of absolute translational parameters (blue) and sum of absolute rotational parameters (black).

To visualize the influence of motion regression, motion measurements and fMRI time series for 8 subjects are shown in Figure 2. The figures for all subjects can be found in the Supplementary Material. The FD (red), sum of absolute translational parameters (blue) and sum of absolute rotational parameters (black) are presented in the top panel for each subject. The normalized time series within GM mask processed with only general preprocessing steps (raw), traditional motion regression *mot12* and CNN-derived motion regression *cnn12* are plotted on the second, third, and fourth panel, respectively. Head movements are observed to have highly variable influences on fMRI signal in terms of three aspects: (1) motion can corrupt fMRI signal with varying duration (the width of dark band in the plot, e.g., arrows A1), (2) the direction of signal change could be mostly in the same direction (e.g., arrow A2) or be opposite at different voxels (e.g., arrow A3), (3) a large head movement may not have visually obvious effect (e.g., arrow A4) but a small head movement can produce marked effect (e.g., arrow A5). By visually inspecting these time series, *cnn12* has a better performance than *mot12* in reducing marked effects, particularly at the time points marked by blue arrows. A quantitative comparison is presented in the following.

**FIGURE 3:**
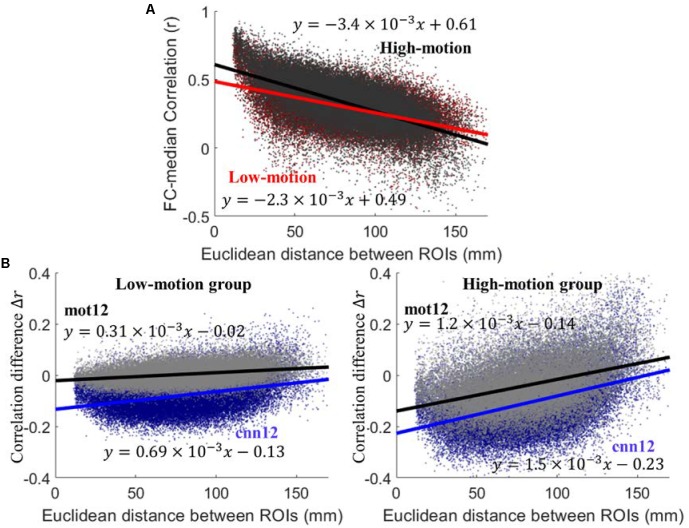
**(A)** Plots of functional connectivity versus Euclidean distance between ROIs using raw data. **(B)** Plots of functional connectivity versus Euclidean distance for low- and high-motion groups. Pearson correlation coefficient *r* is used to calculate functional connectivity and Δ*r* is defined as Δ*r* = *r*(after motion regression) - *r*(before motion regression).

Similar to Power et al. (2012) we have calculated FC difference before and after motion correction to evaluate the performance. Functional connectivity is computed as Pearson correlation between regional time series. For both high-motion and low-motion subjects, the scatter plot of between-region connectivity using raw fMRI data versus inter-node distance is shown in Figure 3A. The high-motion (black) and low-motion (red) group have shown negative linear relationship with Euclidean distance between ROIs with slope of -3.4 × 10^-3^ and -2.2 × 10^-3^, respectively. The dependency for the high-motion group is significantly stronger than the dependency for the low-motion group (*p* < 10^-4^). The plots of correlation difference Δ*r* versus Euclidean distance between ROIs are shown in Figure 3B, where Δ*r* < 0 indicates reduced correlation and Δ*r* > 0 indicates increased correlation after motion regression. The *cnn12*-processed data (blue) shows significantly (*p* < 10^-3^) stronger trend and lower intercept (larger magnitude) than *mot12*-processed data (black) for both high- and low-motion groups. Furthermore, the trend between Δ*r* and distance is significantly (*p* < 10^-4^) stronger and the intercept is also significantly (*p* < 10^-4^) lower in the high-motion group for both *cnn12* and *mot12* processed data.

**FIGURE 4:**
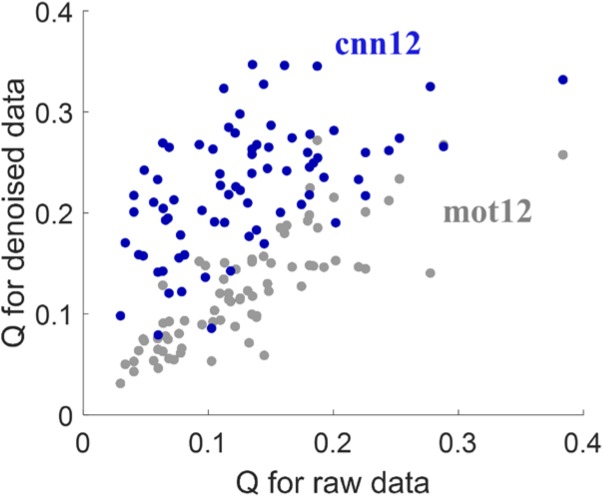
Modularity quantity Q measurement for different processed data. The *Q*-value is computed by applying community detection on each subject’s functional network using the Louvain heuristic, which maximizes the *Q*-value as the criterion to partition the network into sub-networks.

With the 264-ROI FC matrices, the modularity quality Q was computed for each subject. Figure 4 shows the scatter plot of *Q*-values for denoised data versus the *Q*-values for raw data. The proposed *cnn12* method (blue dots in Figure 4) significantly (paired *t*-test, *p* < 0.01) improves the *Q*-value compared to raw fMRI data. In contrast, the *Q*-value for *mot12*-processed data (gray dots in Figure 4) is not significantly (paired *t*-test, *p* > 0.05) different from the value for raw data.

**FIGURE 5:**
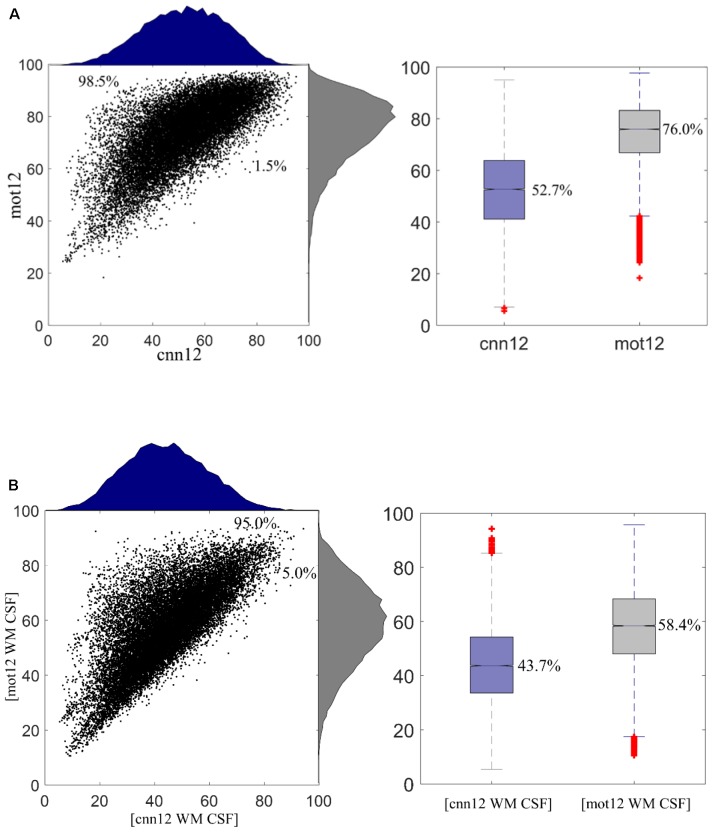
**(A)** Remaining variance of regional time series after motion regression. 98.5% ROI time series after *mot12* motion regression have a larger variance than using *cnn12* motion regression. The median percentage of remaining variance for *cnn12* and *mot12* regression are 52.7 and 76.0%, respectively. **(B)** Remaining variance of regional time series after nuisance regression with average WM and CSF time series as additional regressors. 95.0% ROI time series using [*mot12* WM CSF] regression have a larger variance than using [*cnn12* WM CSF] regression. The median percentage of remaining variance for *cnn12* and *mot12* regression are 43.7 and 58.4%, respectively.

Figure 5A shows the remaining variance (in %) of regional time series after motion regressing using *cnn12* or *mot12*. This plot is generated with all data from 76 subjects. The histograms for *cnn12* and *mot12* are shown in blue and gray color, respectively. The remaining variance of *cnn12*-processed time series is significantly lower than the remaining variance of *mot12*-processed time series with *p* < 10^-4^. 98.5% of *cnn12*-processed time series have remaining variance lower than the corresponding time series regressed by *mot12*. The median percentages of variance retained for *cnn12* and *mot12* were 52.7 and 76.0%, respectively. In addition, we have also computed the remaining variance by including average time series within WM or CSF masks as additional regressors (Figure 5B). Consistent with the finding described above, the time series regressed with [*cnn12* WM CSF] have remaining variance significantly (*p* < 10^-4^) less than the corresponding time series regressed with [*mot12* WM CSF]. The median percentage of remaining variance for *cnn12* and *mot12* with average WM and CSF time series as regressors are 43.7 and 58.4%, respectively.

**FIGURE 6:**
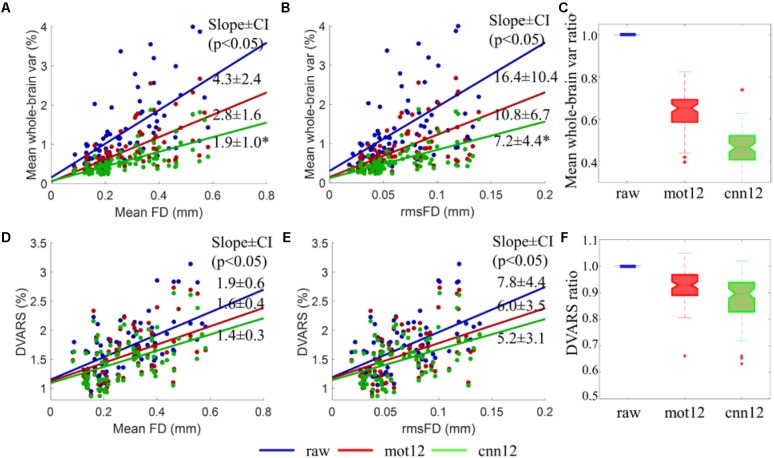
Comparison of different motion regressors using *mean whole-brain variance* and DVARS of intensity-normalized BOLD signal. **(A,B,D,E)** Scatter plots of fMRI data derived measurements (mean whole-brain variance and DVARS) versus quality control measurements (mean FD and rmsFD). The mean whole-brain variance is computed by first converting fMRI time series to percent signal change and then calculating the mean of the variance of all voxels time series across the entire brain. **(C,F)** Boxplots of the ratio of mean whole-brain variance and DVARS. The ratios of mean whole-brain variance and DVARS are defined as the ratio of the measurements after motion regression over the value before motion regression. Data from all 76 subjects are used for the analysis. The slopes of the trend with 95% confidence interval between mean whole-brain standard deviation and mean FD or rmsFD are listed in the figure.

As shown in Figure 6A,B, the mean whole-brain variance for raw fMRI data is observed to have a significant (*p* < 0.05) positive linear relationship with FD (see Figure 6A blue dots, slope ± 95% confidence interval (CI): 4.3 ± 2.4) and rmsFD (see Figure 6B blue dots, slope ± CI: 16.4 ± 10.4). Thus, a reduction of the mean whole-brain variance after motion regression can be treated as a measurement derived from fMRI data itself to evaluate the improvement of applying different motion regressors. The linear relationship between mean whole-brain variance and quality control measurements including FD and rmsFD suggests that greater reduction of motion artifacts is expected to have weaker linear dependency between the variance and motion measurements, and lower mean whole-brain variance value. Using *mot12* regressors, the trend of mean whole-brain variance with mean FD and rmsFD is reduced to 2.8 ± 1.6 and 10.8 ± 6.7, respectively. Using *cnn12* regressors, the trend of mean whole-brain variance with mean FD and rmsFD is reduced to 1.9 ± 1.0 and 7.2 ± 4.4, respectively. The slope of *cnn12* is significantly (*p* < 0.01) flatter than the slope of *mot12* in the linear relationship between mean whole-brain variance and FD or rmsFD. Boxplot of mean whole-brain variance ratio for different motion regressors are shown in Figure 6C. Mean whole-brain variance ratio for a single subject is defined as the ratio of the variance after motion regression over the variance before motion regression. Naturally, the ratio of raw fMRI data (only processed with general preprocessing steps) is equal to one for all subjects. A ratio less than one indicates that the mean whole-brain variance is reduced in comparison to raw fMRI data. Both *mot12* (median ratio 0.65) and *cnn12* (median ratio 0.47) have a ratio lower than the value for raw data, and the ratio for *cnn12* is significantly (*p* < 10^-4^) lower than the ratio for *mot12*.

Similar to the mean whole-brain variance, the DVARS for fMRI data without any additional preprocessing steps also has a significant (*p* < 0.01) positive linear relation with motion measurements including FD (see Figure 6D blue dots, slope ± CI: 1.9 ± 0.6) and rmsFD (see Figure 6E blue dots, slope ± CI: 7.8 ± 4.4). Both *mot12* (slope ± CI with FD: 1.6 ± 0.4; slope ± CI with rmsFD: 6.0 ± 3.5) and *cnn12* (slope ± CI with FD: 1.4 ± 0.3; slope ± CI with rmsFD: 5.2 ± 3.1) decrease the dependency on quality control measurements. The *cnn12* method achieves the weakest linear relationship but the change of slope does not pass a significance level of *p* < 0.05. Boxplots of DVARS ratio for different motion regressors are shown in Figure 6F. DVARS ratio for a single subject is defined as the ratio of the mean DVARS across time points after motion regression over the mean value before motion regression. Both *mot12* (median ratio 0.93) and *cnn12* (median ratio 0.89) overall have reduced DVARS values. Furthermore, *cnn12* has a DVARS ratio significantly (*p* < 10^-4^) less than *mot12*.

**FIGURE 7:**
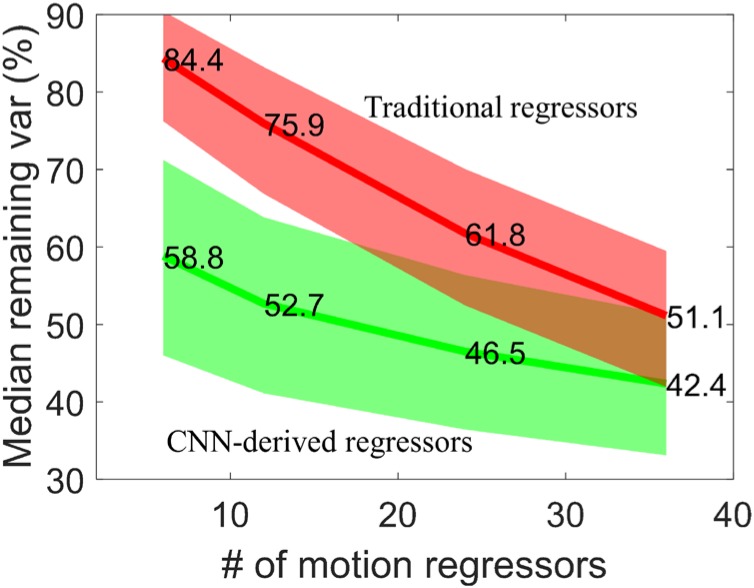
Median percentage of remaining variance with 6, 12, 24, and 36 motion regressors. The 25th and 75th percentiles are used to generate the shaded area for both CNN-derived regressors (green) and traditional regressors (red).

We have also applied the CNN network with ***R*** as input but determined 6, 12, 24, and 36 output regressors. The remaining variance after motion regression is compared with corresponding traditional motion regressors. The detail of these traditional regressors can be seen in section *Motion regressors*. Figure 7 shows the median percentage of remaining variance after regression. Using more regressors naturally explains additional variance and thus leads to less variance remaining. The CNN-derived regressors have a relatively flatter curve and less variance than traditional regressors. Traditional method requires more regressors than the neural network to achieve comparable variance reduction. The traditional method requires 36 regressors (51.1% remaining variance) to have comparable remaining variance with the network with 12 output regressors, namely *cnn12* (52.7% remaining variance). Adding average WM and CSF time series as additional regressors further lowers the remaining variance for both methods, but consistently shows similar difference between CNN-derived and traditional regressors.

**FIGURE 8:**
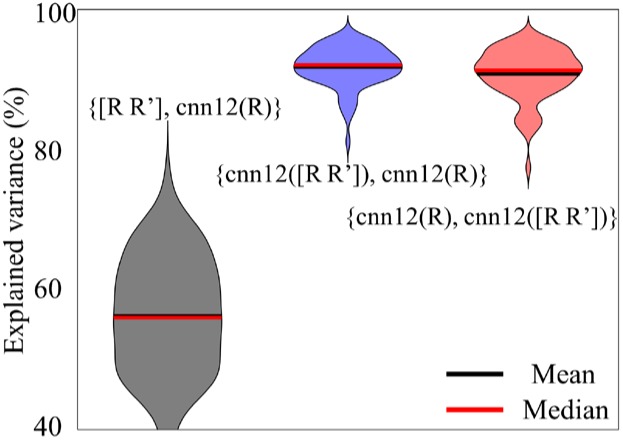
Violin plots of the variance explained (in %) between different sets of regressors. The notation {regressor set 1, regressor set 2} denotes the variance explained for each of the regressors in set 2 by the matrix of regressors in set 1 by linear regression and averaged for all regressors in set 2.

One interesting question for *cnn12* is whether more *input* motion regressors are beneficial for the output regressors. We have computed the percentage of variance in regressor set 2 explained by regressor set 1 using the notation {regressor set 1, regressor set 2}. For the pair {regressor set 1, regressor set 2}, the variance explained for each of the regressors in set 2 by the matrix of regressors in set 1 is computed by linear regression and averaged for all regressors in set 2. The explained variance (in %) is used for the violin plot in Figure 8. In all analyses above, the input data to the neural network are the 6 motion parameters ***R***, namely *cnn12*(***R***), and the input is omitted for simplicity. In this part, we have also applied the neural network with the derivative of motion parameters ***R*** as additional input, namely *cnn12*([***R*** ***R***’]), and all the other settings are exactly the same as in *cnn12*(***R***). As shown in Figure 8, *cnn12*([***R*** ***R***’]) explained the variance of *cnn12*(***R***) (blue) with a median and mean percentage more than 90%, and vice versa (red). In contrast, [***R*** ***R***’] could only explain about 56% of variance in *cnn12*(***R***). Furthermore, *cnn12*-derived regressors have a mean correlation of 0.65 with raw motion time series across all subjects and a mean correlation of 0.47 with mask-averaged WM and CSF time series.

## Discussion

Motion-related artifacts are a major problem in the analysis of rs-fMRI data. Modeling and reducing these artifacts are critical for improving fMRI analysis. In this study, we have designed a CNN framework for modeling rigid-body motion artifacts in rs-fMRI data. To the best of our knowledge, this is the first study where a deep neural network is designed for denoising resting-state functional MRI data. The proposed subject-level CNN model is constructed with two sequential 1-dim temporal convolutional layers. With the assumption that the voxels within WM or CSF masks share similar motion-related fluctuation as the voxels in GM mask but do not contain any BOLD signal of neural origin, the time series used in the CNN network are limited to voxel locations within the non-GM mask to avoid BOLD signal modeled erroneously in the output regressors. The estimated motion parameters during rigid-body realignment are replicated to match the number of non-GM voxels and then each repetition is treated as a sample to optimize the CNN model. The correlation between non-GM time series and output regressors is used to compute the loss function for optimizing the parameters in the model. The 12-regressor CNN network, *cnn12*, is compared with traditional motion regression, namely *mot12*, for data from 76 subjects downloaded from the ADNI database. While *cnn12* and *mot12* have the same number of regressors, *cnn12* takes advantage of the flexibility in the network to model signal disruption of rigid-body head movements without prior assumptions. The proposed *cnn12* was shown to be superior to *mot12* in terms of multiple quantitative measurements.

### High-Motion and Low-Motion Groups

Two prominent effects of motion are the increase of pairwise correlations for nearby voxels and the increase of whole-brain correlations if the signal disruption is widespread and similar over the entire brain (Power et al., 2015). Consistent with these two effects, the high-motion group has more significant linear relationship with Euclidean distance between ROIs, and higher FC than the low-motion group (see Figure 3A). With the assumption that signal disruption is more severe in the high-motion group, the difference between the high-motion and the low-motion groups can be explained by these two effects. These findings suggest that motion artifacts considerably influence the analysis and interpretation of fMRI data. Considering the distance dependent FC, a larger slope of Δ*r* as a function of the inter-ROI distance indicates that motion regression is more effective in reducing motion-related fluctuations. The negative intercept is a sign of decreased correlations. Since the high-motion group is more severely affected by motion-related artifacts, the two techniques including *mot12* and *cnn12*, as expected, have a steeper slope and smaller intercept (in magnitude) for the plot of Δ*r* versus distance. However, *cnn12* has significantly reduced motion-related artifacts compared to *mot12*, in terms of slope and intercept, for both high- and low-motion groups.

We would like to point out that both *cnn12* and *mot12* can only reduce but not completely remove motion-related artifacts. After motion regression, the FC in denoised data is still strongly associated with Euclidean distance between ROIs. A fixed set of motion regressors for the entire brain can only partially explain motion artifacts because of the potential variability of motion artifacts across voxels. Multiple studies have demonstrated that motion regression should be applied together with other processing steps to further reduce signal contamination. For example, Patel et al. (2014) applied wavelet- or time-domain de-spiking before nuisance regression and Power et al. (2012) proposed a “scrubbing” technique to remove motion-related spikes as a complementary strategy to motion regression. The CNN-derived regressors can also be combined with these denoising strategies by simply replacing traditional motion regressors with the derived regressors to further reduce the influence of head motion. Voxel-specific motion parameters (Wilke, 2012; Yan et al., 2013) potentially can also be combined with *cnn12* regressors to further reduce artifacts.

### Further Comparison of *mot12* and *cnn12*

Multiple studies indicate that neurobiological signals in human fMRI data only occupy 5–20% of signal variance (Bianciardi et al., 2009; Marcus et al., 2013). We observe that the remaining variance by *mot12* is significantly higher than *cnn12*. More than 90% of *mot12*-regressed time series have variance higher than *cnn12*-regressed time series regardless whether additional tissue-based regressors are used. The median percentage of remaining variance for *cnn12* is 23.3% less than *mot12*, and the variance difference is reduced to 14.7% less than *mot12* if average WM and CSF time series are also included for regression. The decreased variance difference may be because tissue-based regressors share more common information with *cnn12* but less with *mot12* regressors. We have applied motion regression with 6, 12, 24, and 36 regressors. The CNN-derived regressors always explain more variance than traditional regressors, leading to less remaining variance. Even though *cnn12* removes more variance than *mot12, cnn12* remains a higher network modularity. Considering that the modularity quality is expected to decline if BOLD signal is removed along with noise in motion regression, this finding suggests that the extra variance removed by *cnn12* is more likely to be motion-related noise instead of the BOLD signal.

Both mean whole-brain variance and DVARS are measurements computed from fMRI data itself to evaluate the influence of motion regression. The positive linear relationship in Figure 6 shows that the magnitude of these two measurements are heavily driven by head movement. The weaker linear relationship with FD or rmsFD, and smaller value of these two measurements indicate improved data quality. Compared to *mot12, cnn12* had significantly flatter slope between mean whole-brain variance and FD or rmsFD. While the slope difference between *cnn12* and *mot12* is not significant, *cnn12* still achieved the flattest slope between DVARS and FD or rmsFD. For both mean whole-brain variance and DVARS, *cnn12* achieves a ratio value less than *mot12*. The weaker linear relationship and smaller ratio value consistently suggest that *cnn12* outperforms *mot12*.

### Potential Modification of the Network

As mentioned in section *Architecture of CNN network*, the realignment parameters are replicated and paired with different time series within non-GM mask to form a large number of samples for optimizing the designed CNN network, as demonstrated in Figure 1. One potential way to modify the network is to switch the time series and parameters ***R*** in the CNN network and thus output voxel-wise motion regressors instead of volume-wise motion regressors. In other words, non-GM time series are used as input data and along with ***R*** to compute the loss function and thus voxel-specific motion regressors could be obtained with such a revised network. The CNN network is highly flexible because of the large amount of model parameters in the network, however, the flexibility can be beneficial or detrimental to the following analysis depending on the input. The flexibility in the alternative neural network can easily make the output of arbitrary input time series highly correlated with parameters ***R*** to achieve optimal loss function but does not extract any useful information. In contrast, the current framework requires the output regressors to optimize the summation of correlations over all non-GM voxels. Output regressors that are highly correlated with a single time series are not optimal because they have a minor effect in the loss function due to the summation over all voxels. While voxel-specific regressors may be more useful than a fixed set of regressors for the entire brain, the current framework with switched time series and realignment parameters has difficulty to extract voxel-specific regressors properly.

The *cnn12* network can also be applied with more motion parameters as input. We have compared the *cnn12* with only ***R*** and with [***R R***’] as input. Interestingly, the space spanned by the *cnn12*(***R***) regressors is similar to the space spanned by the *cnn12*([***R*** ***R***’]) since the variance explained of *cnn12*(***R***) by *cnn12*([***R*** ***R***’]) is larger than 90% for all subjects (see Figure 8). This finding suggests that adding the derivative as input does not have noticeable impact to the output regressors. The unexplained variance maybe due to the intrinsic randomness in the network. However, the *cnn12*(R) regressors can explain a large proportion of variance that cannot be explained by traditional motion regressors [***R*** ***R***’], which may be because motion-related artifacts in fMRI data cannot be sufficiently described by only adding the preceding time point into consideration (Power et al., 2014).

### Novelties of the *cnn12* Network

Compared to standard CNN algorithms, the input and loss function in *cnn12* are specified in a novel way. A standard CNN algorithm requires thousands of samples to train the neural network. Though the *cnn12* network seems to have only the six motion parameters as input samples (which is not the case), we associate each set of motion parameters with different voxel time series in the *cnn12* network. Thus, each motion parameter *paired* with voxel time series is treated as a different sample and, consequently, a sufficient number of samples can be generated to train the neural network.

Many cost functions have been developed for the purpose of classification or regression in machine learning or deep learning applications, such as the mean squared error, mean absolute percentage error, cross entropy, Poisson, and cosine proximity cost functions. These cost functions are calculated with the known true values or classes. However, because the ground truth is unknown, constructing a cost function for *cnn12* denoising faces a significant challenge. To overcome this challenge, we have proposed a customized cost function which does not require knowledge of the true BOLD signal.

### Limitations and Future Study

There are a few limitations in this study. First, similar to most motion regression studies, the same regressors are used for all voxels in the brain. While the revised neural network mentioned in the section above potentially can achieve voxel-specific regressors, unfortunately such a network cannot extract useful information. We would like to explore other neural network architectures for modeling voxel-specific motion in a future study. Second, while this study is only focused on modeling the influence of head motion, other artifact sources such as cardiac and respiratory noise also considerably confound fMRI data analysis. Multiple methods (Glover et al., 2000; Beall, 2010) have been proposed to model cardiac and respiratory fluctuation of fMRI data with the assistance of external recordings, which is not available in the ADNI data. It would be interesting to model these physiologic noise sources by using our neural network with input from external recordings. Third, the hyper-parameters, e.g., filter size, number of nodes, and learning rate, in a network are impacted by the data. The hyper-parameters used in this study are tuned for a single standard EPI sequence. Following studies with a large sample size are required to gain more knowledge about the influence of TR, the number of volumes and EPI sequences, such as multi-echo EPI (Kundu et al., 2012) and multi-band EPI sequences (Moeller et al., 2010). In addition to the motion-related artifacts induced in fMRI data, motion may have a neurobiological basis (Zeng et al., 2014) and could reflect individual differences. Genetic differences and impulsivity were found to be factors related to head motion (Kong et al., 2014; Hodgson et al., 2016). The *positive* motion-BOLD relationship (Yan et al., 2013) may reflect neural origins of motion. Therefore, any approaches for removing motion-related artifacts, including *cnn12*, may remove some useful subject-related information.

While the CNN network is developed based on resting-state data, this technique potentially can also be useful for reducing motion-related artifacts in task-based fMRI data, whereas an additional study with large number of subjects is required for further validation.

## Conclusion

We have proposed a CNN network modeling motion-related signal disruption in rs-fMRI data using estimated realignment parameters and compared the CNN-derived regressors with traditional motion regressors using publicly available data. Visually, *cnn12* is more effective in reducing head-motion effects. Quantitatively, *cnn12* reduces more variance in regional time series, reduces more the trend between motion parameters and other measurements derived from fMRI data itself, makes the data more homogeneous based on between-subject similarity of brain connectivity and leads to a larger modularity Q, when compared to *mot12*.

## Data Availability

Publicly available datasets were analyzed in this study. This data can be found here: http://adni.loni.usc.edu/.

## Author Contributions

ZY and DC conceived and designed the study and acquired, analyzed, and interpreted the data. ZY, XZ, KS, VM, and DC drafted the manuscript, revised the manuscript critically for important intellectual content, and approved the final version of the manuscript to be submitted.

## Conflict of Interest Statement

The authors declare that the research was conducted in the absence of any commercial or financial relationships that could be construed as a potential conflict of interest.

## Footnotes

1 http://adni.loni.usc.edu/

2 http://adni.loni.usc.edu/

3 https://www.fil.ion.ucl.ac.uk/spm/

4 http://stnava.github.io/ANTs/

5 https://keras.io/

6 http://deeplearning.net/software/theano/

## References

[B1] Beall E. B. (2010). Adaptive cyclic physiologic noise modeling and correction in functional MRI. *J. Neurosci. Methods* 187 216–228. 10.1016/j.jneumeth.2010.01.013 20096307

[B2] Beall E. B. Lowe M. J. (2014). SimPACE: generating simulated motion corrupted BOLD data with synthetic-navigated acquisition for the development and evaluation of SLOMOCO: a new, highly effective slicewise motion correction. *Neuroimage* 101 21–34. 10.1016/j.neuroimage.2014.06.038 24969568 PMC4165749

[B3] Behzadi Y. Restom K. Liau J. Liu T. T. (2007). A component based noise correction method (CompCor) for BOLD and perfusion based fMRI. *Neuroimage* 37 90–101. 10.1016/j.neuroimage.2007.04.042 17560126 PMC2214855

[B4] Bianciardi M. Fukunaga M. van Gelderen P. Horovitz S. G. de Zwart J. A. Shmueli K. (2009). Sources of functional magnetic resonance imaging signal fluctuations in the human brain at rest: a 7 T study. *Magn. Reson. Imaging* 27 1019–1029. 10.1016/j.mri.2009.02.004 19375260 PMC3512098

[B5] Blondel V. D. Guillaume J.-L. Lambiotte R. Lefebvre E. (2008). Fast unfolding of communities in large networks. *J. Stat. Mech.* 2008:10008. 21517554

[B6] Caballero-Gaudes C. Reynolds R. C. (2017). Methods for cleaning the BOLD fMRI signal. *Neuroimage* 154 128–149. 10.1016/j.neuroimage.2016.12.018 27956209 PMC5466511

[B7] Ciric R. Wolf D. H. Power J. D. Roalf D. R. Baum G. L. Ruparel K. (2017). Benchmarking of participant-level confound regression strategies for the control of motion artifact in studies of functional connectivity. *Neuroimage* 154 174–187. 10.1016/j.neuroimage.2017.03.020 28302591 PMC5483393

[B8] Courtemanche M. J. Sparrey C. J. Song X. MacKay A. D’arcy R. C. (2018). Detecting white matter activity using conventional 3 Tesla fMRI: an evaluation of standard field strength and hemodynamic response function. *Neuroimage* 169 145–150. 10.1016/j.neuroimage.2017.12.008 29229580

[B9] Friston K. J. Williams S. Howard R. Frackowiak R. S. Turner R. (1996). Movement-related effects in fMRI time-series. *Magn. Reson. Med.* 35 346–355. 10.1002/mrm.1910350312 8699946

[B10] Gawryluk J. R. Mazerolle E. L. Beyea S. D. D’Arcy R. C. (2014). Functional MRI activation in white matter during the symbol digit modalities test. *Front. Hum. Neurosci.* 8:589. 10.3389/fnhum.2014.00589 25136311 PMC4120763

[B11] Glorot X. Bengio Y. (2010). “Understanding the difficulty of training deep feedforward neural networks,” in *Proceedings of the Thirteenth International Conference on Artificial Intelligence and Statistics* Vol. 9 eds Teh Y. W. Titterington M. (Sardinia: Chia Laguna Resort), 249–256.

[B12] Glover G. H. Li T. Q. Ress D. (2000). Image-based method for retrospective correction of physiological motion effects in fMRI: RETROICOR. *Magn. Reson. Med.* 44 162–167. 10.1002/1522-2594(200007)44:1<162::AID-MRM23>3.0.CO;2-E 10893535

[B13] Griffanti L. Salimi-Khorshidi G. Beckmann C. F. Auerbach E. J. Douaud G. Sexton C. E. (2014). ICA-based artefact removal and accelerated fMRI acquisition for improved resting state network imaging. *Neuroimage* 95 232–247. 10.1016/j.neuroimage.2014.03.034 24657355 PMC4154346

[B14] Hodgson K. Poldrack R. A. Curran J. E. Knowles E. E. Mathias S. Göring H. H. (2016). Shared genetic factors influence head motion during MRI and body mass index. *Cereb. Cortex* 27 5539–5546. 10.1093/cercor/bhw321 27744290 PMC6075600

[B15] Jiang A. Kennedy D. N. Baker J. R. Weisskoff R. M. Tootell R. B. Woods R. P. (1995). Motion detection and correction in functional MR imaging. *Hum. Brain Mapp.* 3 224–235. 10.1002/hbm.460030306

[B16] Kim Y. (2014). “Convolutional neural networks for sentence classification,” in *Proceedings of the 2014 Conference on Empirical Methods in Natural Language Processing (EMNLP)* (Doha, Qatar: Association for Computational Linguistics), 1746–1751.

[B17] Kingma D. Ba L. (2015). Adam: a method for stochastic optimization. *arXiv* [Preprint]. arXiv:1412.6980 30066388

[B18] Kong X.-Z. Zhen Z. Li X. Lu H.-H. Wang R. Liu L. (2014). Individual differences in impulsivity predict head motion during magnetic resonance imaging. *PLoS One* 9:e104989. 10.1371/journal.pone.0104989 25148416 PMC4141798

[B19] Krizhevsky A. Sutskever I. Hinton G. E. (2012). Imagenet classification with deep convolutional neural networks. *Adv. Neural Inf. Process. Syst.* 25 1097–1105.

[B20] Kundu P. Inati S. J. Evans J. W. Luh W.-M. Bandettini P. A. (2012). Differentiating BOLD and non-BOLD signals in fMRI time series using multi-echo EPI. *Neuroimage* 60 1759–1770. 10.1016/j.neuroimage.2011.12.028 22209809 PMC3350785

[B21] Lemieux L. Salek-Haddadi A. Lund T. E. Laufs H. Carmichael D. (2007). Modelling large motion events in fMRI studies of patients with epilepsy. *Magn. Reson. Imaging* 25 894–901. 10.1016/j.mri.2007.03.009 17490845

[B22] Liang M. Hu X. (2015). “Recurrent convolutional neural network for object recognition,” in *Proceedings of the IEEE Conference on Computer Vision and Pattern Recognition*, (Piscataway, NJ: IEEE), 3367–3375. 10.1109/CVPR.2015.7298958

[B23] Macey P. M. Macey K. E. Kumar R. Harper R. M. (2004). A method for removal of global effects from fMRI time series. *Neuroimage* 22 360–366. 10.1016/j.neuroimage.2003.12.042 15110027

[B24] Marcus D. S. Harms M. P. Snyder A. Z. Jenkinson M. Wilson J. A. Glasser M. F. (2013). Human Connectome Project informatics: quality control, database services, and data visualization. *Neuroimage* 80 202–219. 10.1016/j.neuroimage.2013.05.077 23707591 PMC3845379

[B25] Moeller S. Yacoub E. Olman C. A. Auerbach E. Strupp J. Harel N. (2010). Multiband multislice GE-EPI at 7 tesla, with 16-fold acceleration using partial parallel imaging with application to high spatial and temporal whole-brain fMRI. *Magn. Reson. Med.* 63 1144–1153. 10.1002/mrm.22361 20432285 PMC2906244

[B26] Murphy K. Birn R. M. Bandettini P. A. (2013). Resting-state fMRI confounds and cleanup. *Neuroimage* 80 349–359. 10.1016/j.neuroimage.2013.04.001 23571418 PMC3720818

[B27] Murphy K. Birn R. M. Handwerker D. A. Jones T. B. Bandettini P. A. (2009). The impact of global signal regression on resting state correlations: are anti-correlated networks introduced? *Neuroimage* 44 893–905. 10.1016/j.neuroimage.2008.09.036 18976716 PMC2750906

[B28] Nair V. Hinton G. E. (2010). “Rectified linear units improve restricted boltzmann machines,” in *Proceedings of the 27th International conference on machine learning (ICML-10)*, eds Fürnkranz J. Joachims T. (New York, NY: ACM), 807–814.

[B29] Patel A. X. Kundu P. Rubinov M. Jones P. S. Vértes P. E. Ersche K. D. (2014). A wavelet method for modeling and despiking motion artifacts from resting-state fMRI time series. *Neuroimage* 95 287–304. 10.1016/j.neuroimage.2014.03.012 24657353 PMC4068300

[B30] Power J. D. Barnes K. A. Snyder A. Z. Schlaggar B. L. Petersen S. E. (2012). Spurious but systematic correlations in functional connectivity MRI networks arise from subject motion. *Neuroimage* 59 2142–2154. 10.1016/j.neuroimage.2011.10.018 22019881 PMC3254728

[B31] Power J. D. Cohen A. L. Nelson S. M. Wig G. S. Barnes K. A. Church J. A. (2011). Functional network organization of the human brain. *Neuron* 72 665–678. 10.1016/j.neuron.2011.09.006 22099467 PMC3222858

[B32] Power J. D. Mitra A. Laumann T. O. Snyder A. Z. Schlaggar B. L. Petersen S. E. (2014). Methods to detect, characterize, and remove motion artifact in resting state fMRI. *Neuroimage* 84 320–341. 10.1016/j.neuroimage.2013.08.048 23994314 PMC3849338

[B33] Power J. D. Plitt M. Gotts S. J. Kundu P. Voon V. Bandettini P. A. (2018). Ridding fMRI data of motion-related influences: removal of signals with distinct spatial and physical bases in multiecho data. *Proc. Natl. Acad. Sci. U.S.A.* 115 E2105–E2114. 10.1073/pnas.1720985115 29440410 PMC5834724

[B34] Power J. D. Schlaggar B. L. Petersen S. E. (2015). Recent progress and outstanding issues in motion correction in resting state fMRI. *Neuroimage* 105 536–551. 10.1016/j.neuroimage.2014.10.044 25462692 PMC4262543

[B35] Pruim R. H. Mennes M. van Rooij D. Llera A. Buitelaar J. K. Beckmann C. F. (2015). ICA-AROMA: a robust ICA-based strategy for removing motion artifacts from fMRI data. *Neuroimage* 112 267–277. 10.1016/j.neuroimage.2015.02.064 25770991

[B36] Saad Z. S. Gotts S. J. Murphy K. Chen G. Jo H. J. Martin A. (2012). Trouble at rest: how correlation patterns and group differences become distorted after global signal regression. *Brain Connect.* 2 25–32. 10.1089/brain.2012.0080 22432927 PMC3484684

[B37] Satterthwaite T. D. Elliott M. A. Gerraty R. T. Ruparel K. Loughead J. Calkins M. E. (2013). An improved framework for confound regression and filtering for control of motion artifact in the preprocessing of resting-state functional connectivity data. *Neuroimage* 64 240–256. 10.1016/j.neuroimage.2012.08.052 22926292 PMC3811142

[B38] Satterthwaite T. D. Wolf D. H. Loughead J. Ruparel K. Elliott M. A. Hakonarson H. (2012). Impact of in-scanner head motion on multiple measures of functional connectivity: relevance for studies of neurodevelopment in youth. *Neuroimage* 60 623–632. 10.1016/j.neuroimage.2011.12.063 22233733 PMC3746318

[B39] Smyser C. D. Inder T. E. Shimony J. S. Hill J. E. Degnan A. J. Snyder A. Z. (2010). Longitudinal analysis of neural network development in preterm infants. *Cereb. Cortex* 20 2852–2862. 10.1093/cercor/bhq035 20237243 PMC2978240

[B40] Van Dijk K. R. Sabuncu M. R. Buckner R. L. (2012). The influence of head motion on intrinsic functional connectivity MRI. *Neuroimage* 59 431–438. 10.1016/j.neuroimage.2011.07.044 21810475 PMC3683830

[B41] Weissenbacher A. Kasess C. Gerstl F. Lanzenberger R. Moser E. Windischberger C. (2009). Correlations and anticorrelations in resting-state functional connectivity MRI: a quantitative comparison of preprocessing strategies. *Neuroimage* 47 1408–1416. 10.1016/j.neuroimage.2009.05.005 19442749

[B42] Wilke M. (2012). An alternative approach towards assessing and accounting for individual motion in fMRI timeseries. *Neuroimage* 59 2062–2072. 10.1016/j.neuroimage.2011.10.043 22036679

[B43] Yan C.-G. Cheung B. Kelly C. Colcombe S. Craddock R. C. Di Martino A. (2013). A comprehensive assessment of regional variation in the impact of head micromovements on functional connectomics. *Neuroimage* 76 183–201. 10.1016/j.neuroimage.2013.03.004 23499792 PMC3896129

[B44] Zeng L.-L. Wang D. Fox M. D. Sabuncu M. Hu D. Ge M. (2014). Neurobiological basis of head motion in brain imaging. *Proc. Natl. Acad. Sci. U.S.A.* 111 6058–6062. 10.1073/pnas.1317424111 24711399 PMC4000812

