# Supplementary figures and images for: Robust Motion Regression of Resting-State Data Using a Convolutional Neural Network Model
Source: Front Neurosci. 2019 Feb 28;13:169. doi: 10.3389/fnins.2019.00169 (PMC6482337; doi:10.3389/fnins.2019.00169)

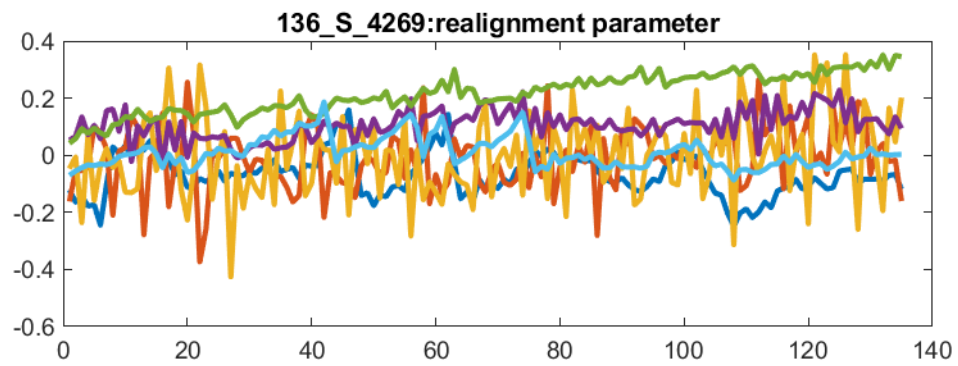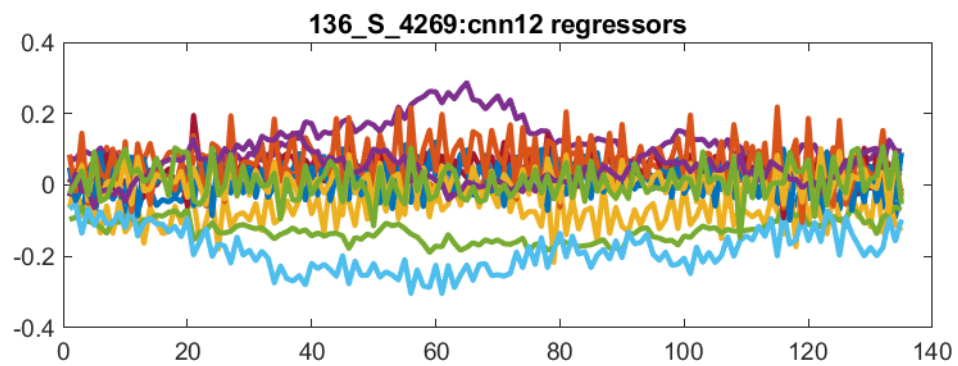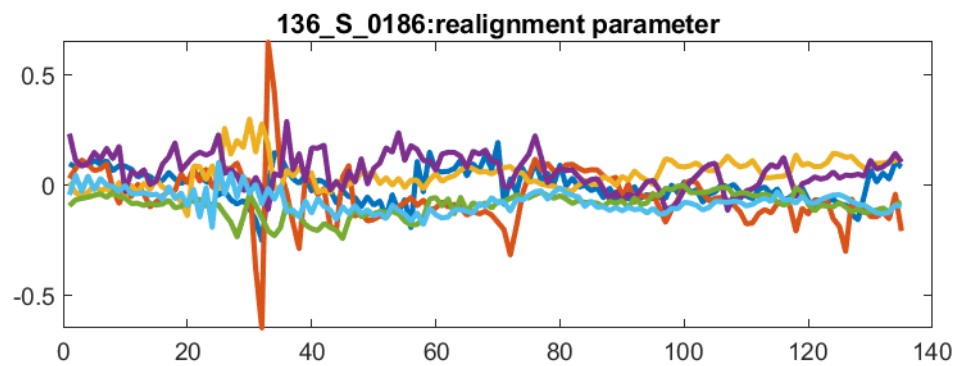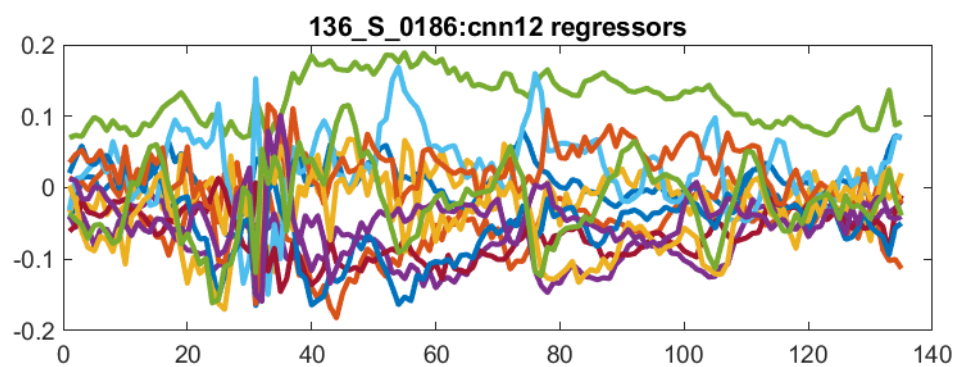

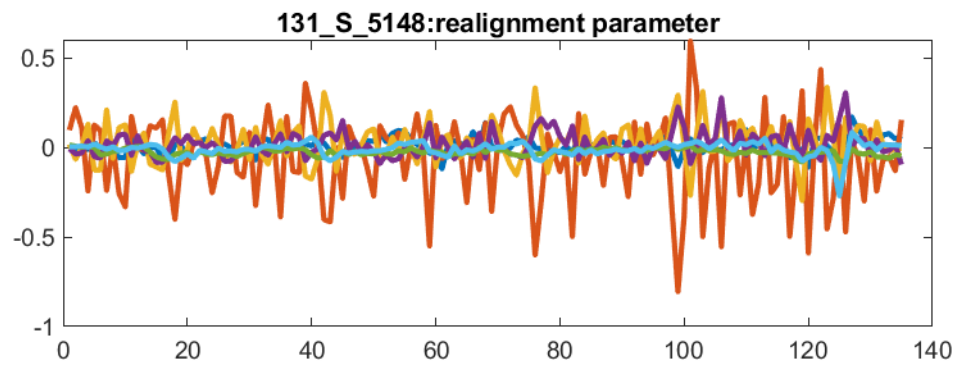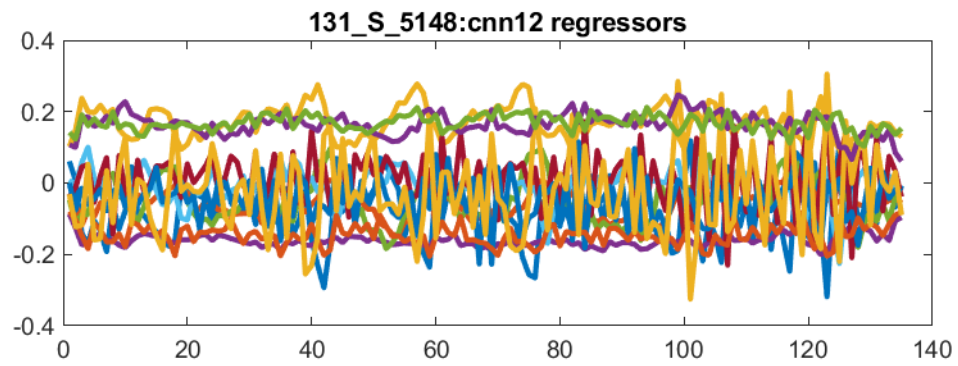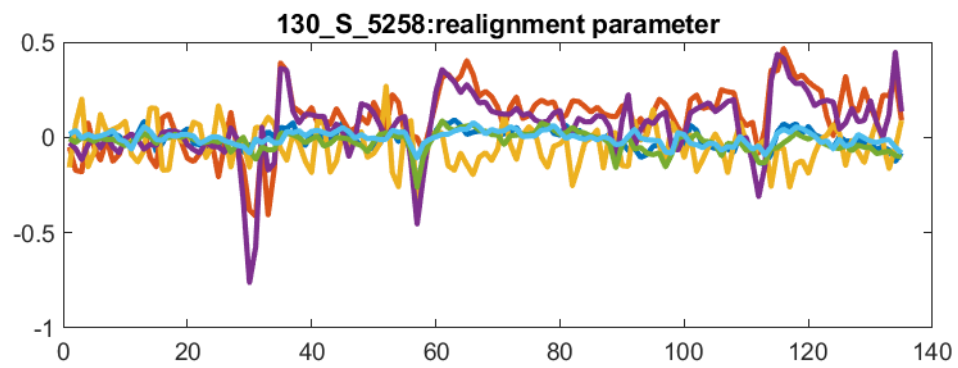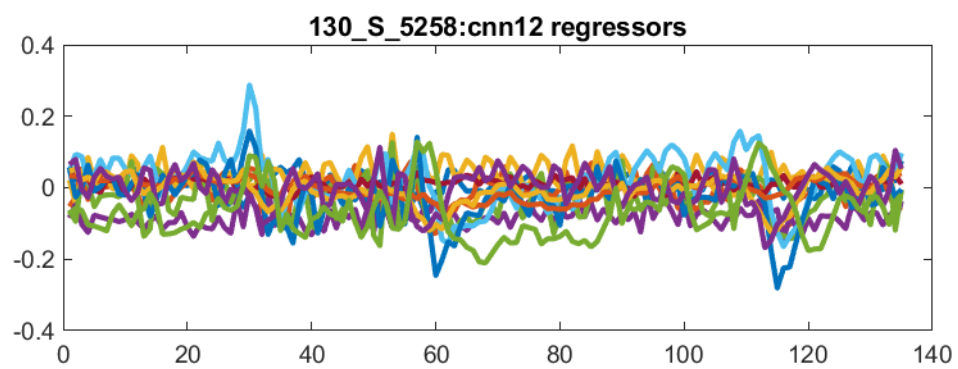

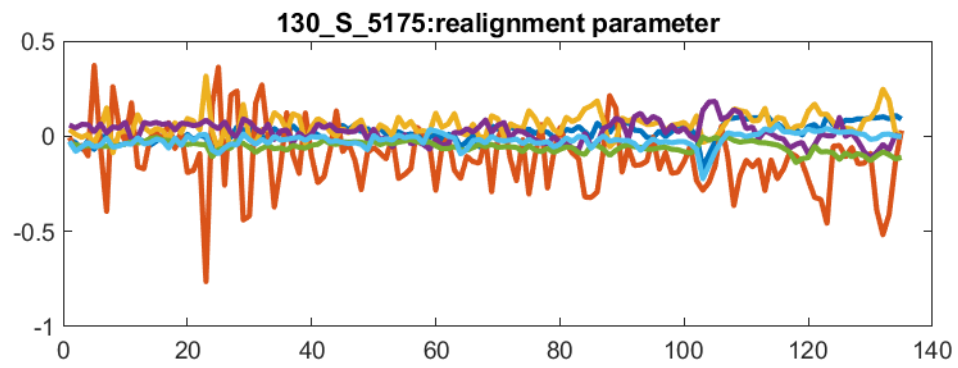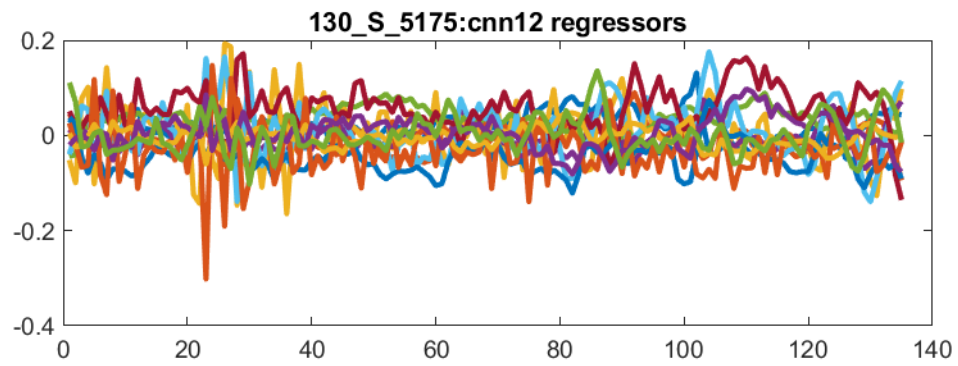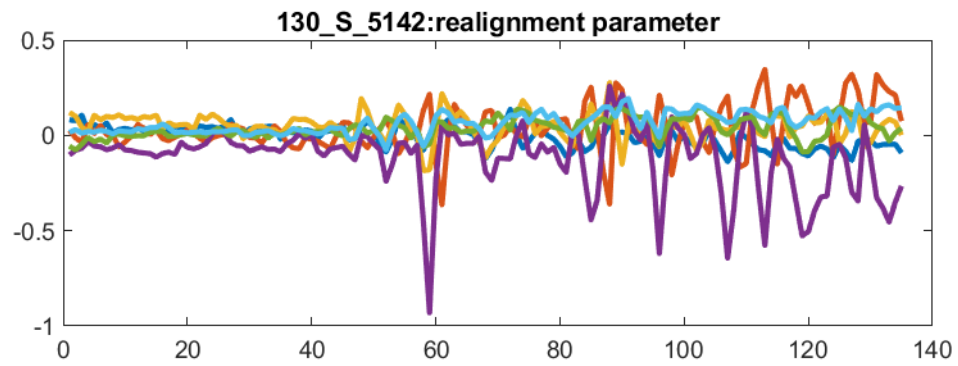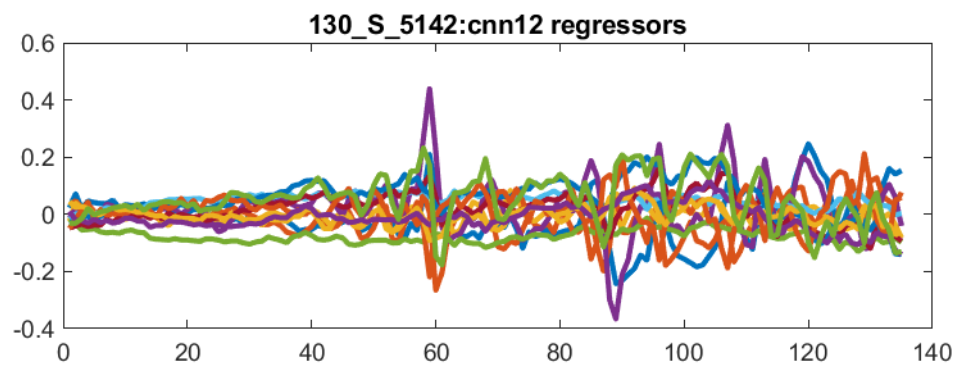

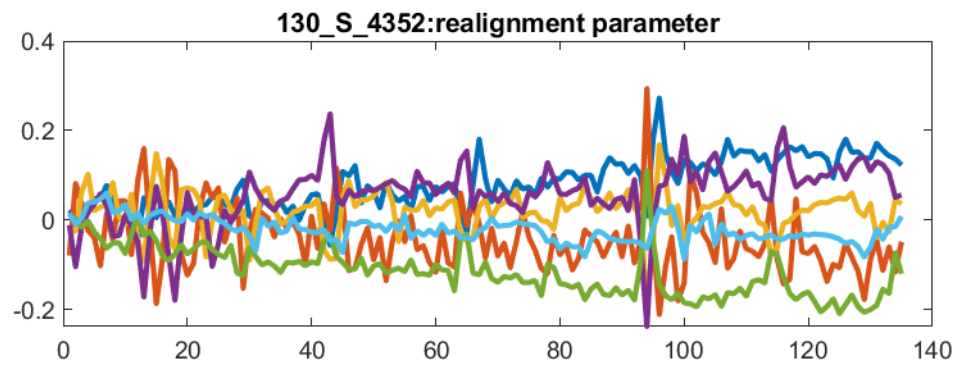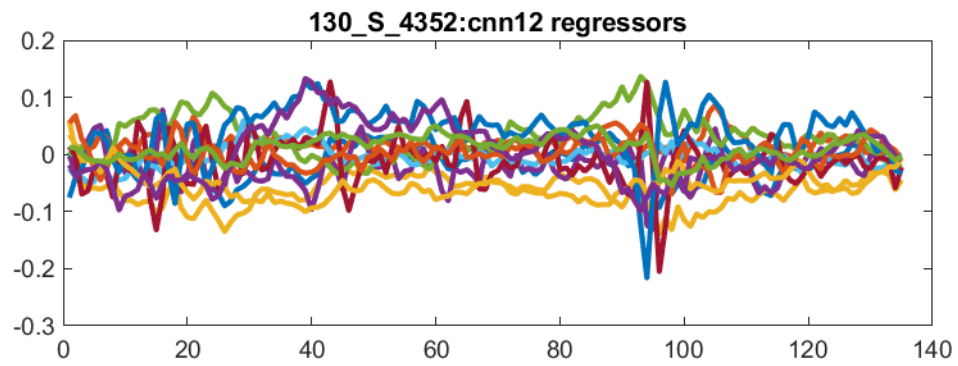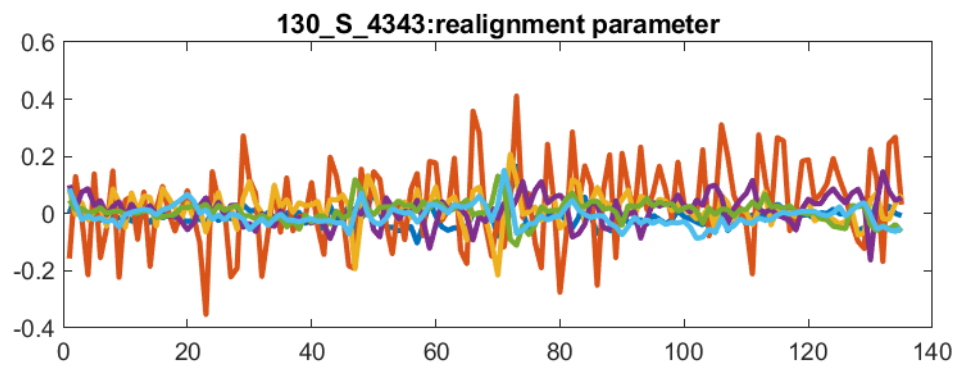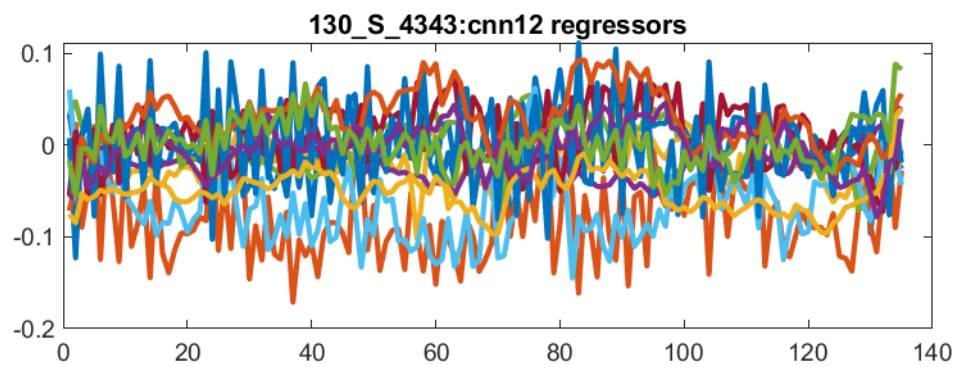

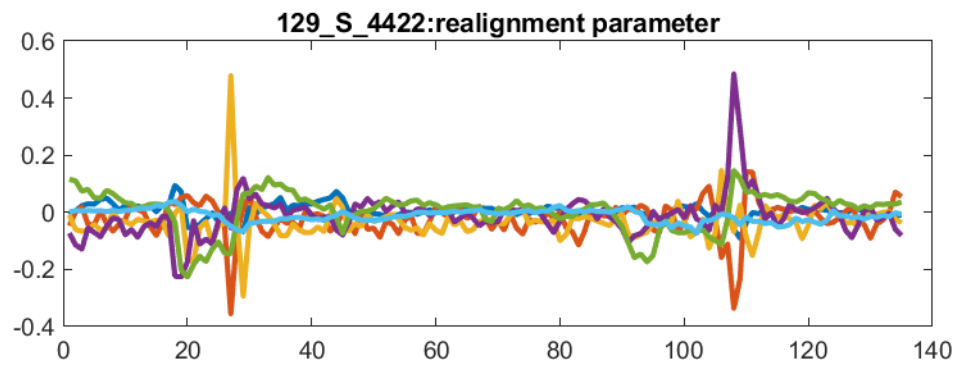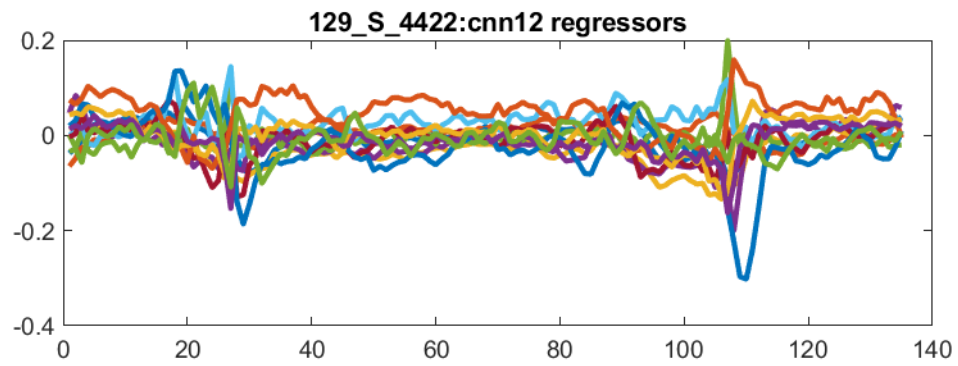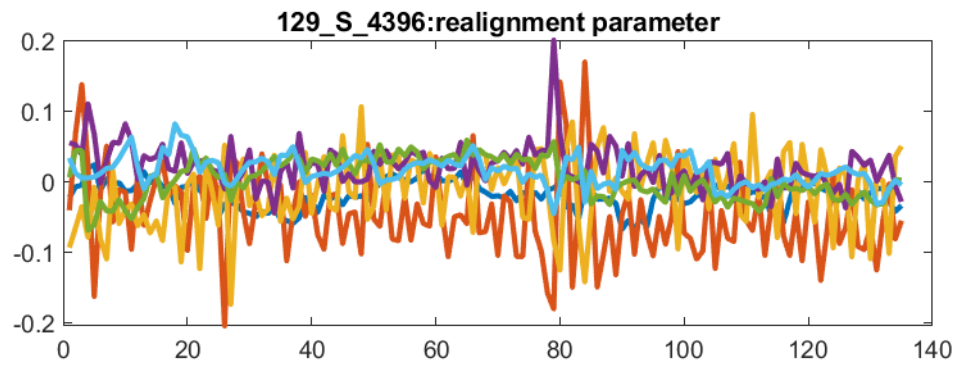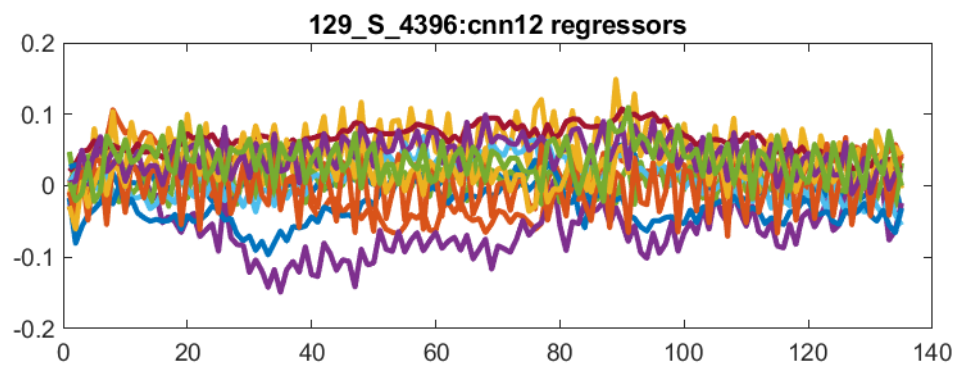

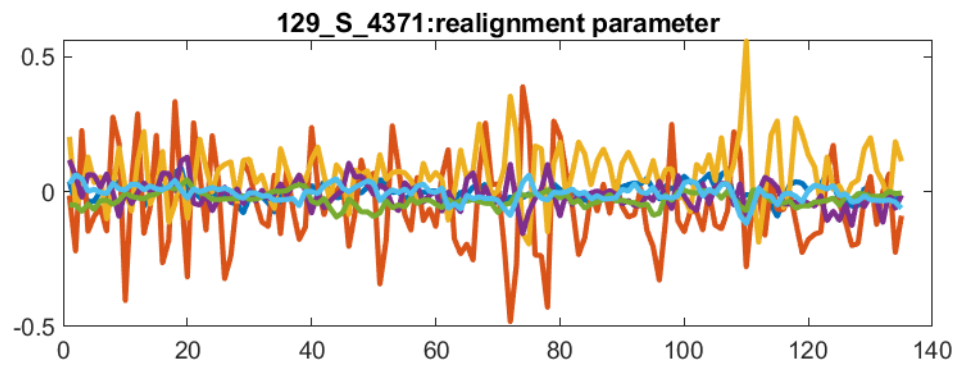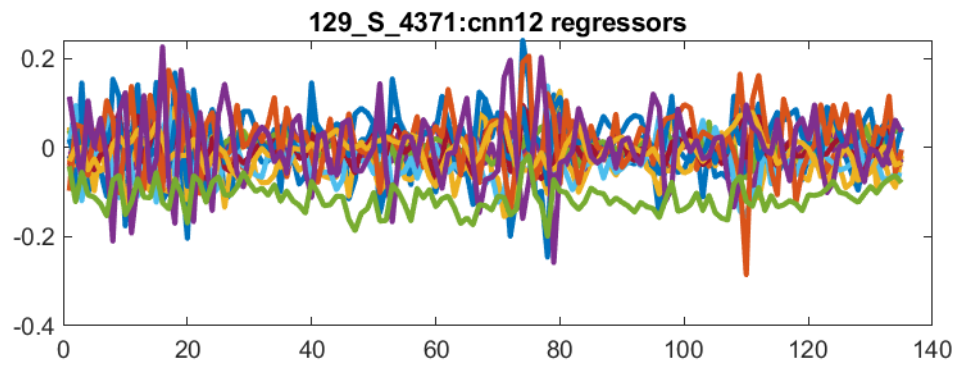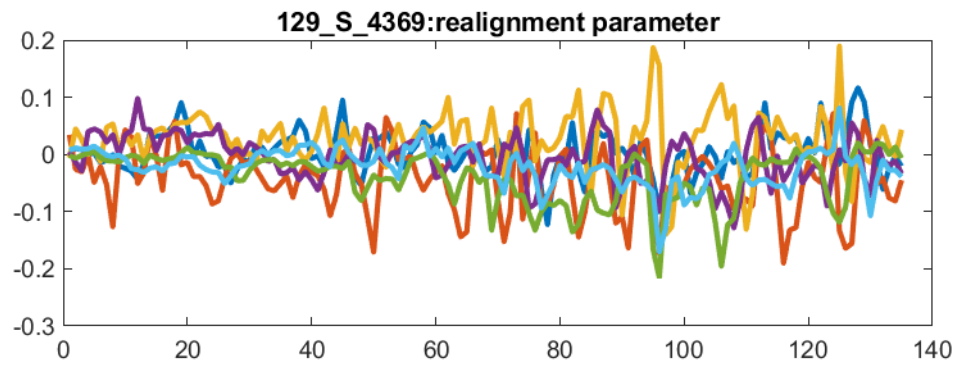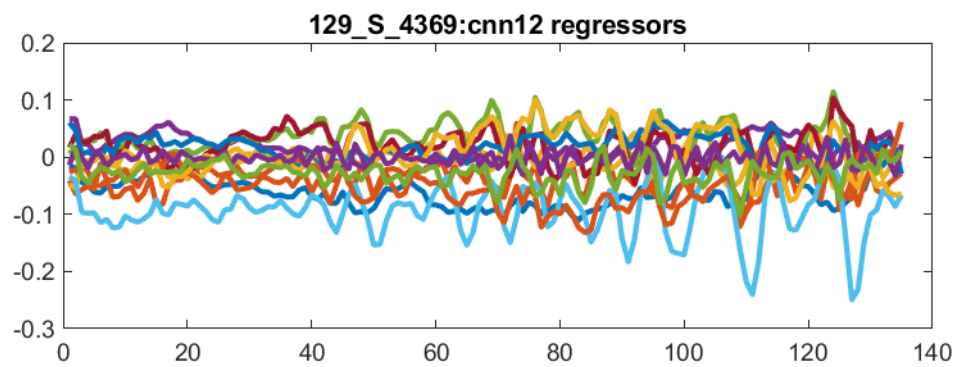

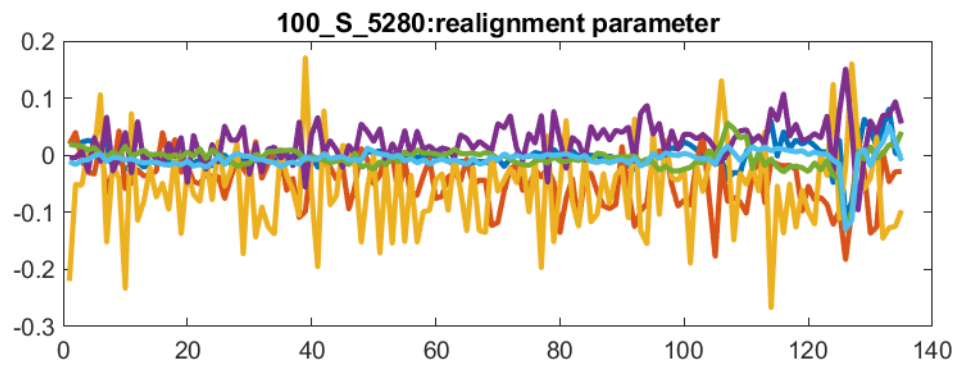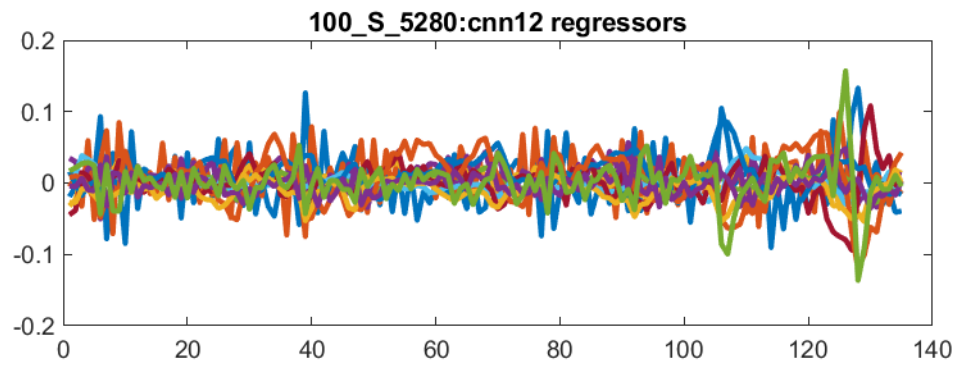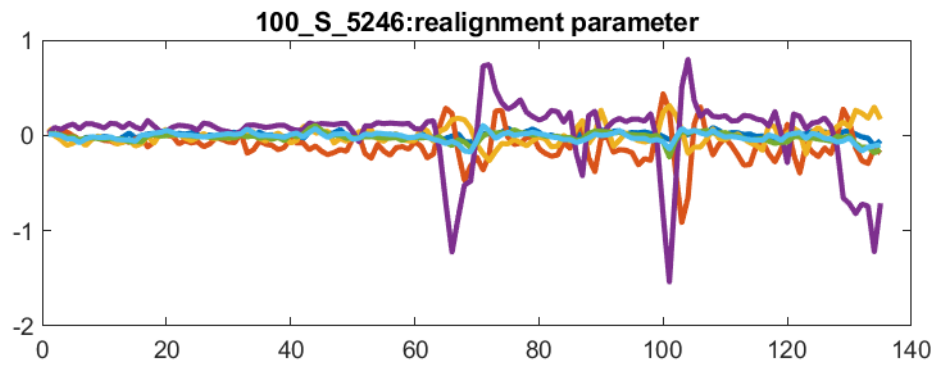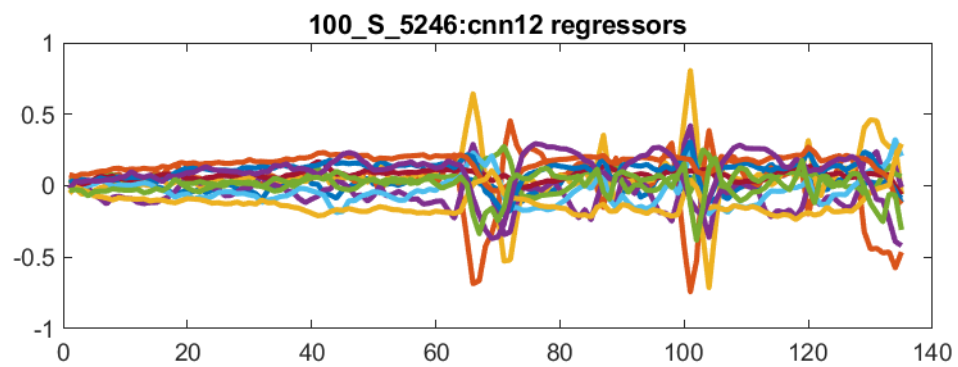

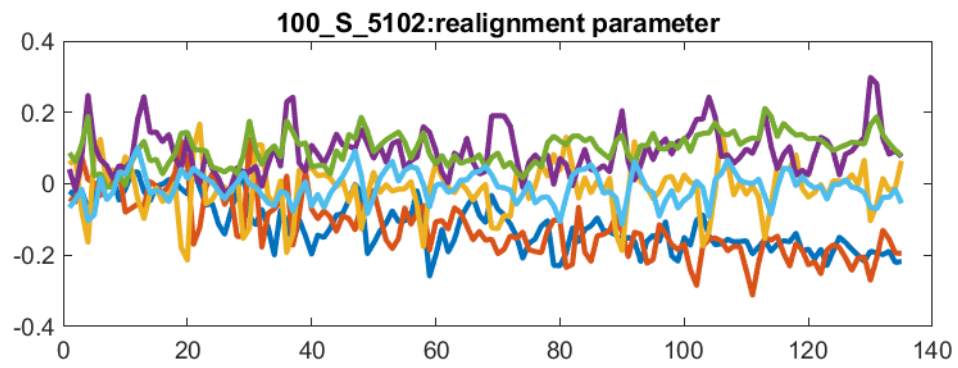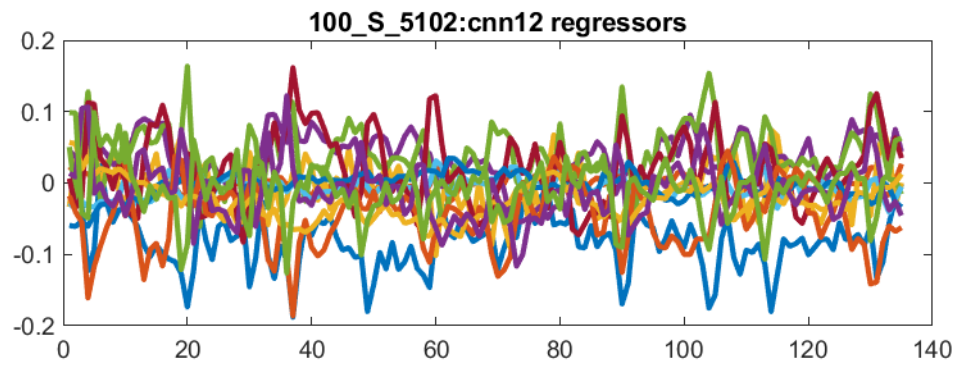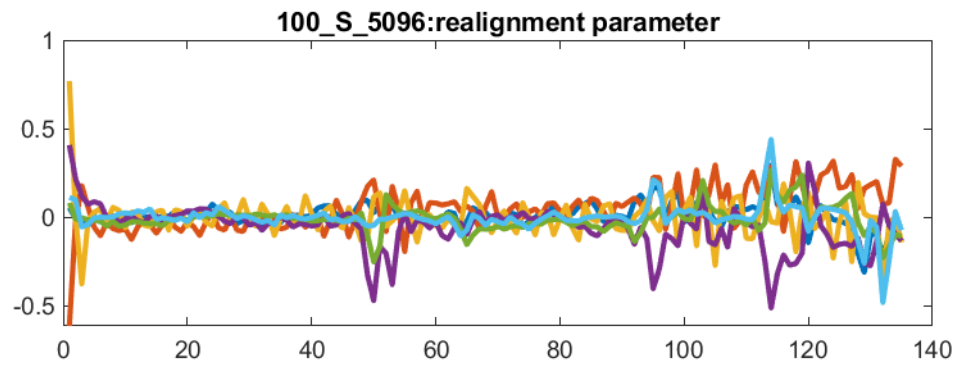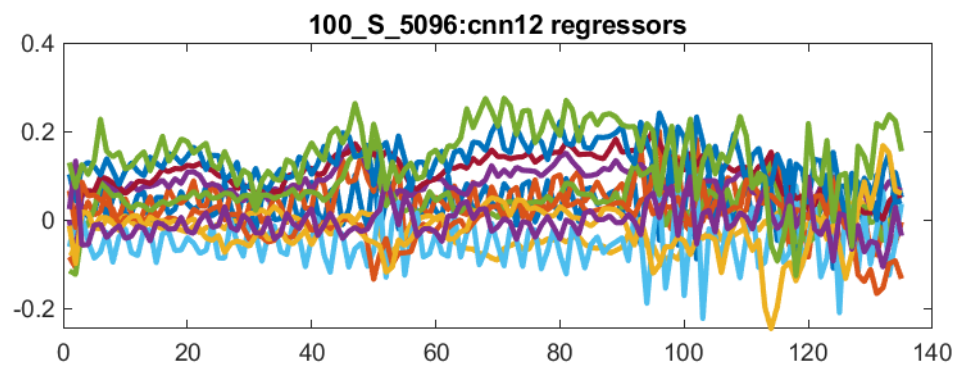

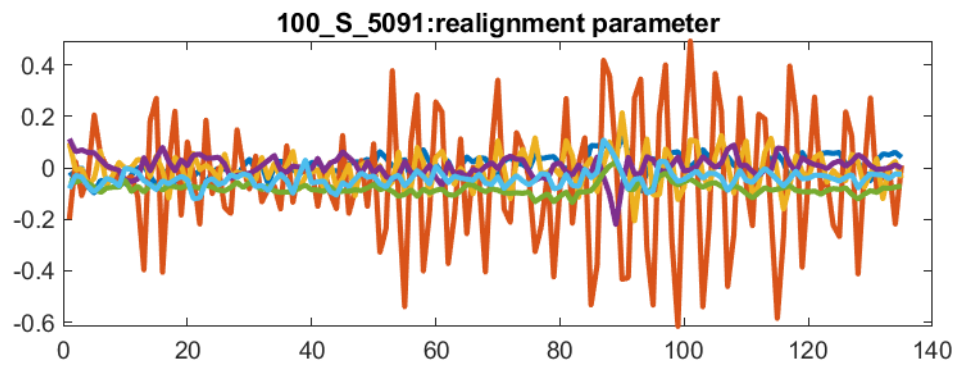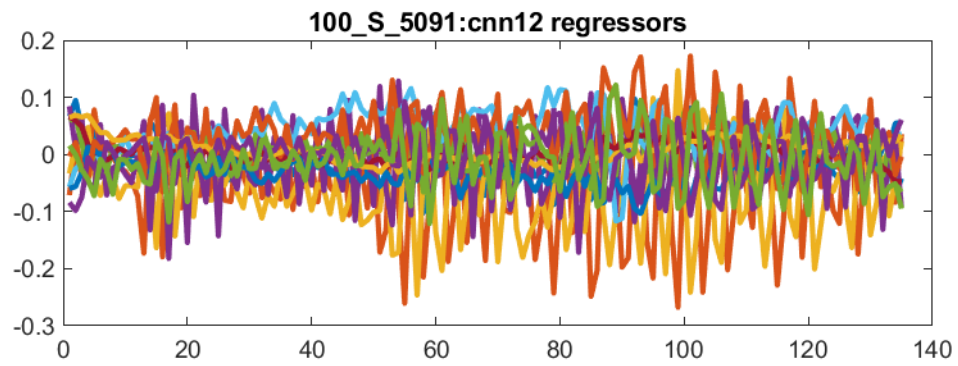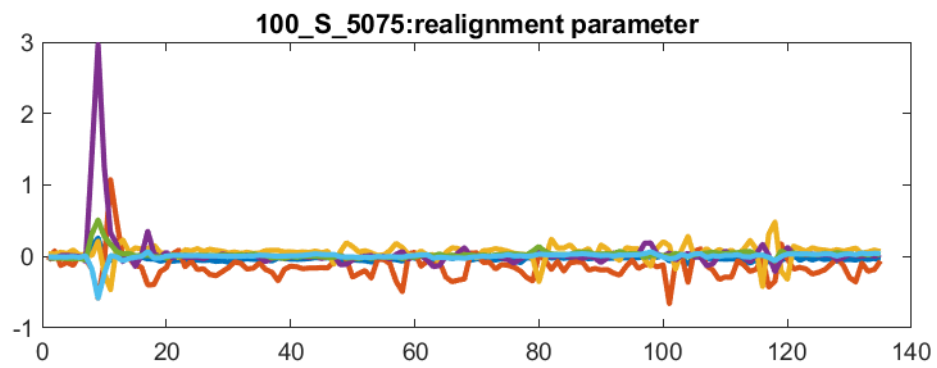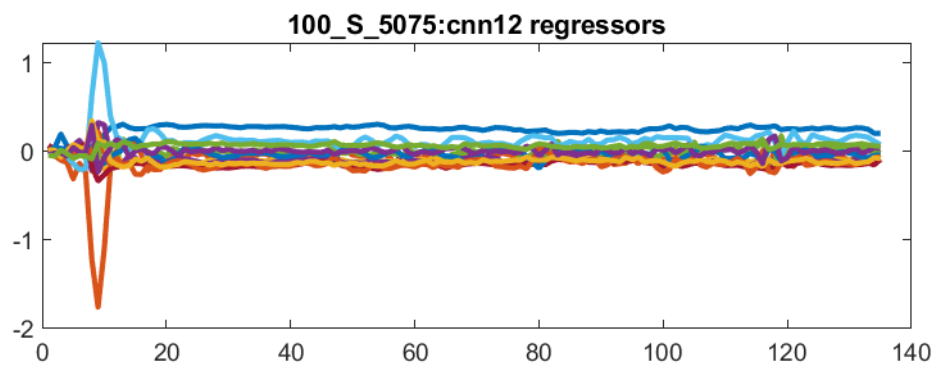

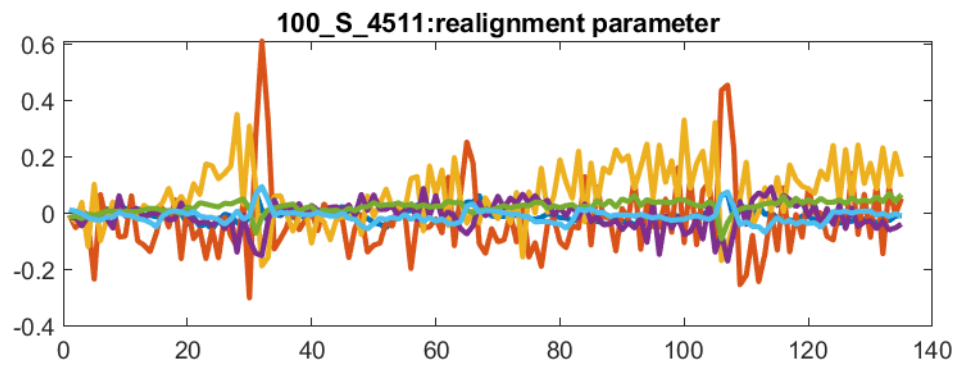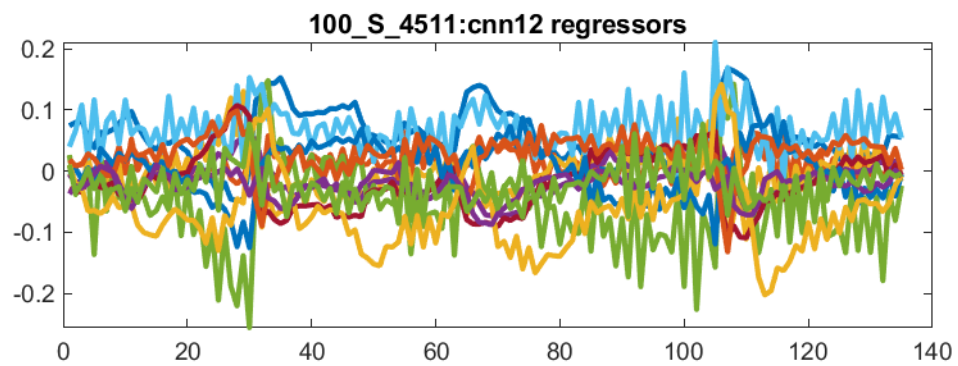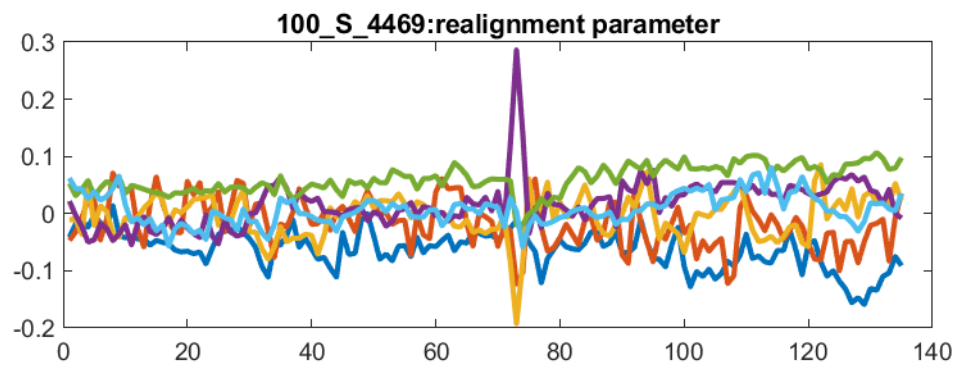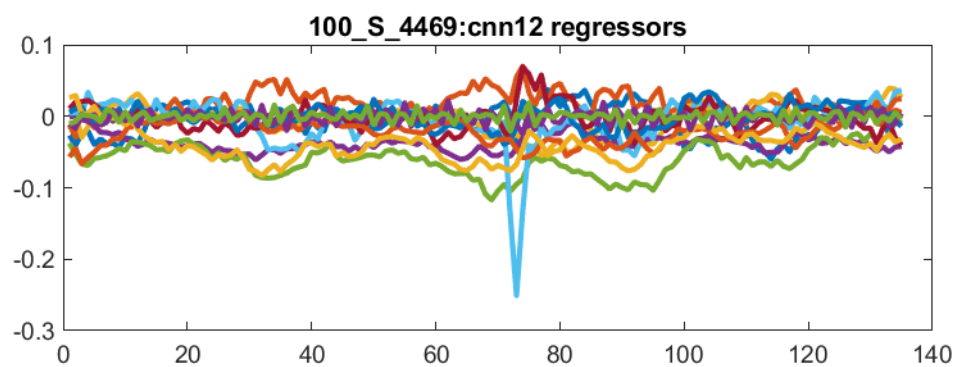

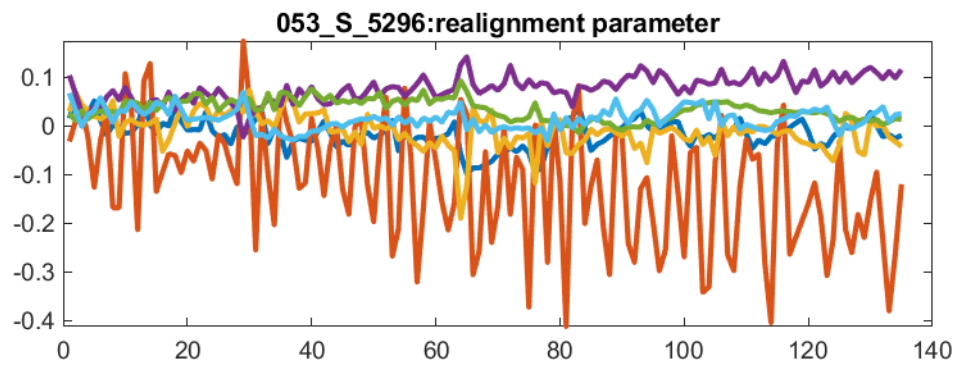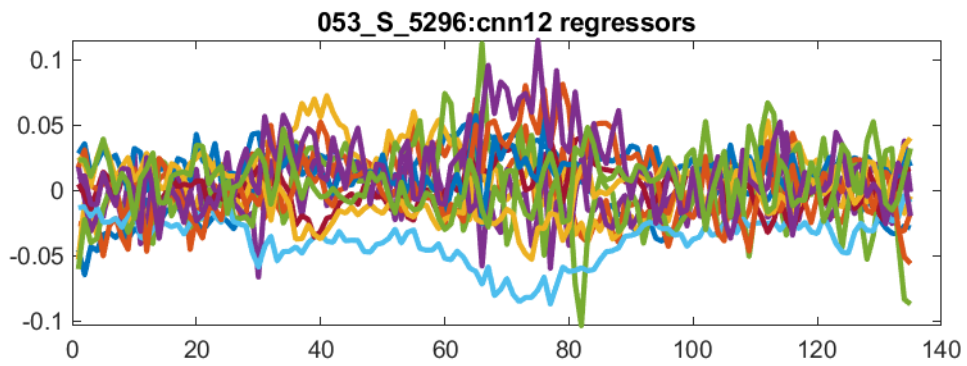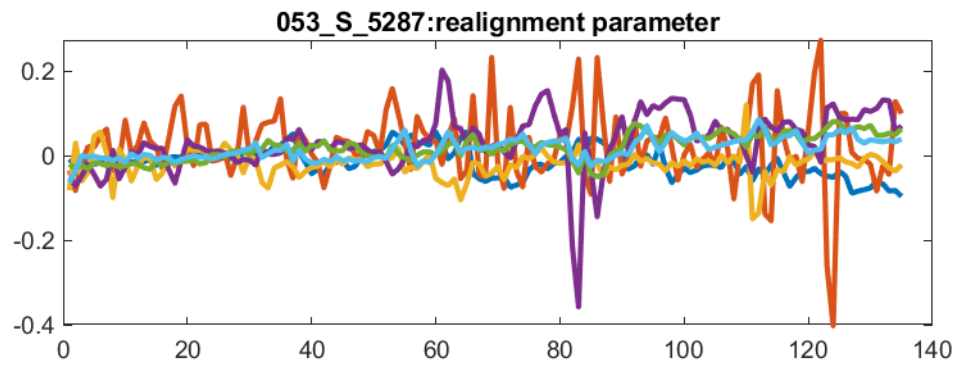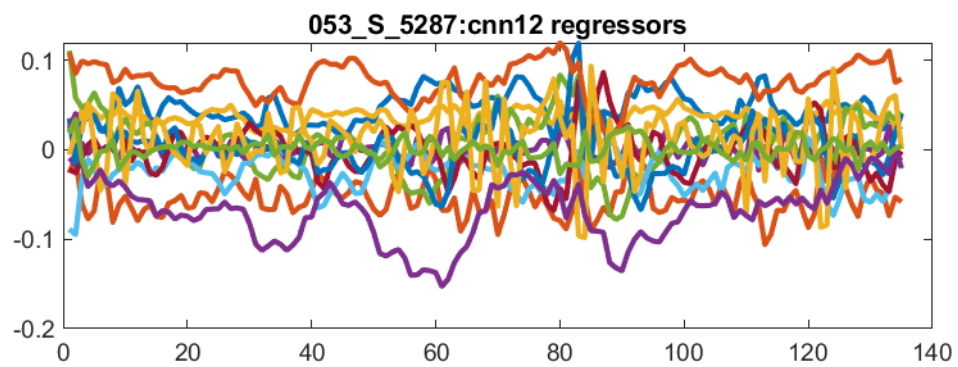

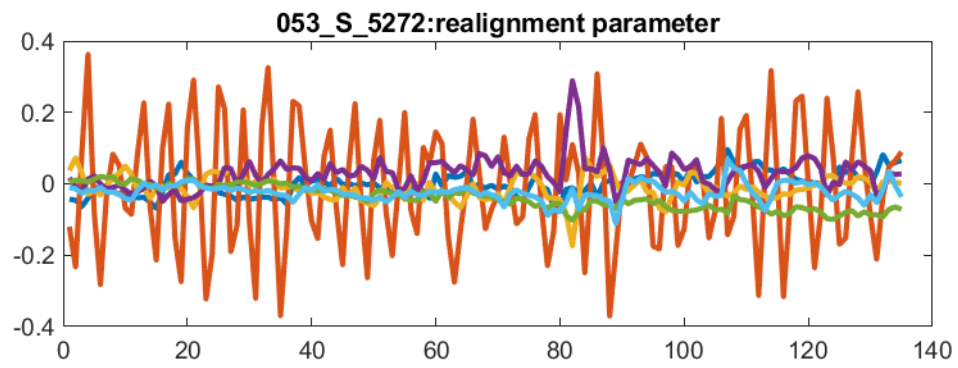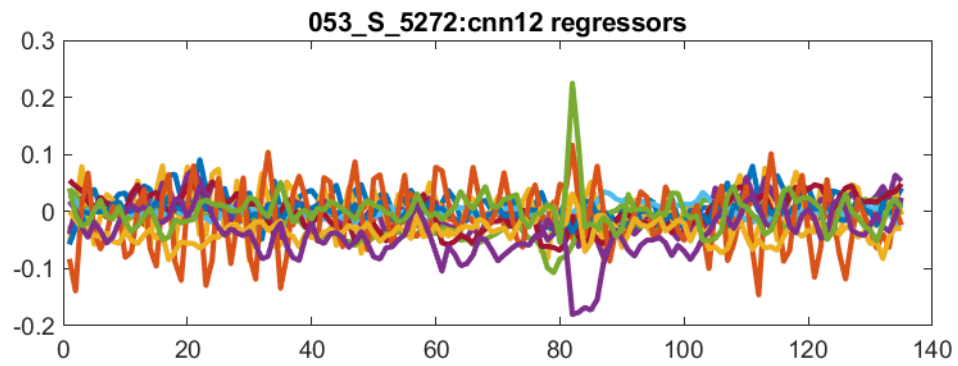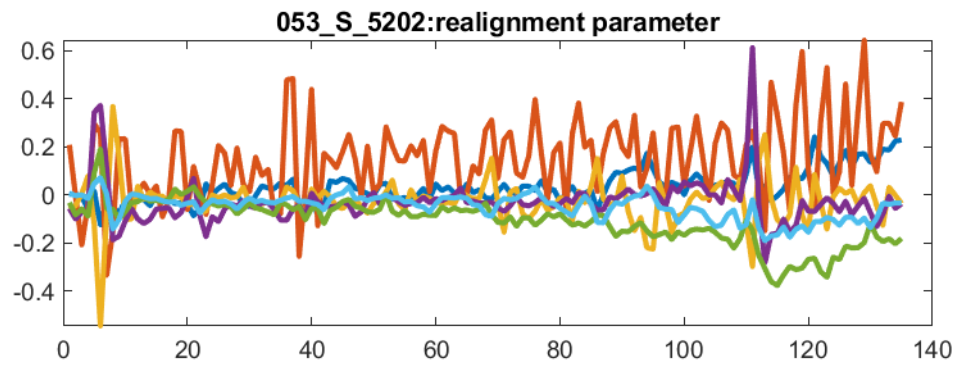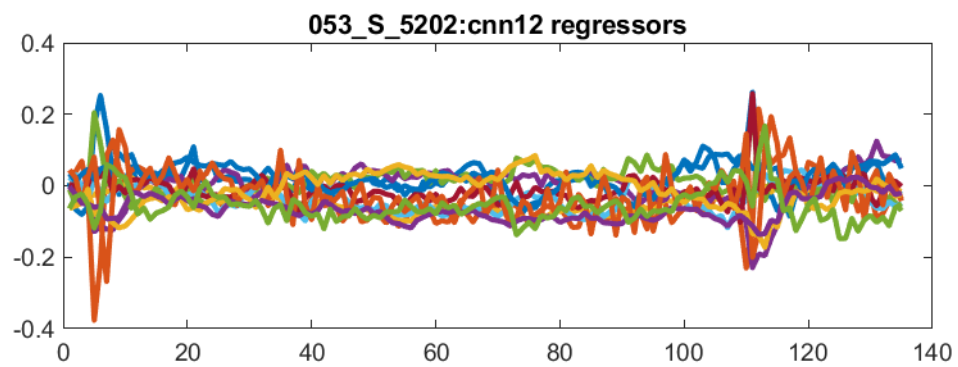

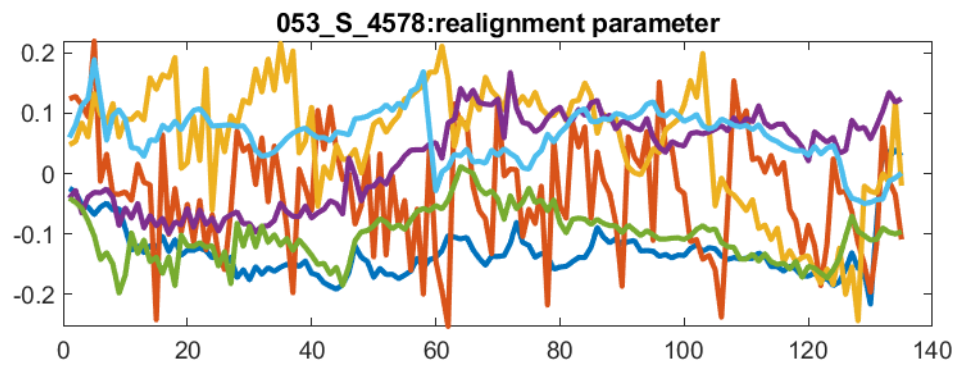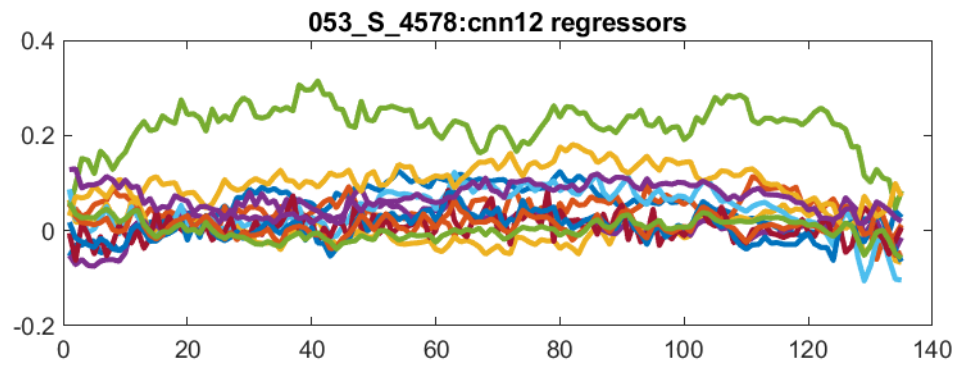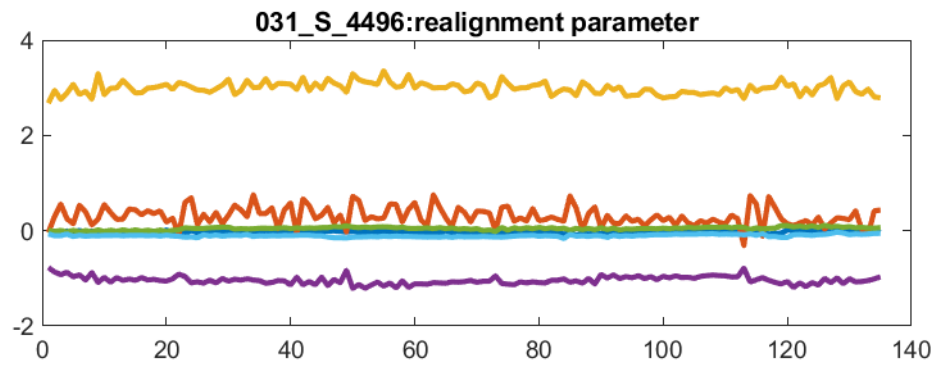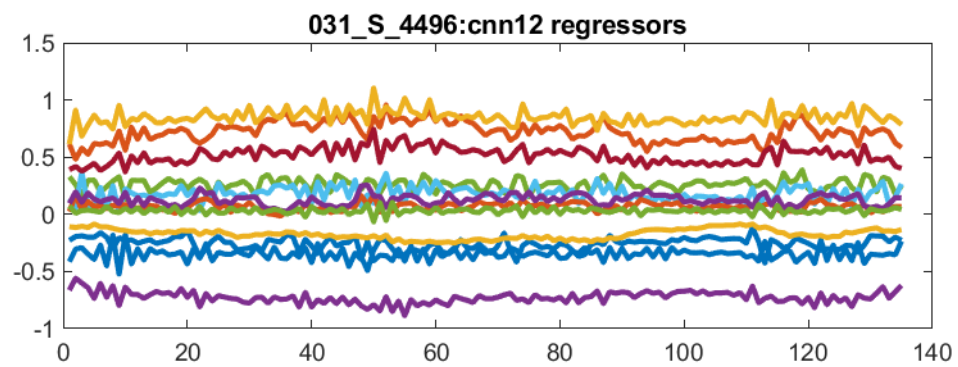

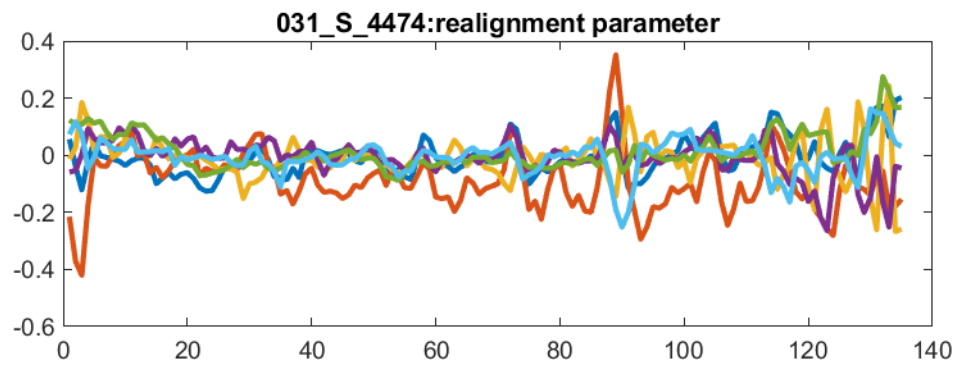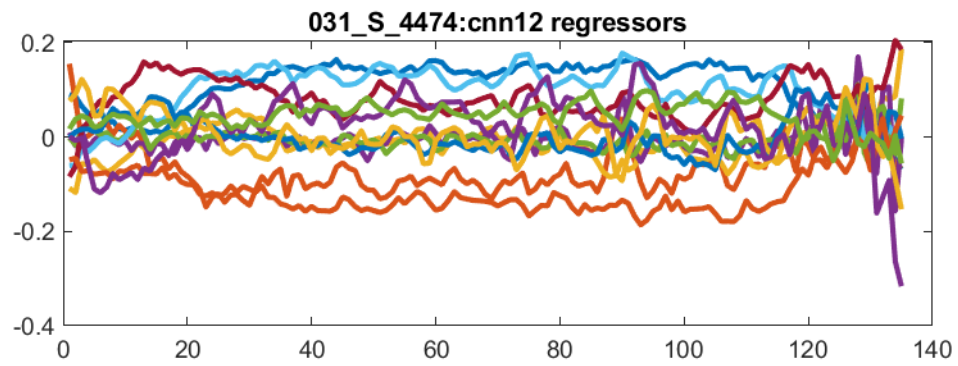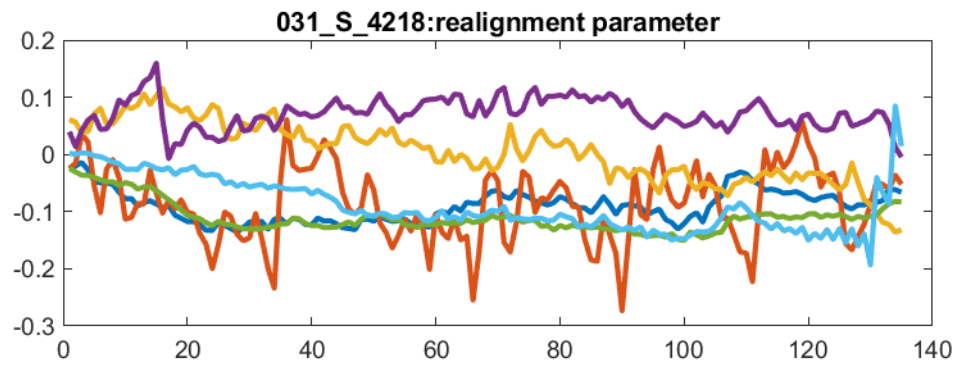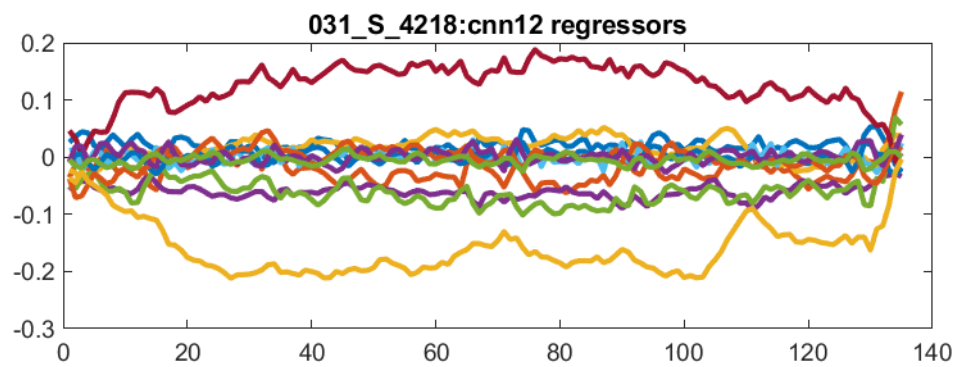

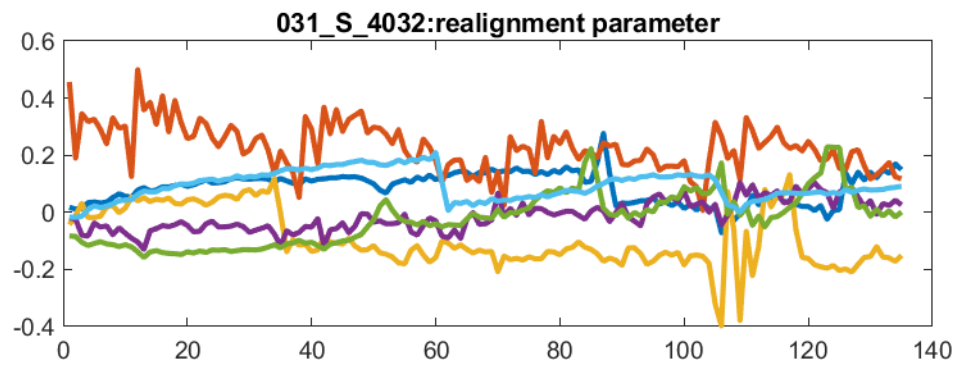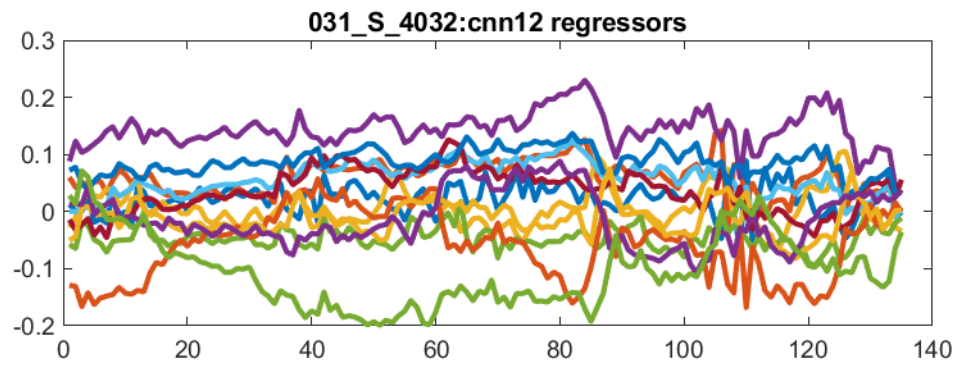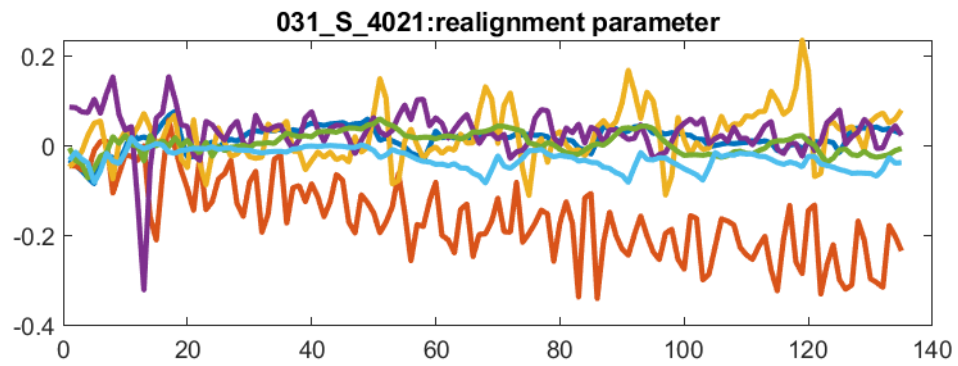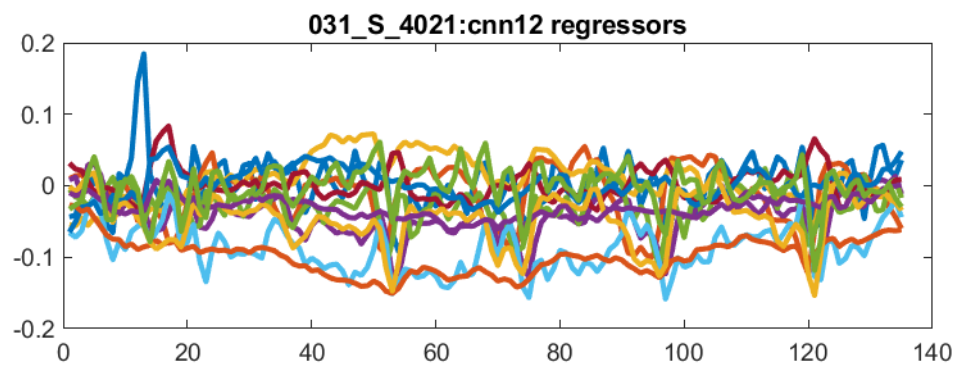

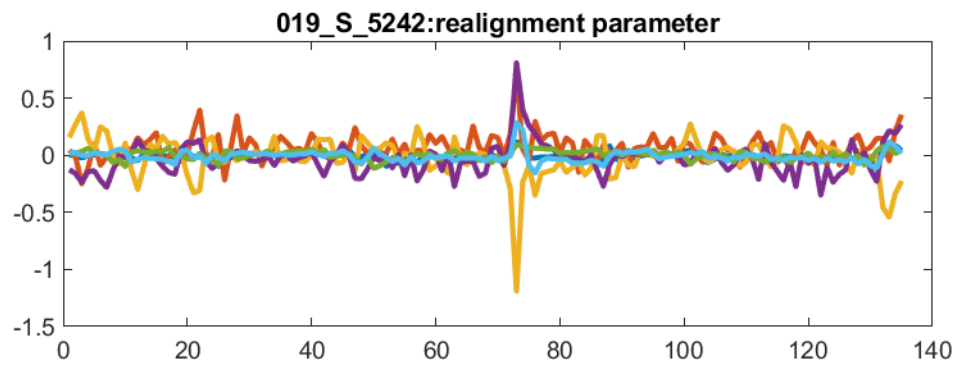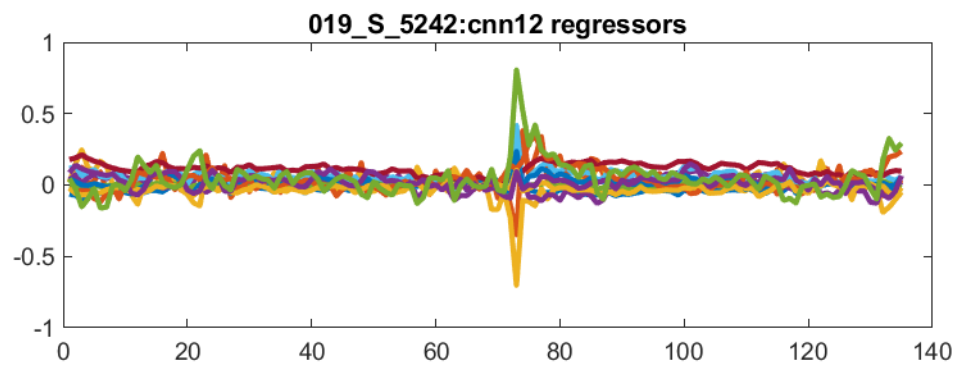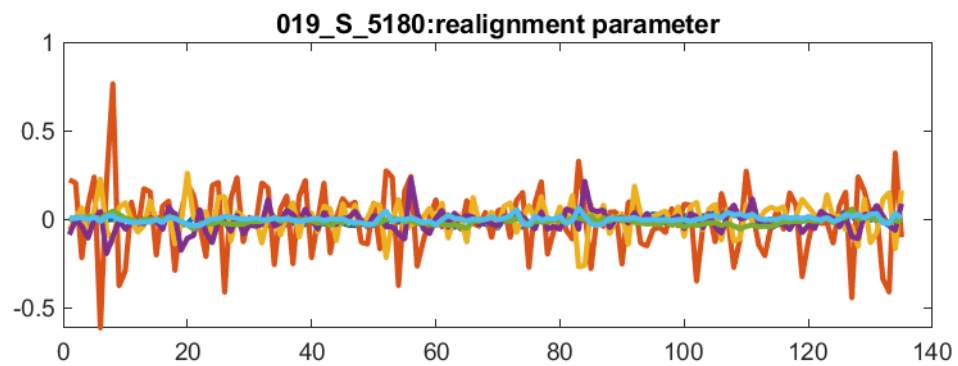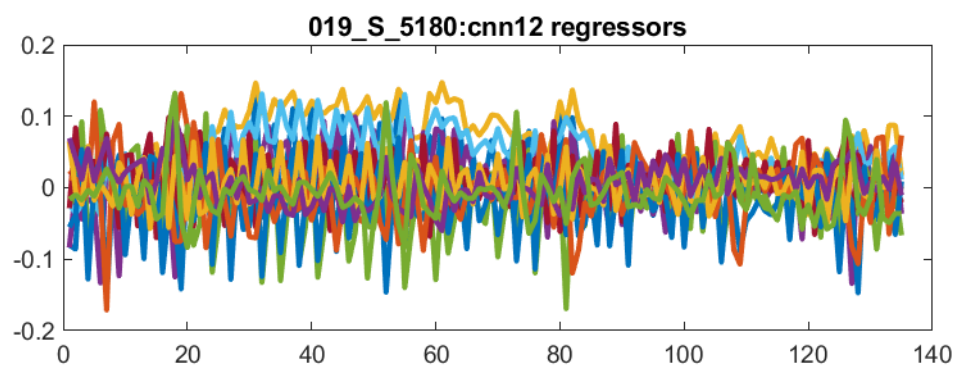

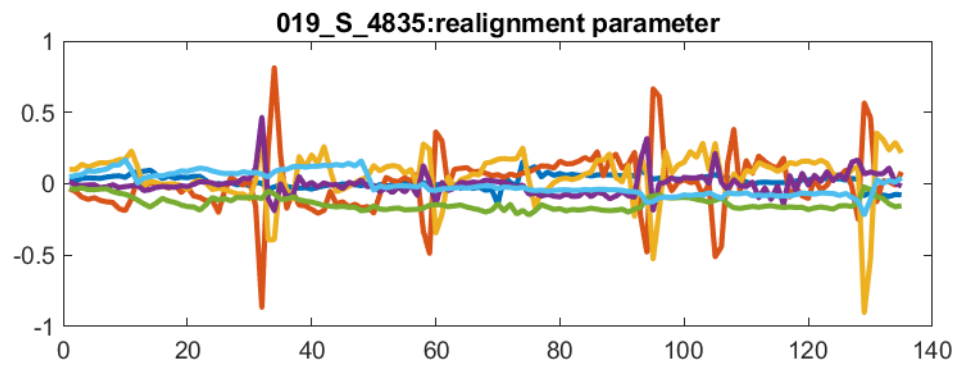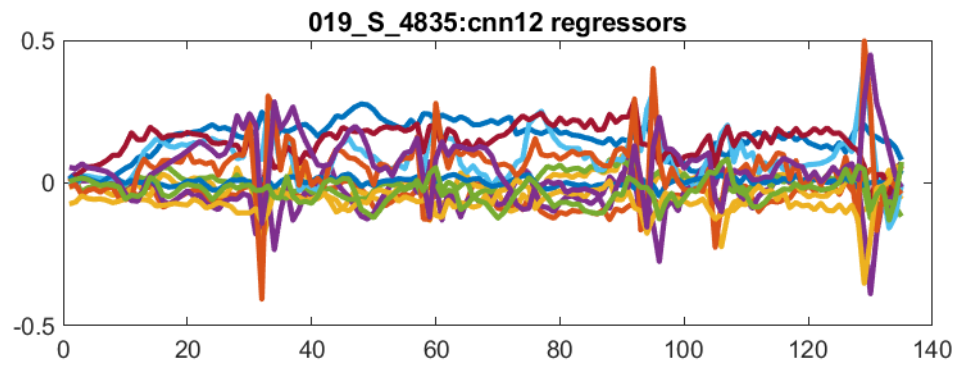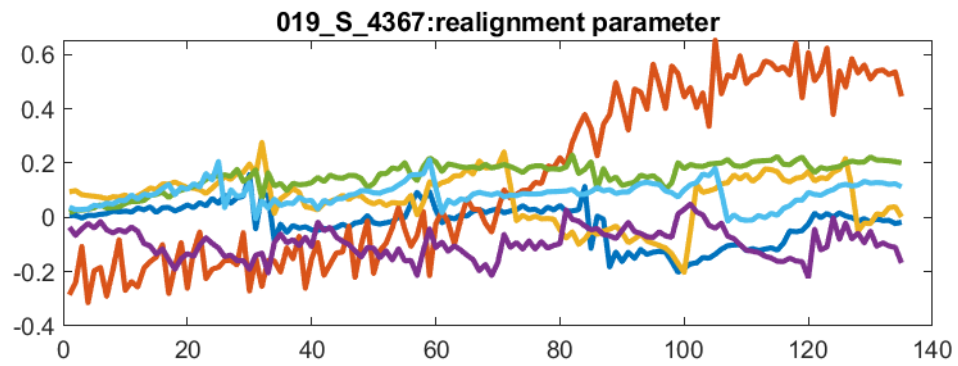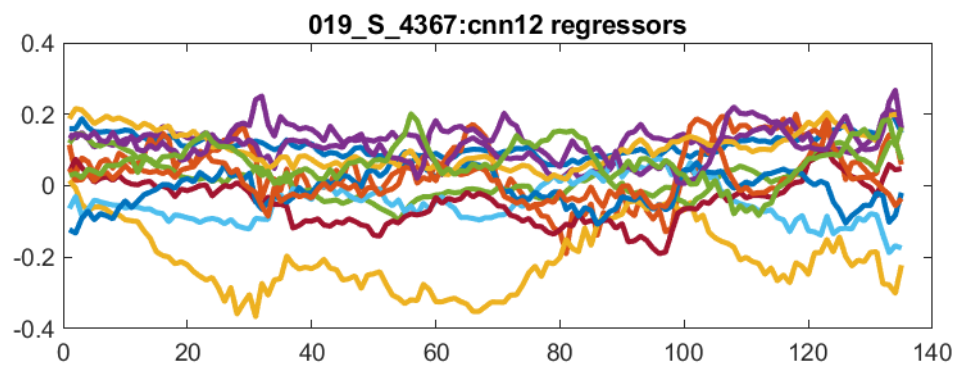

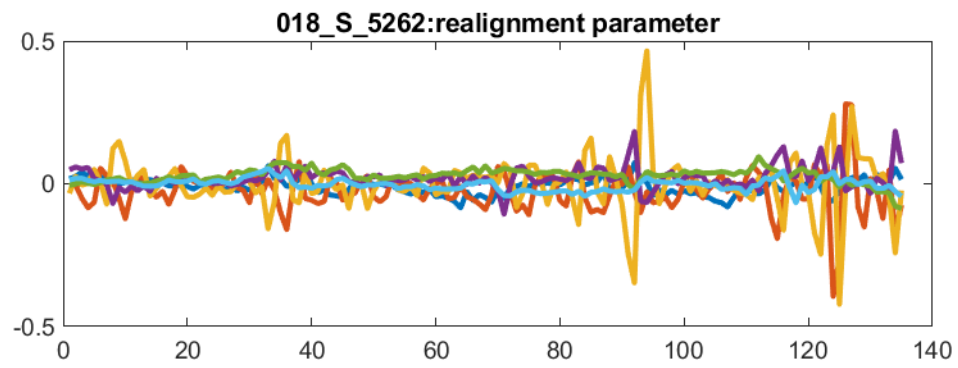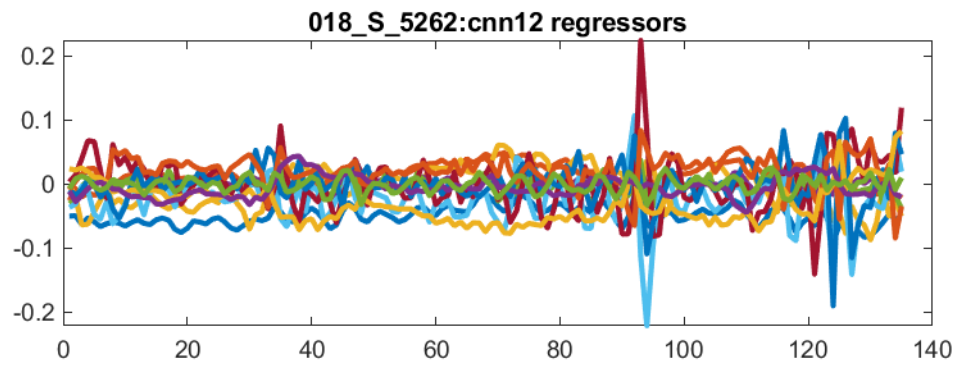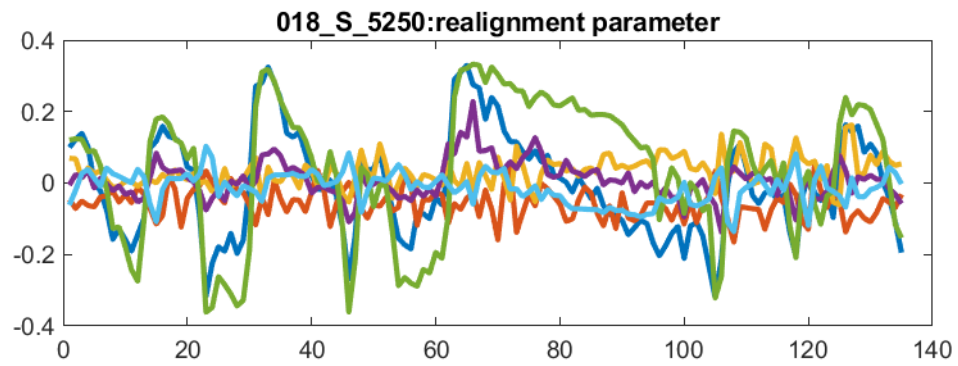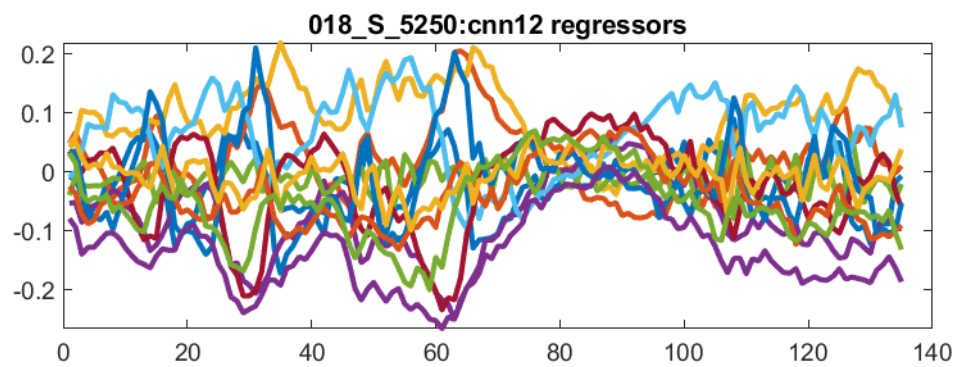

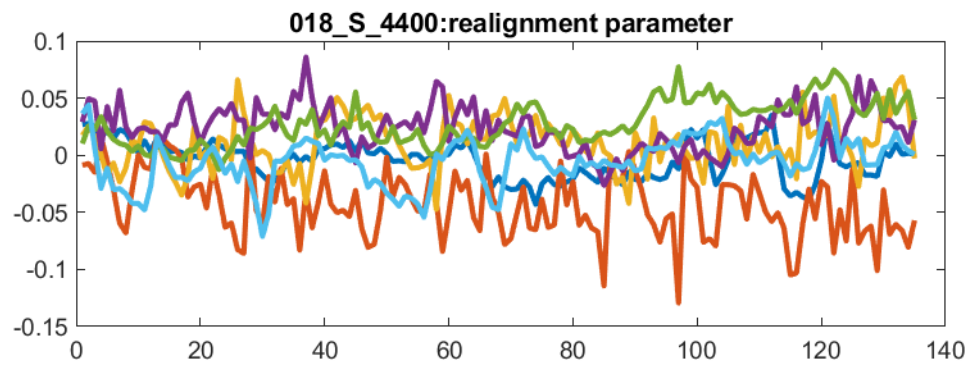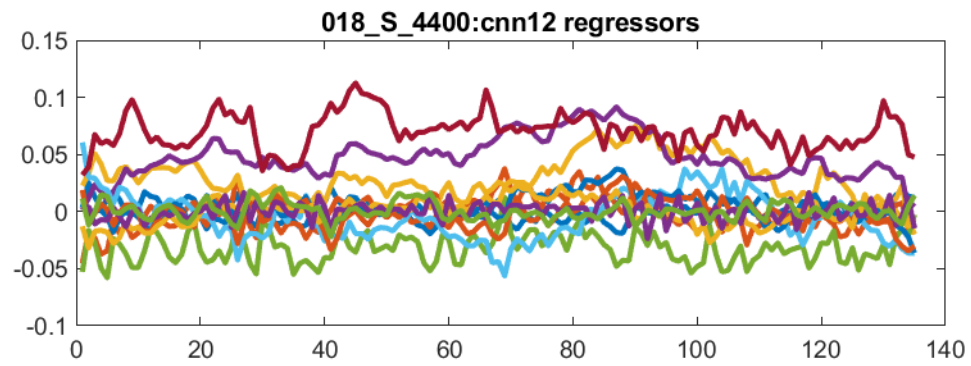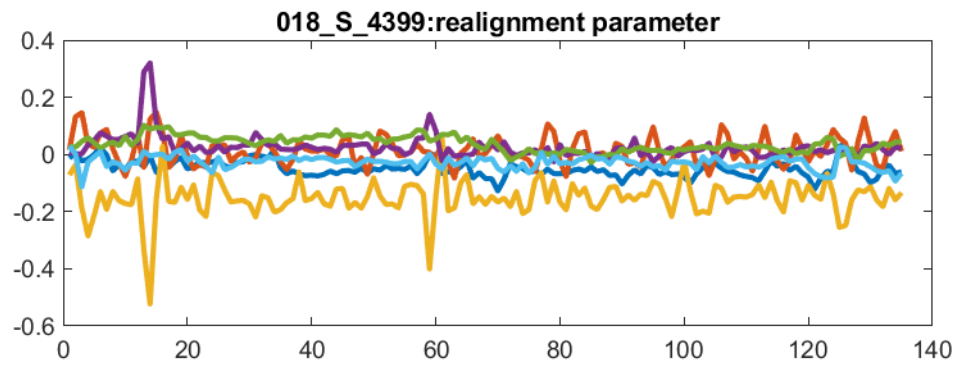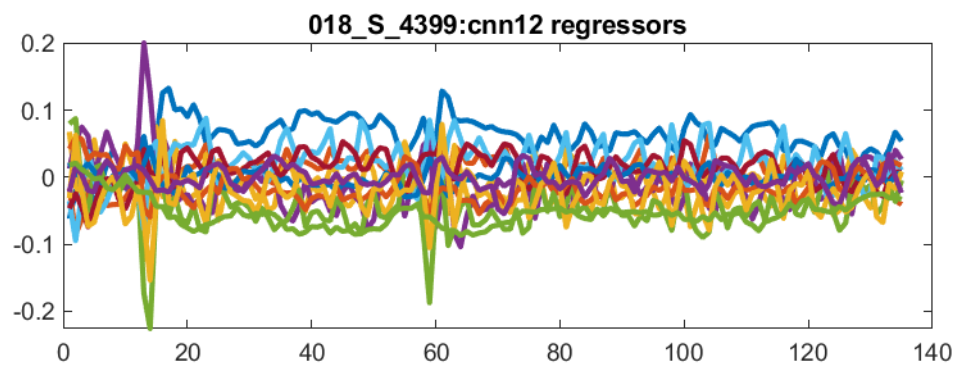

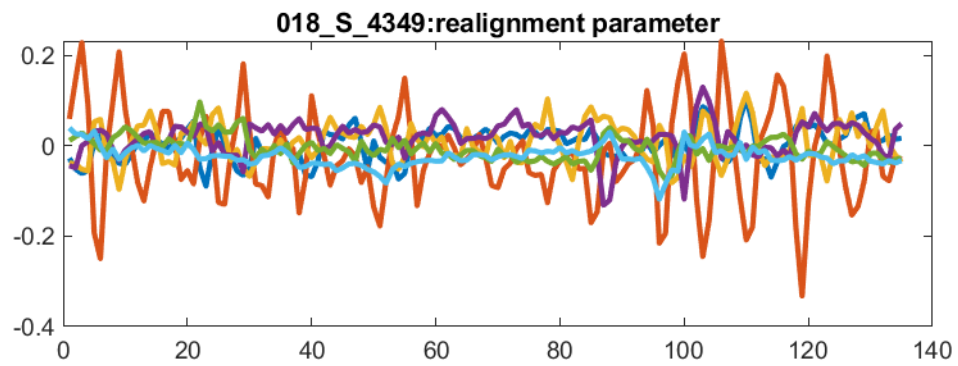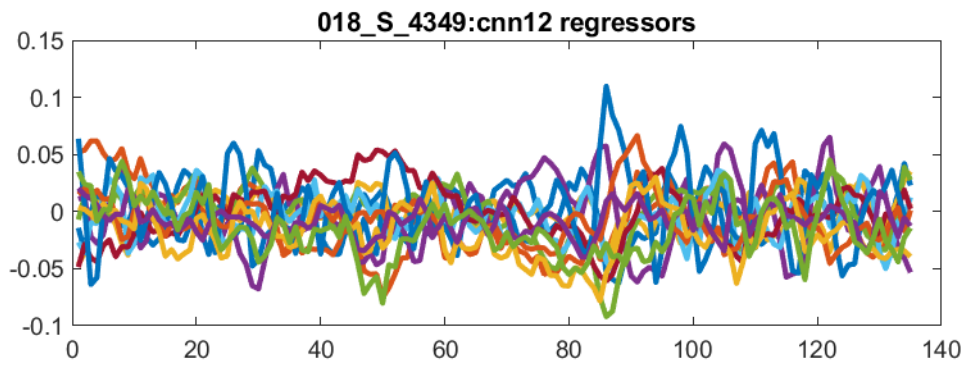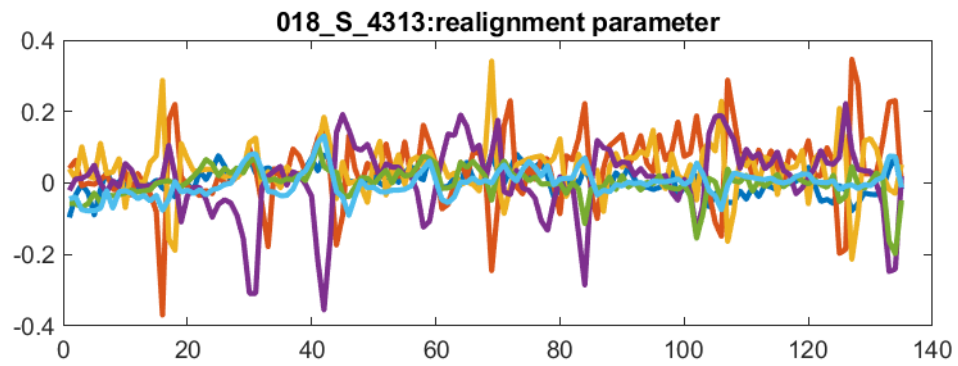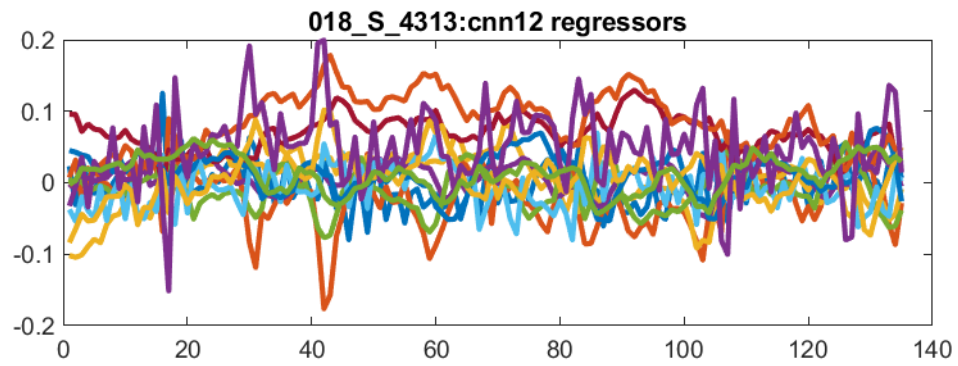

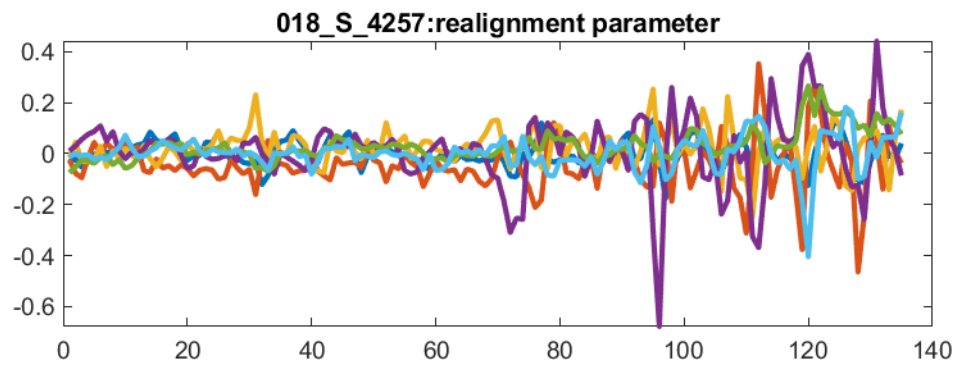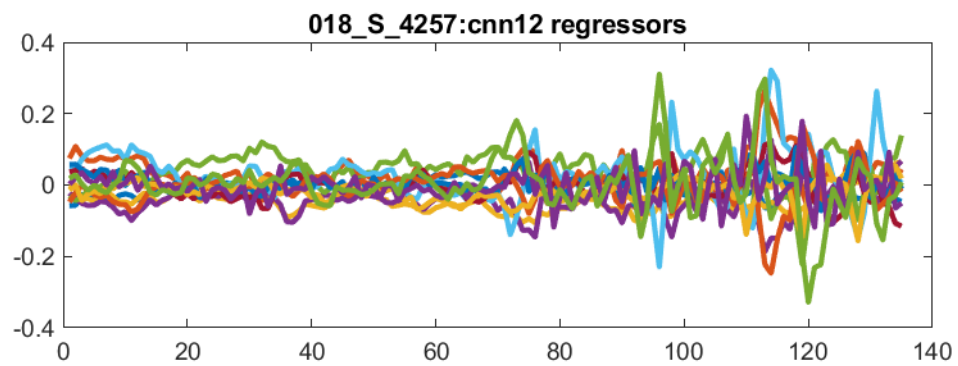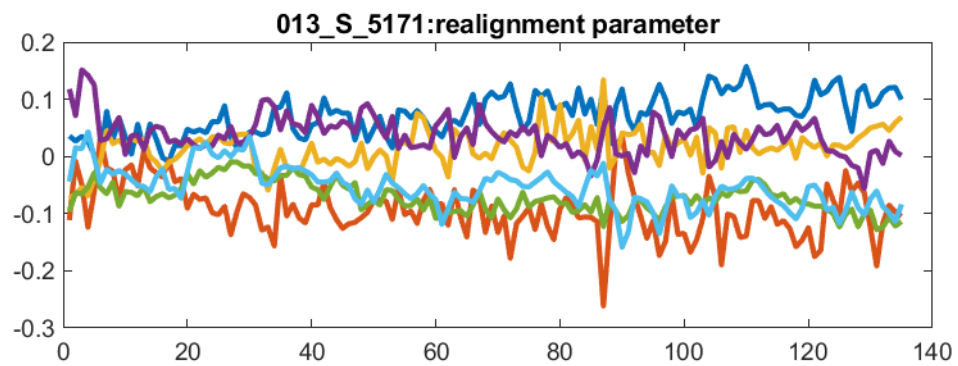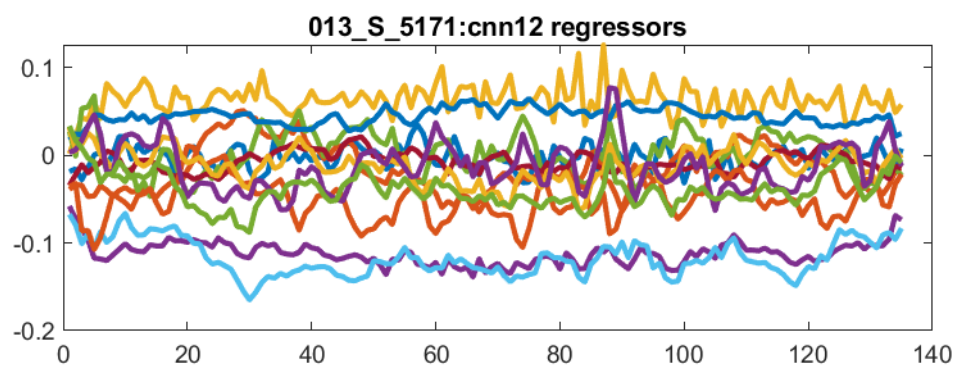

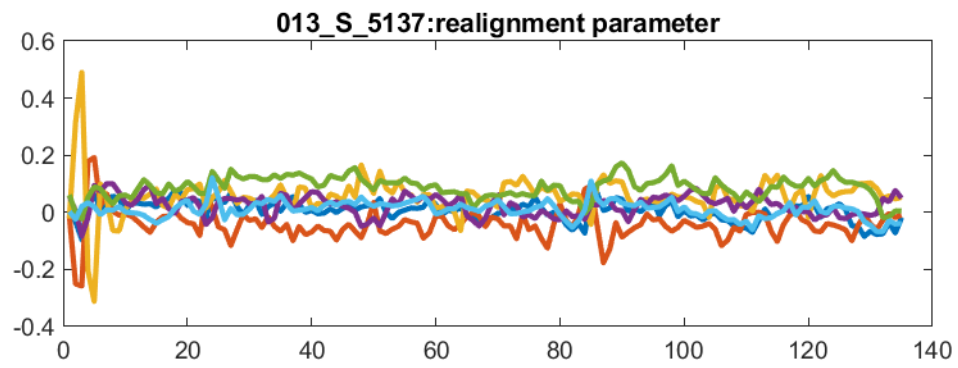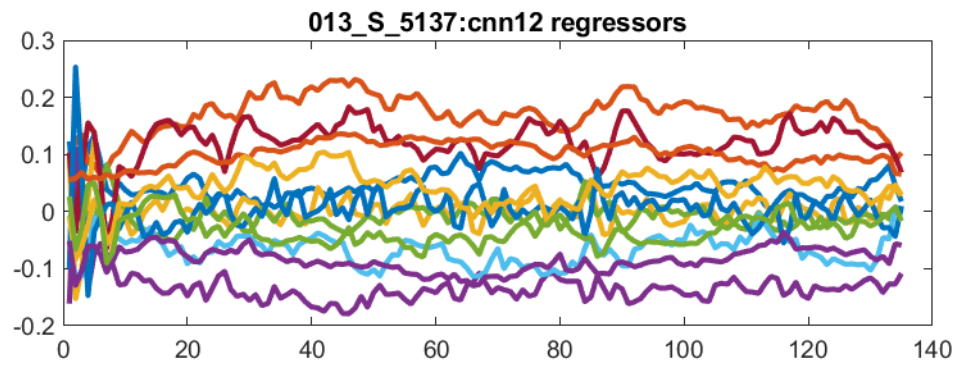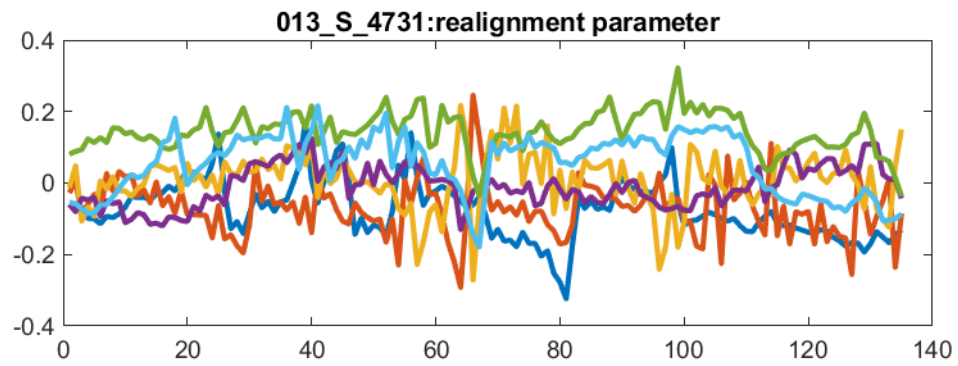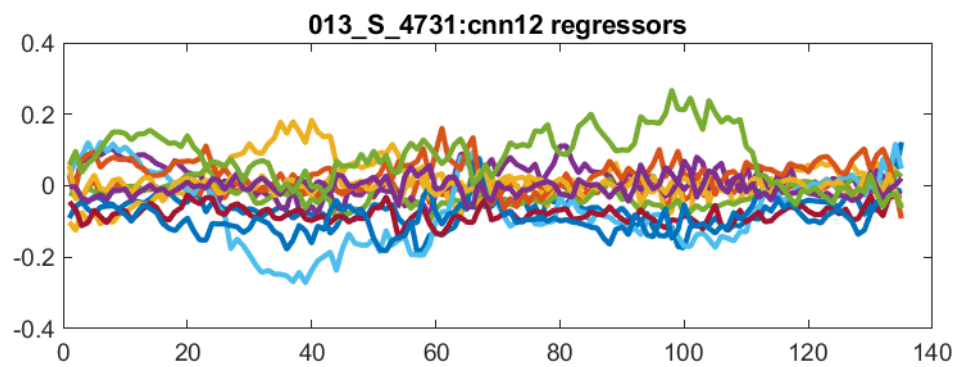

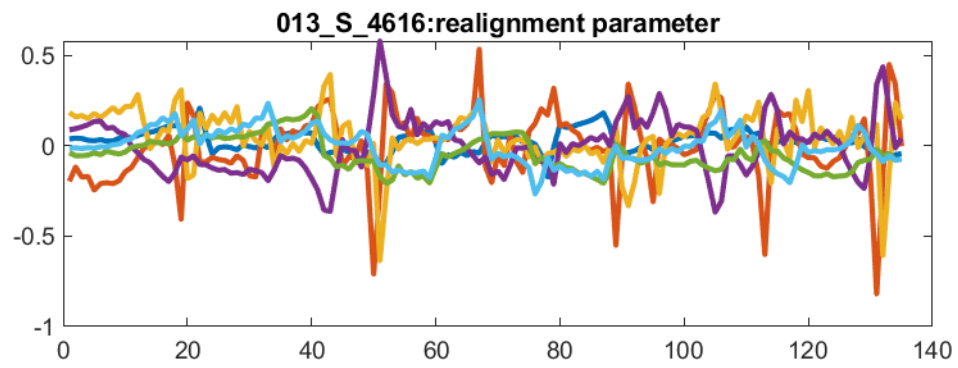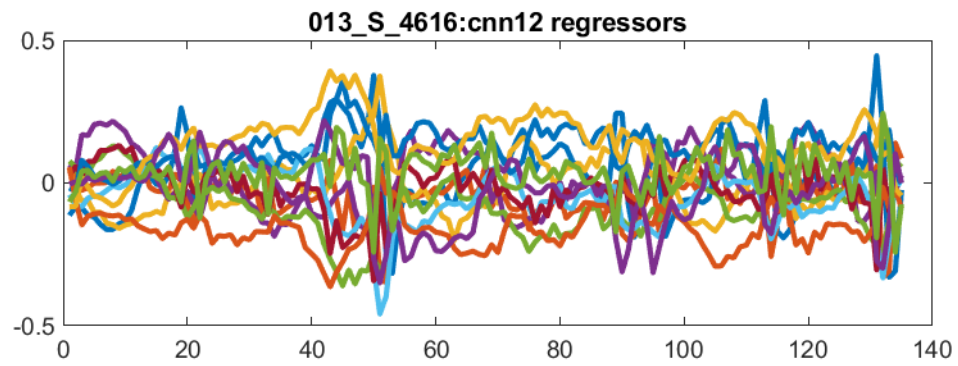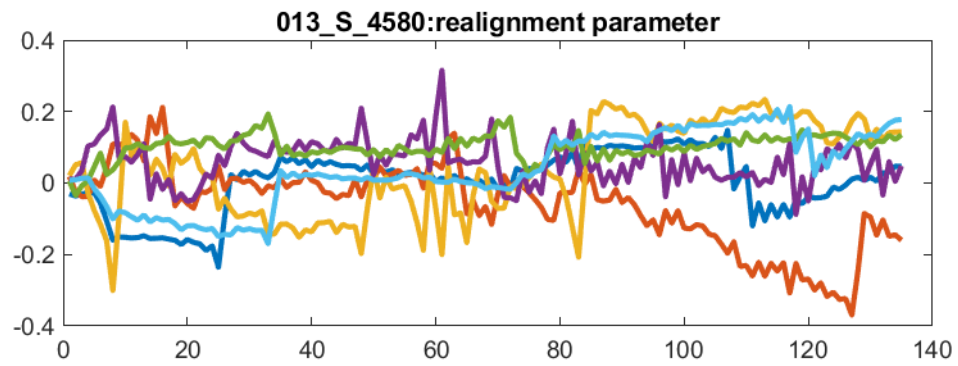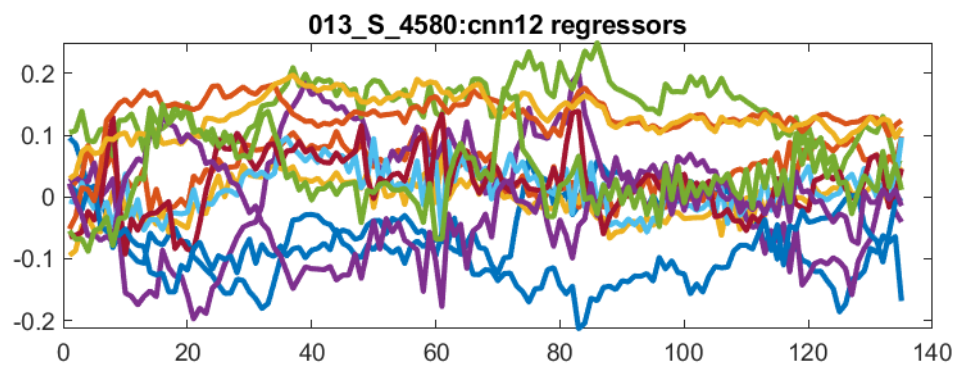

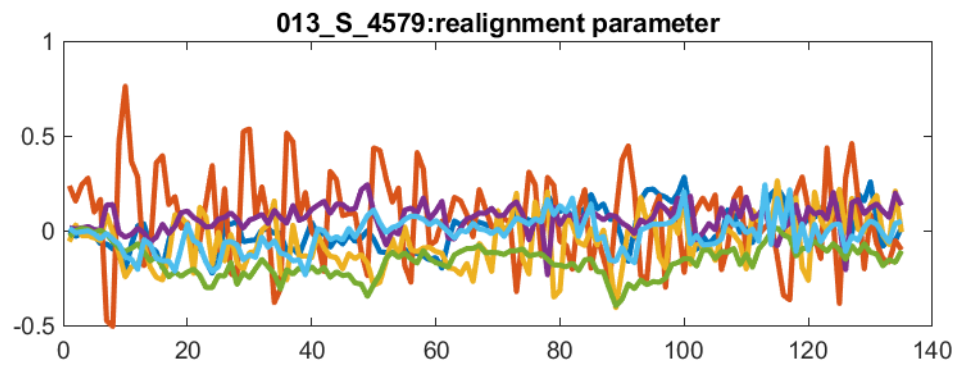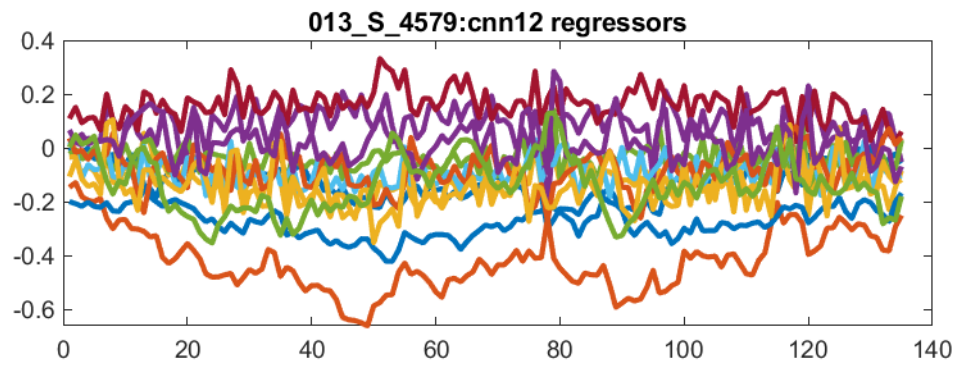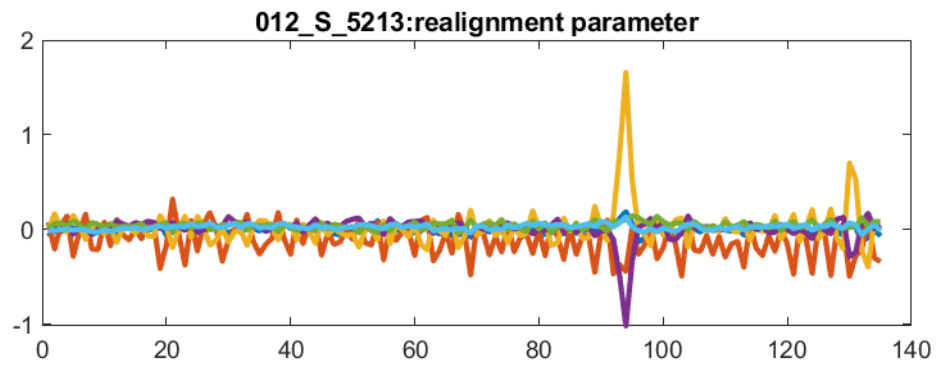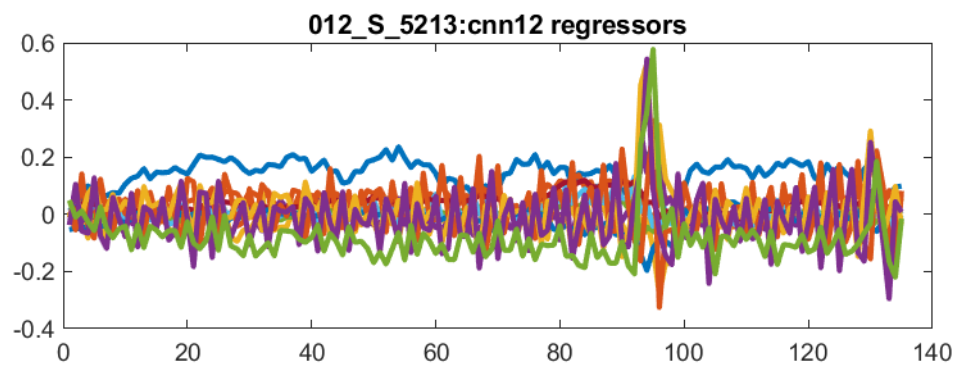

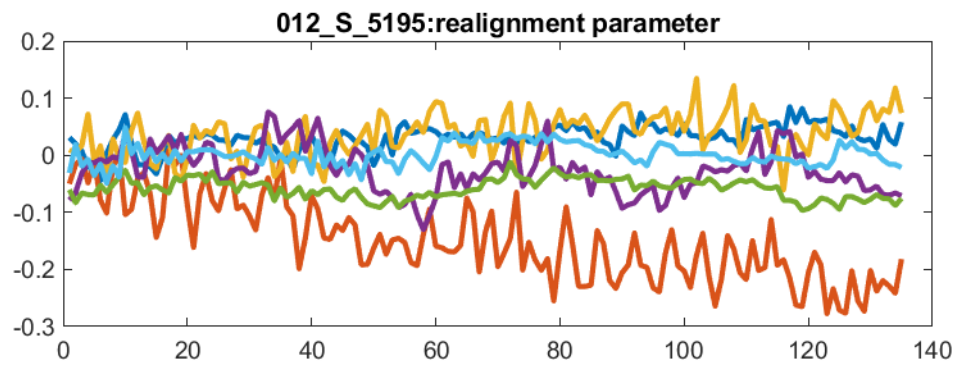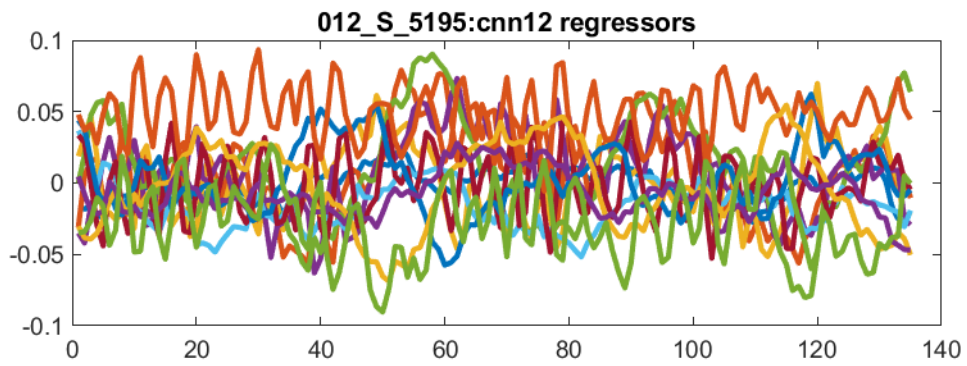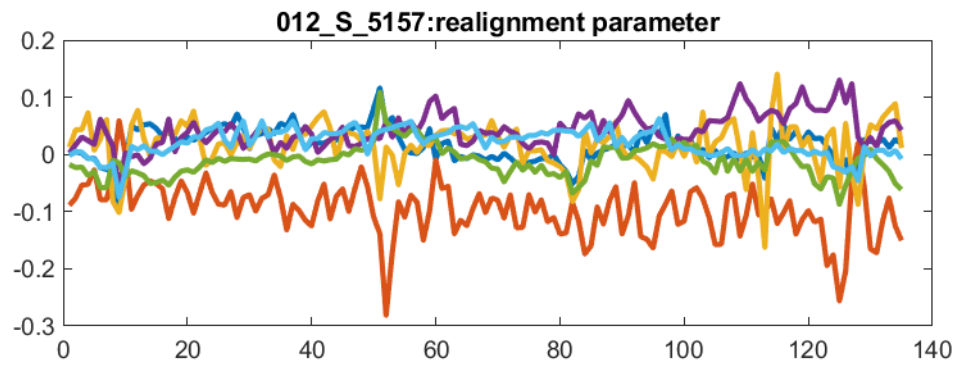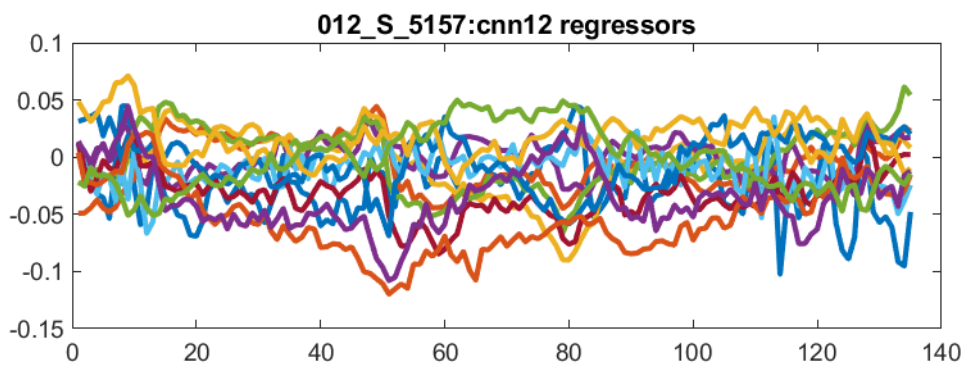

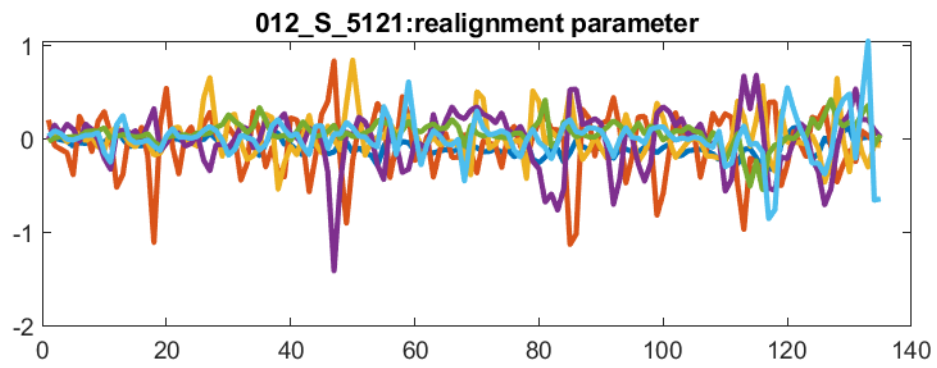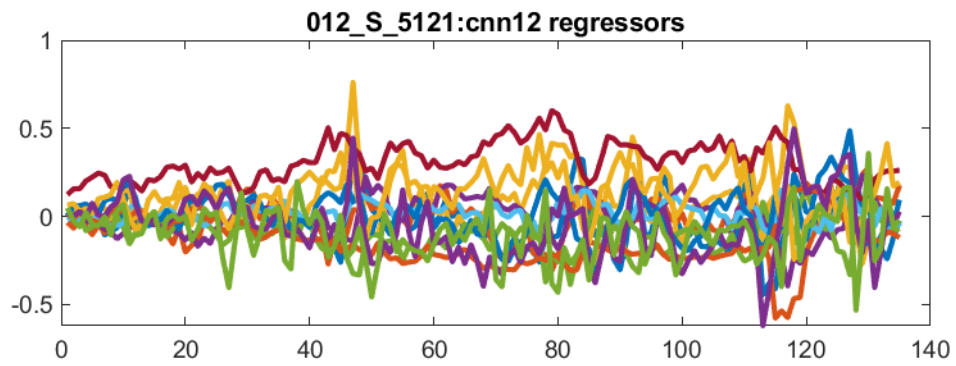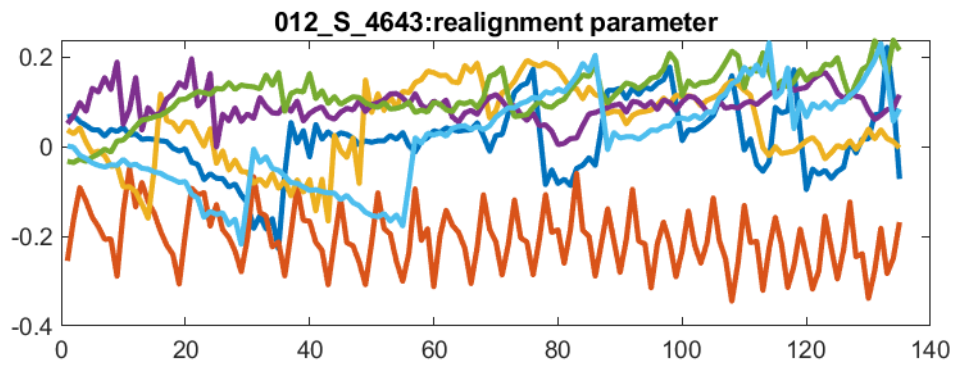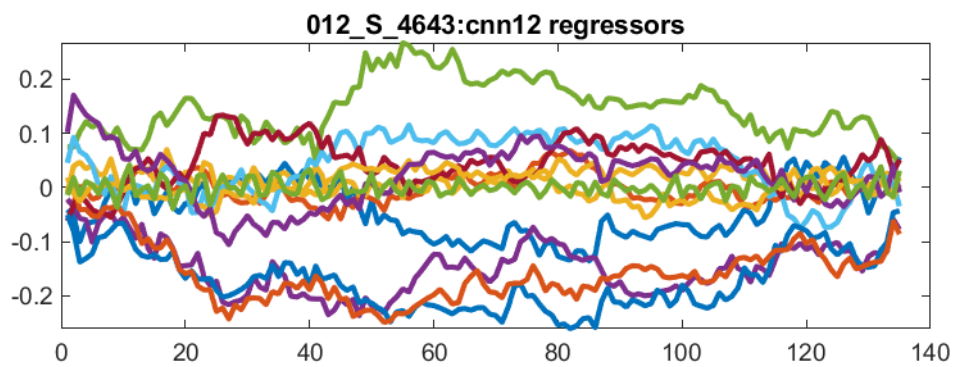

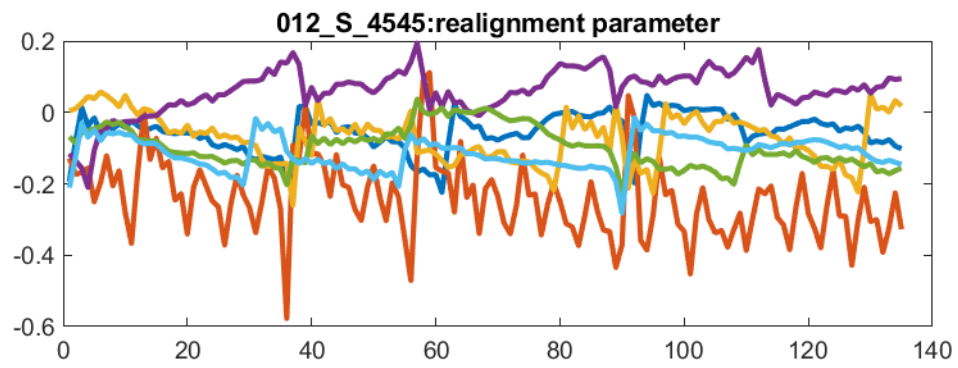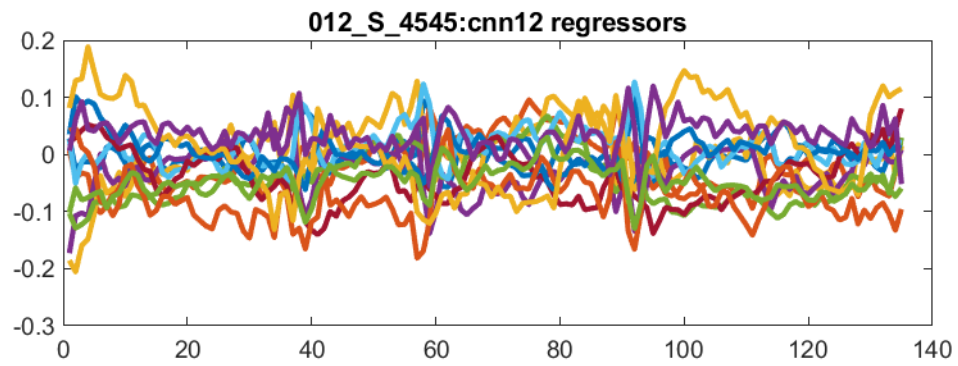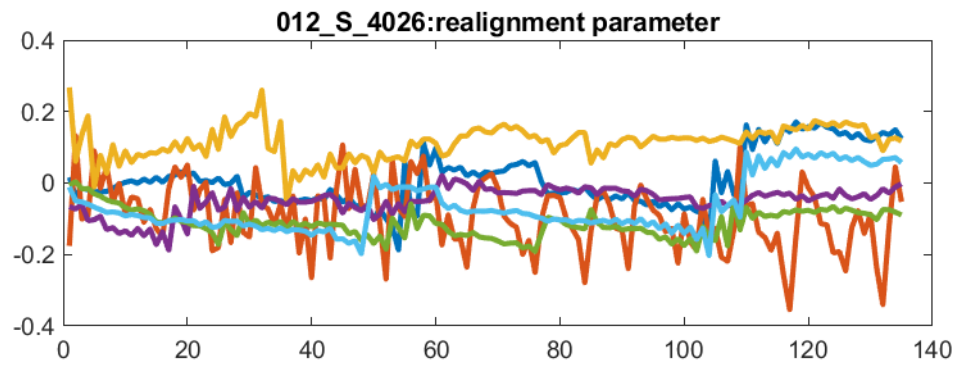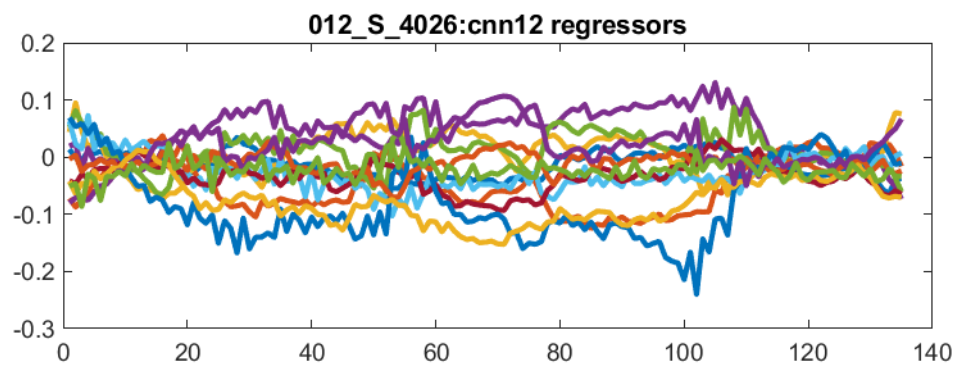

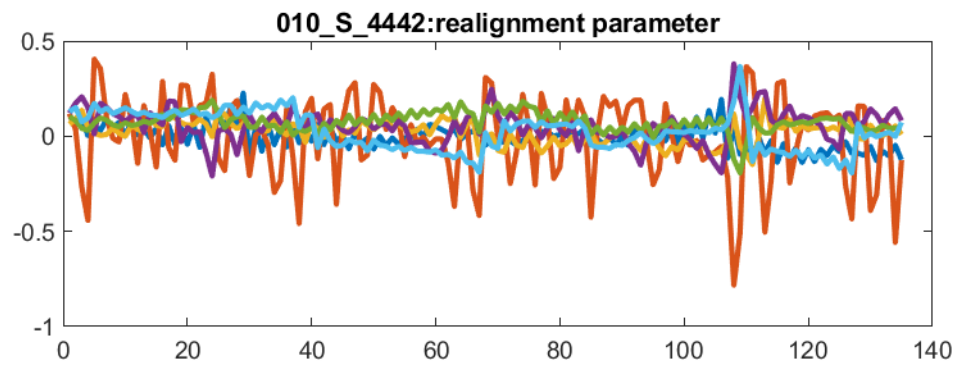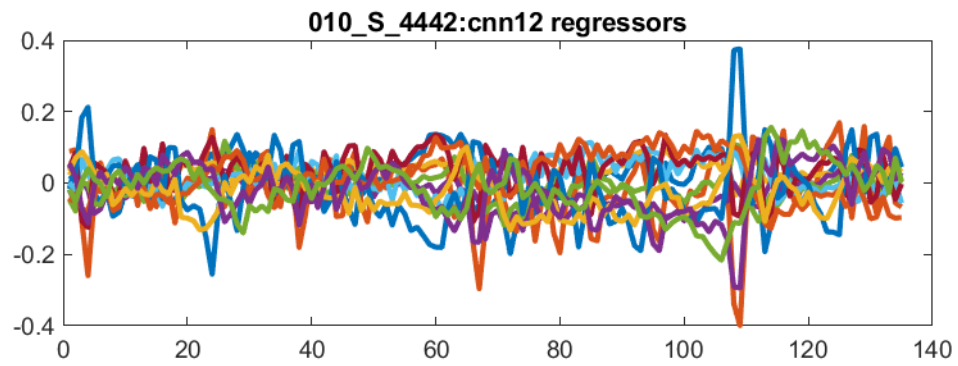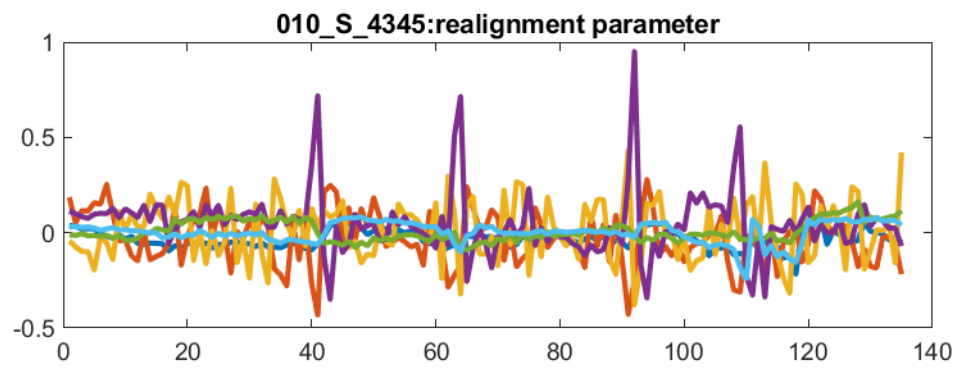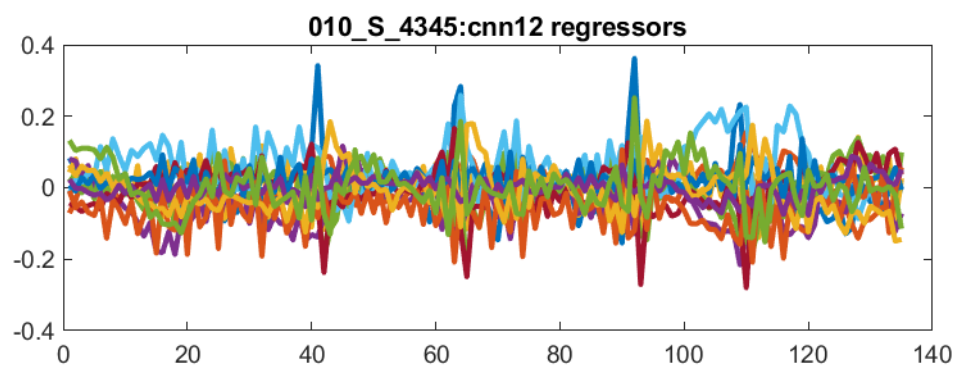

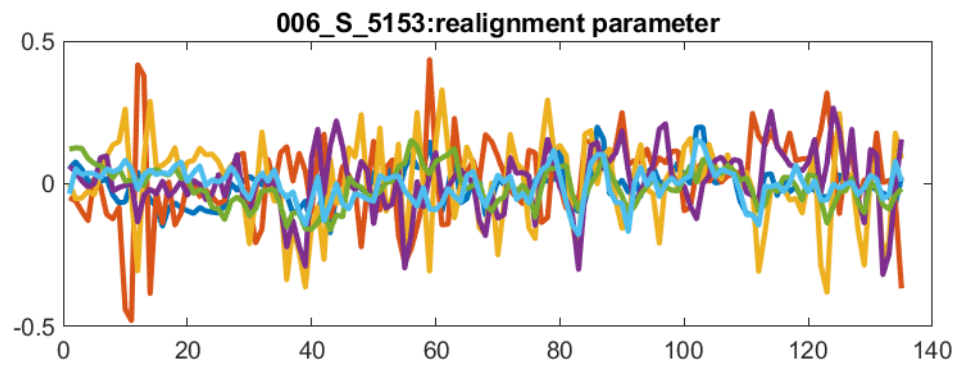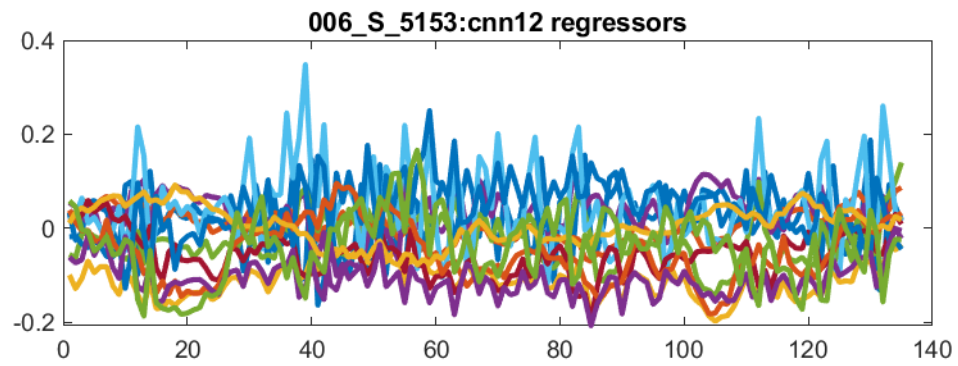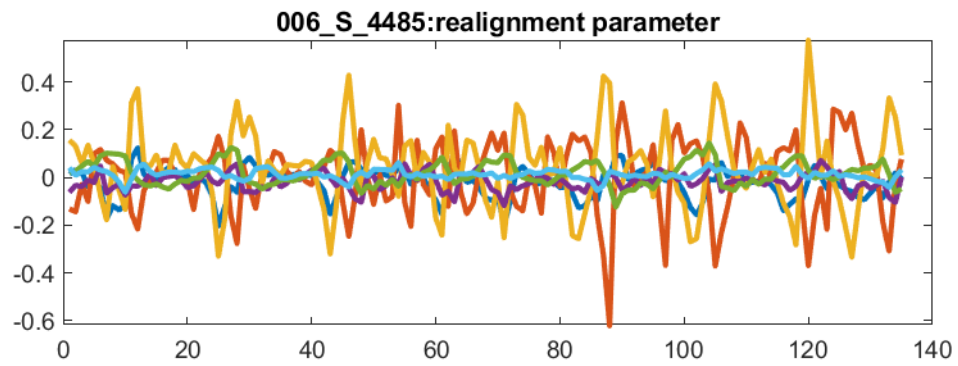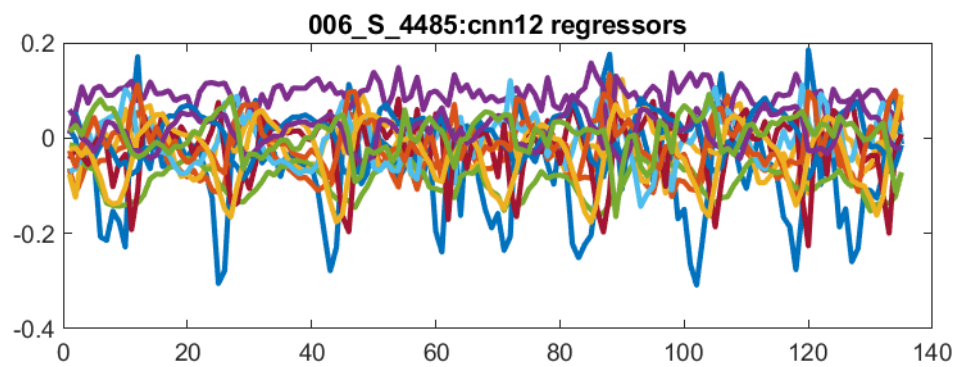

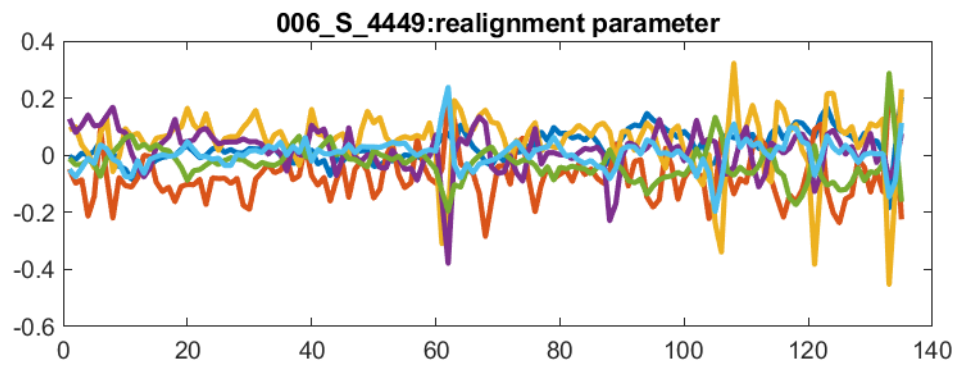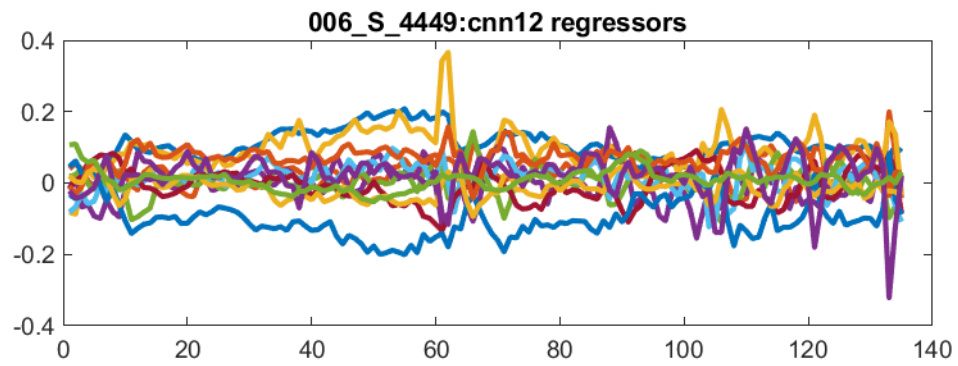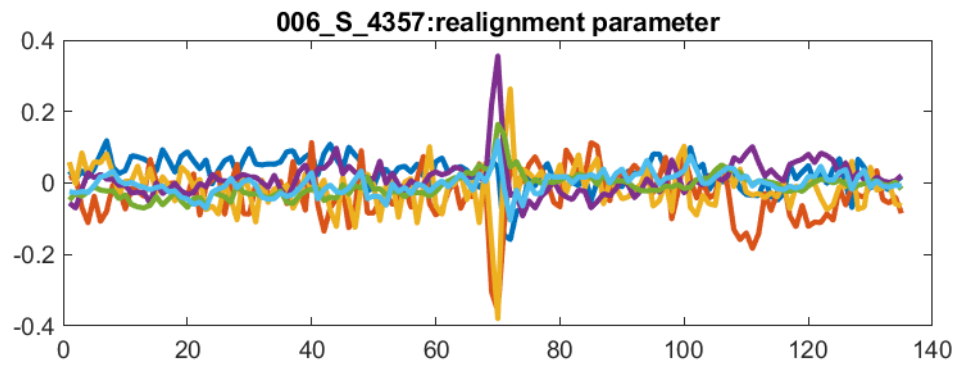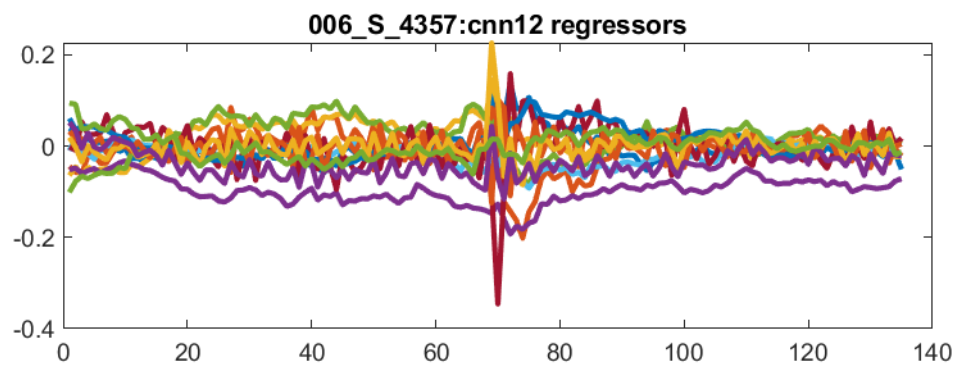

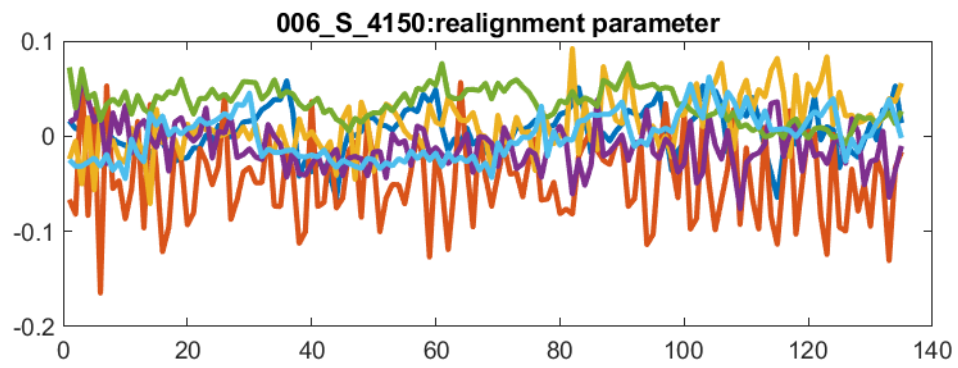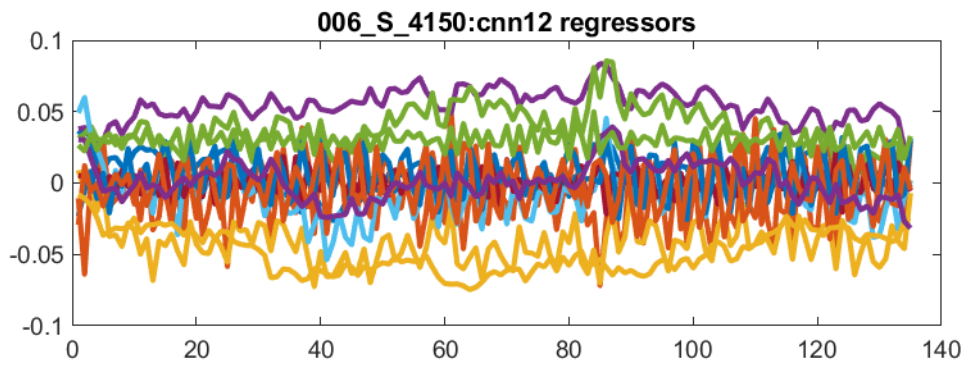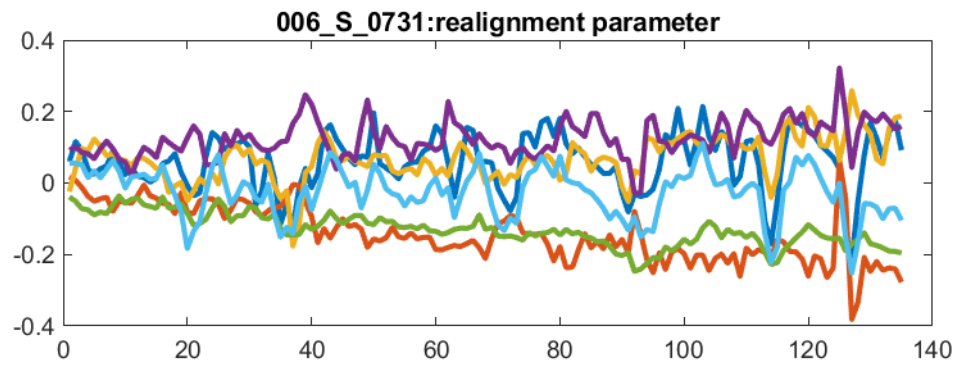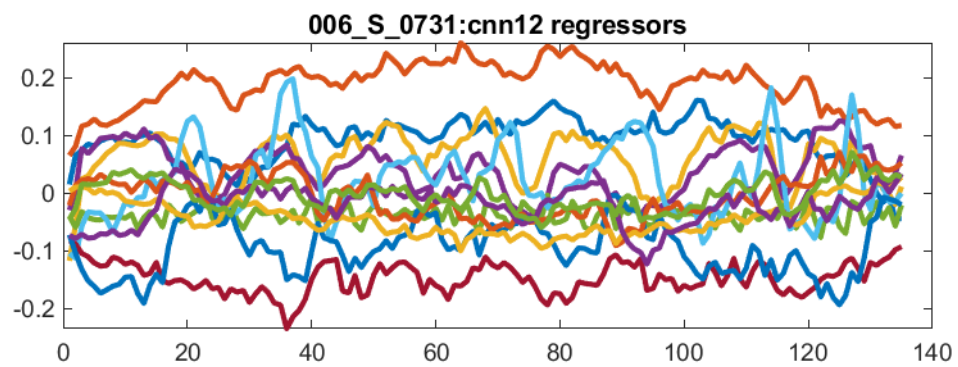

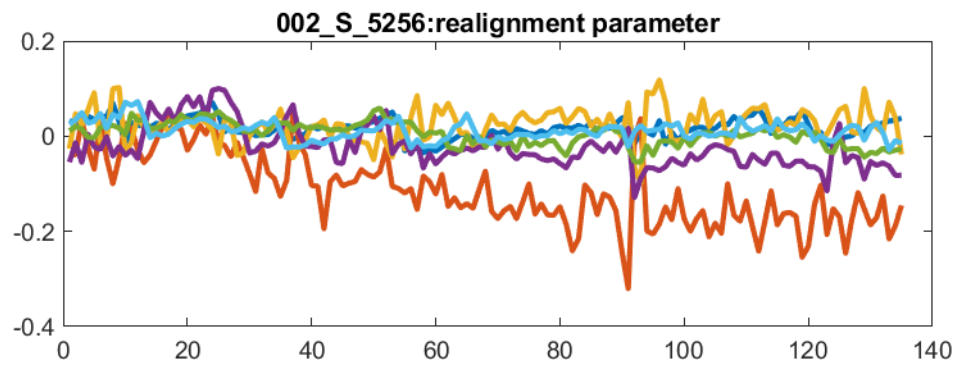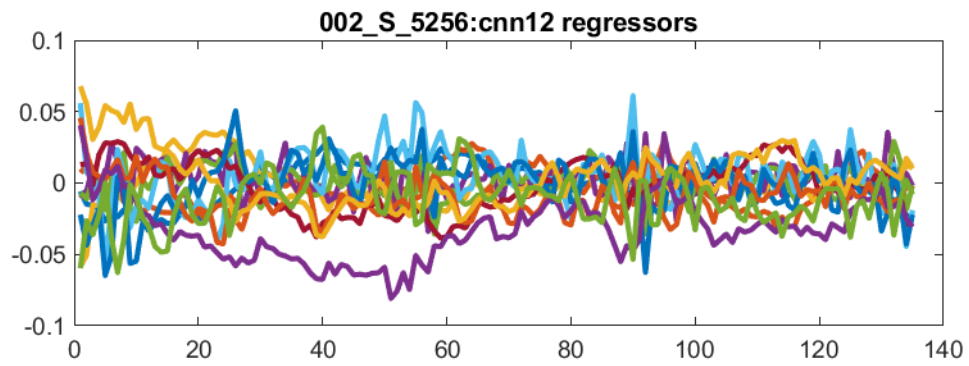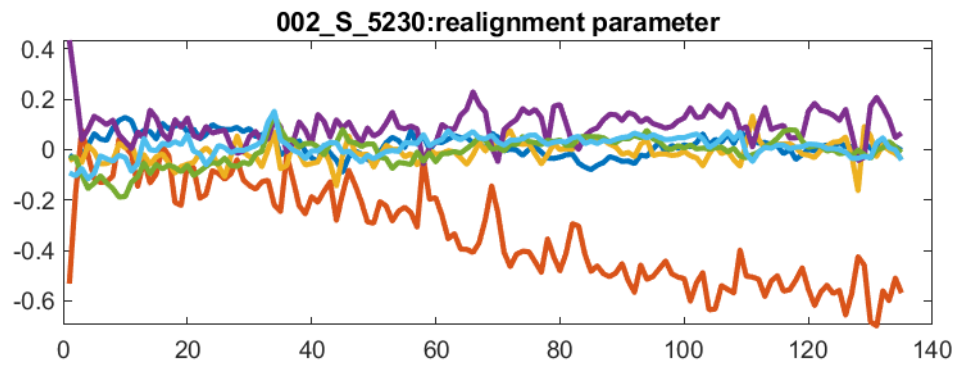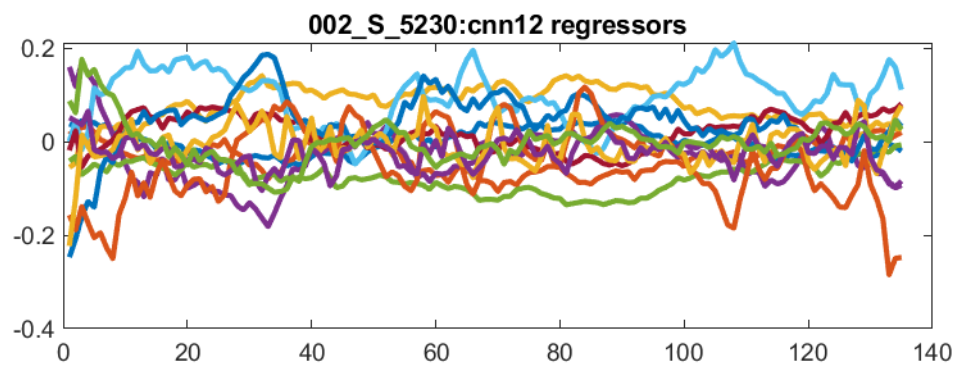

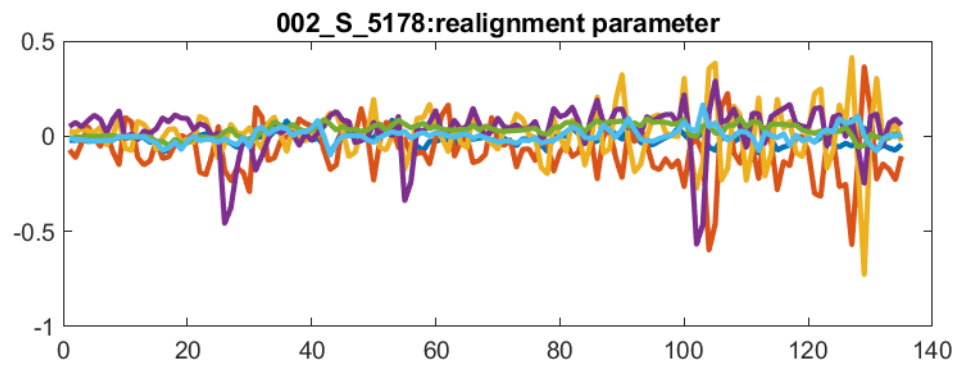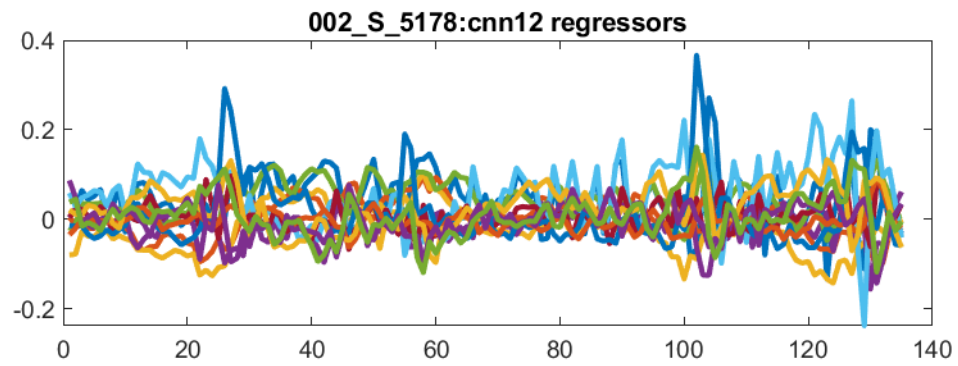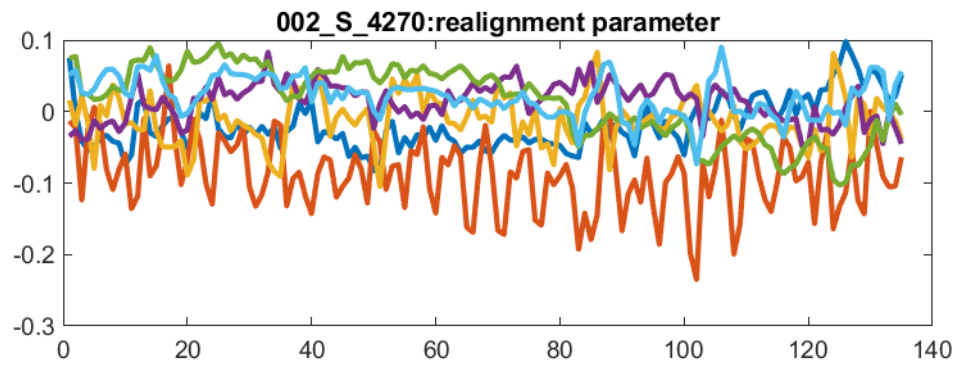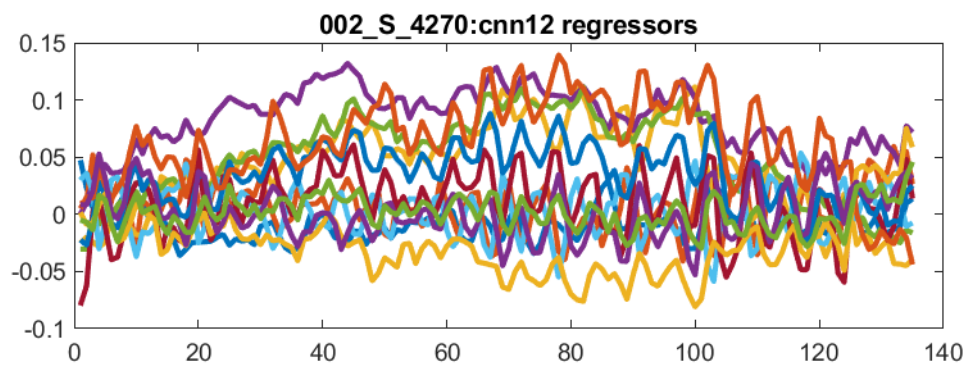

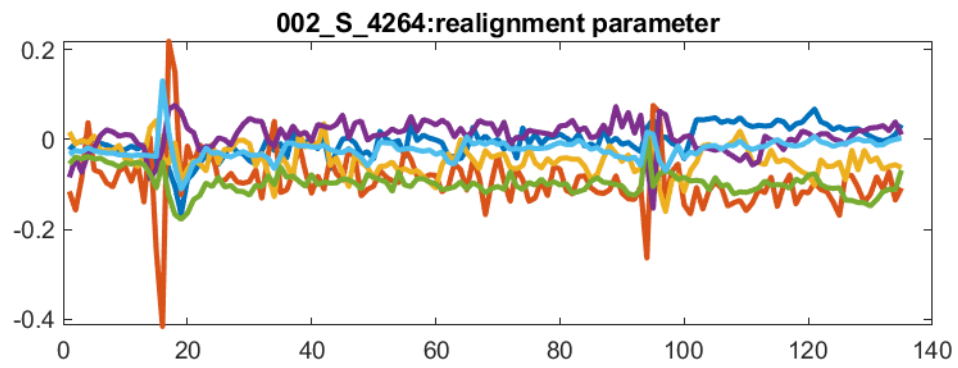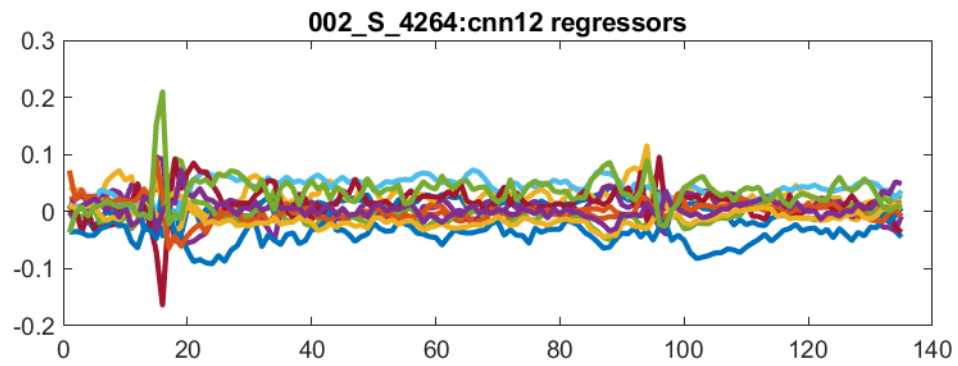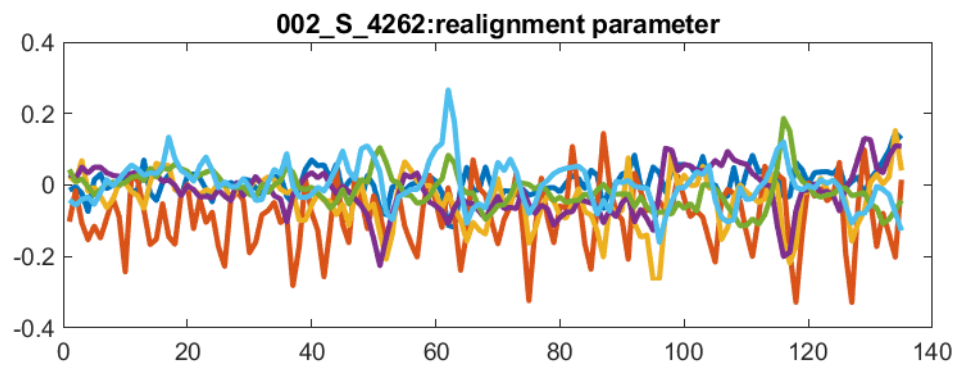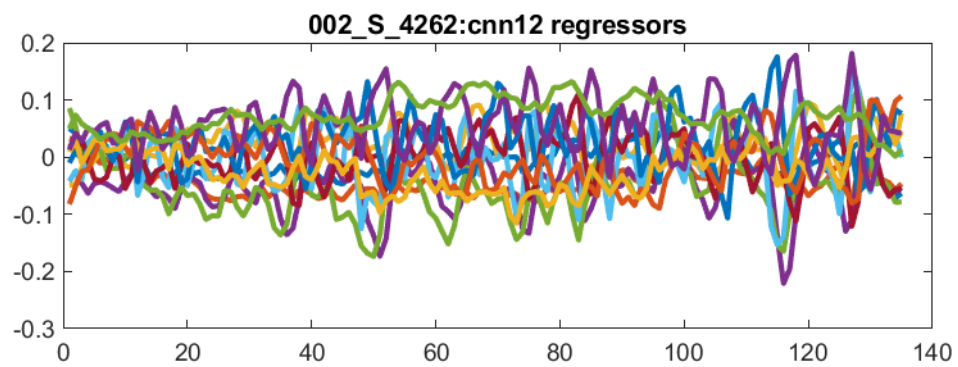

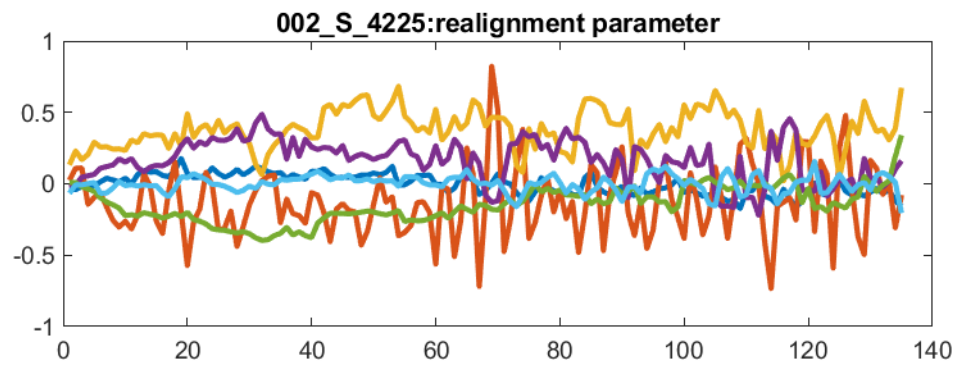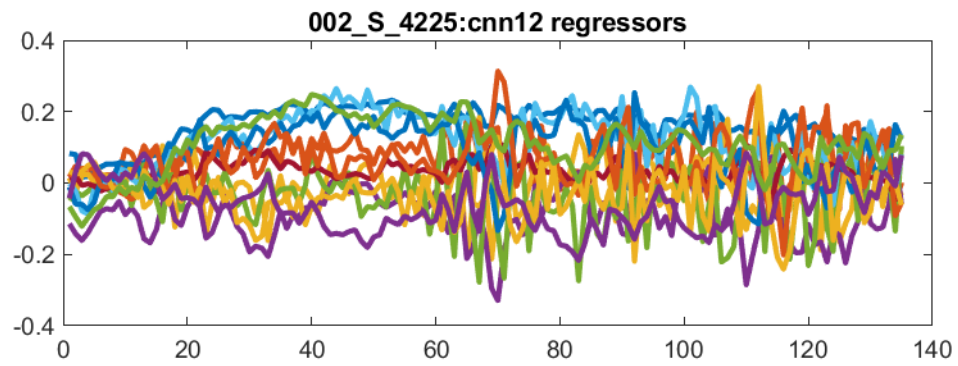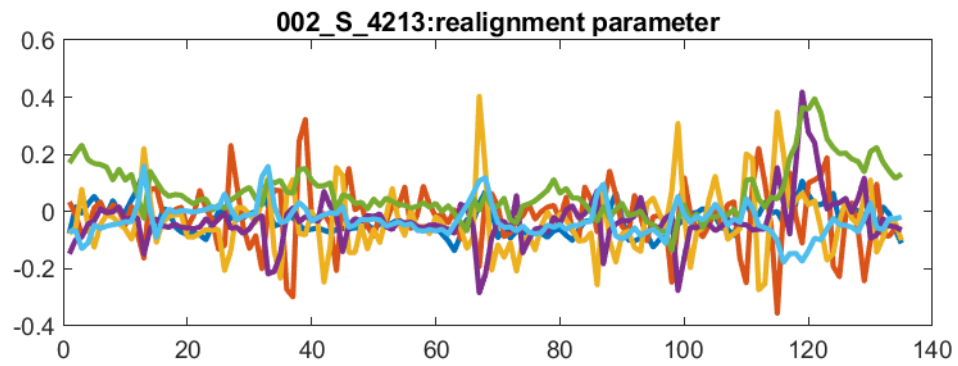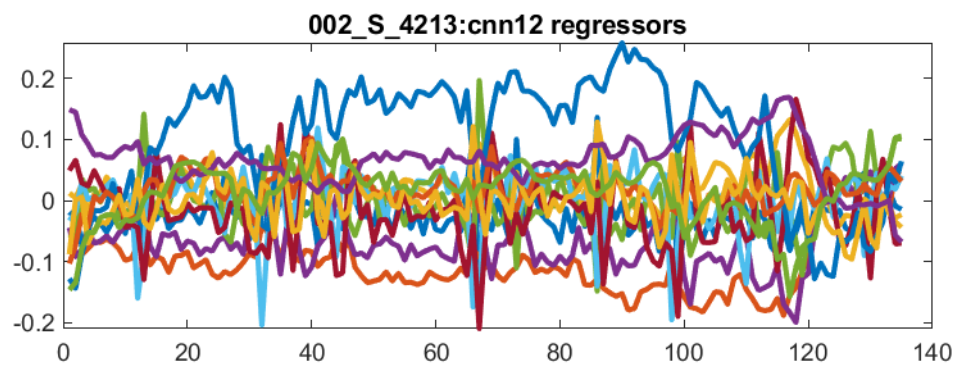

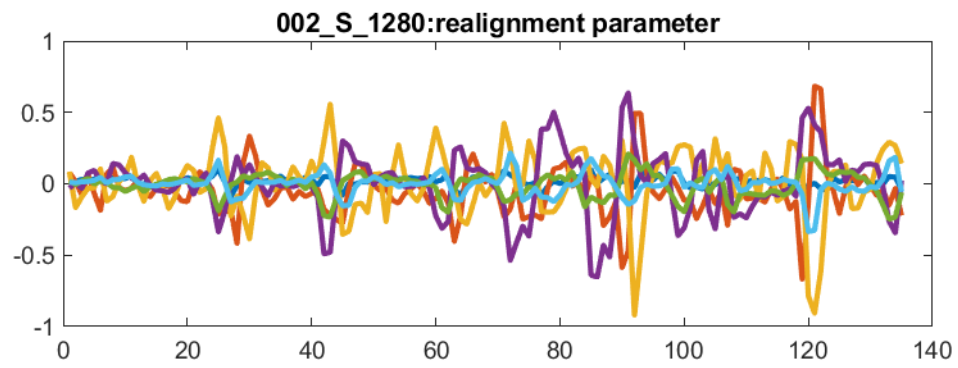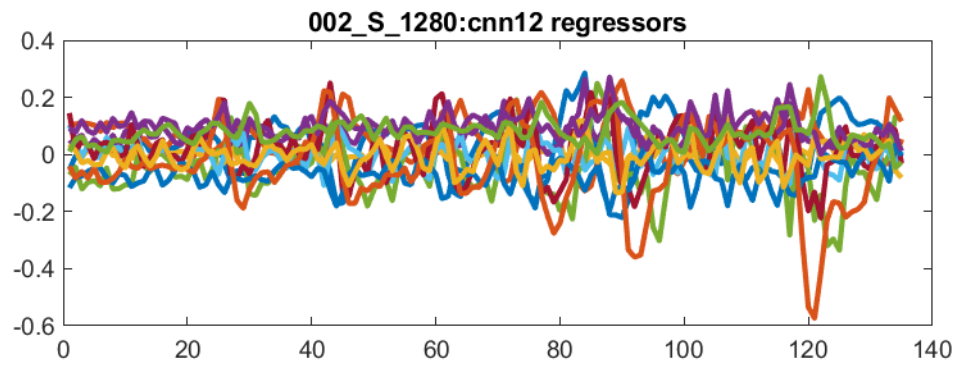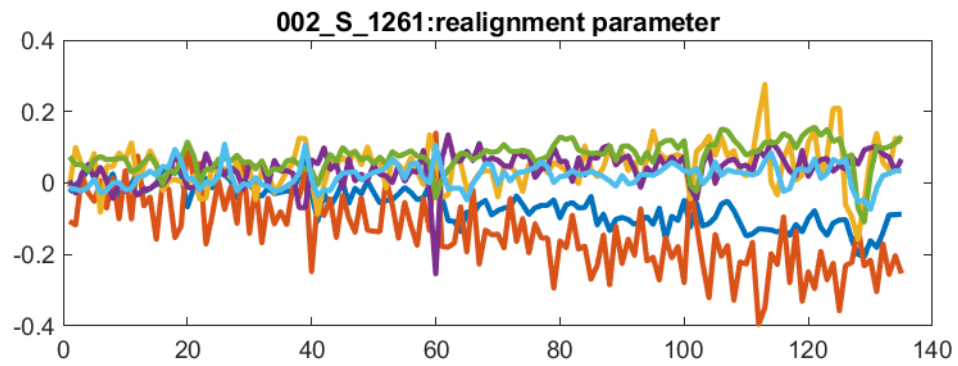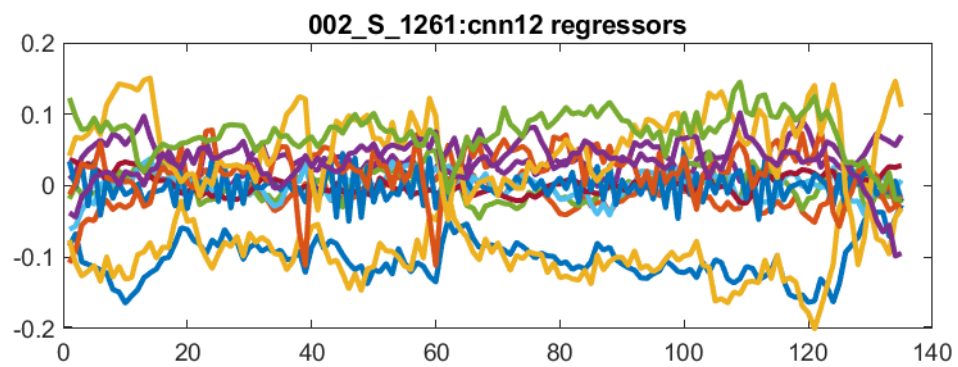

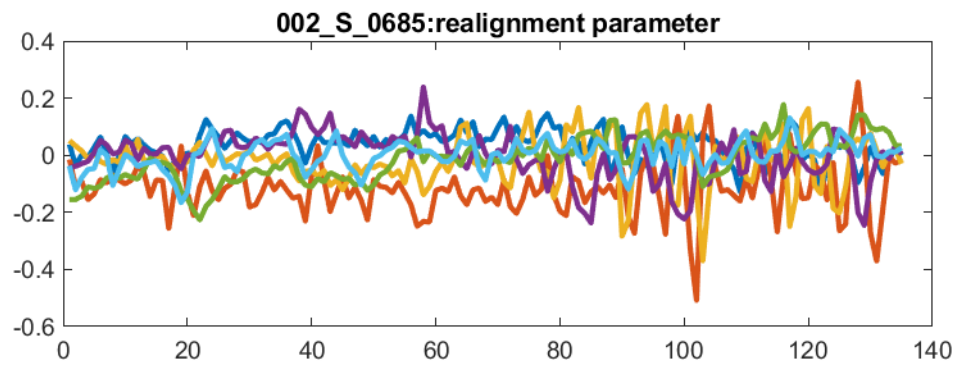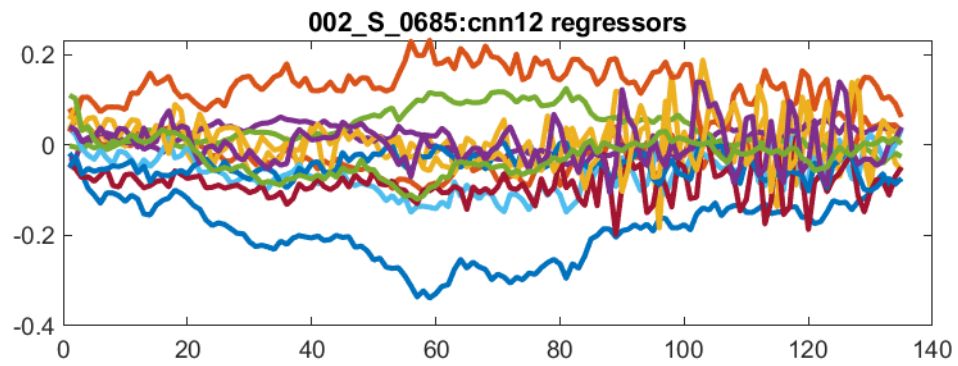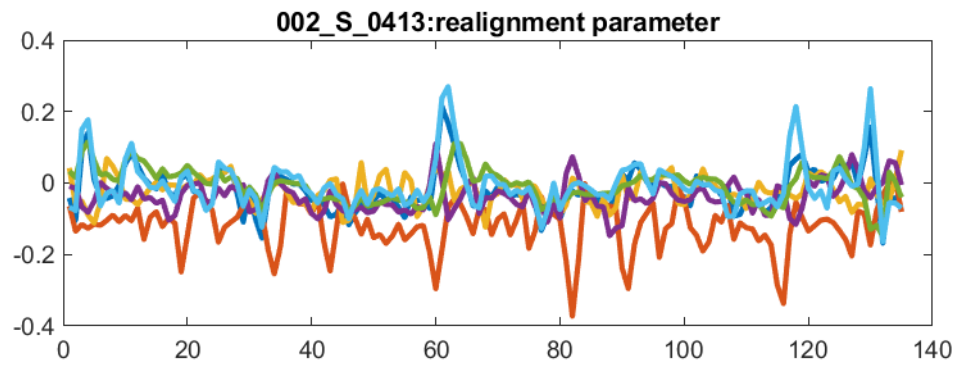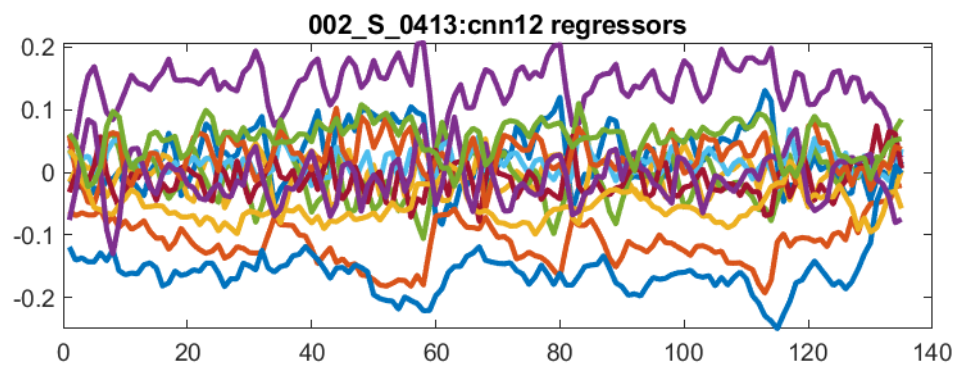

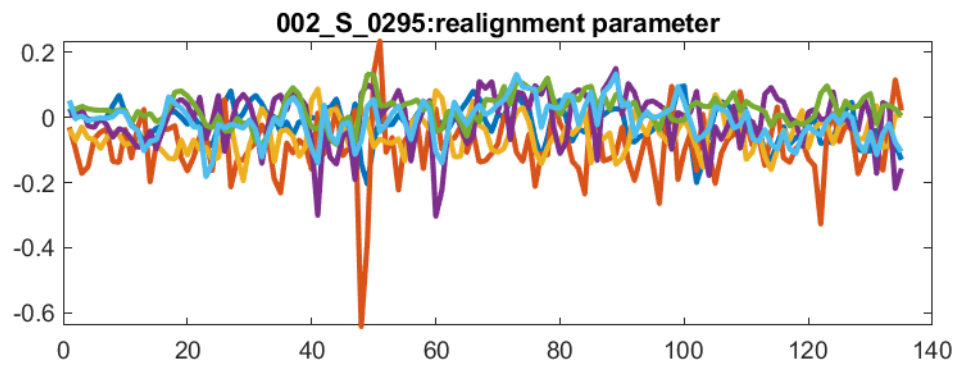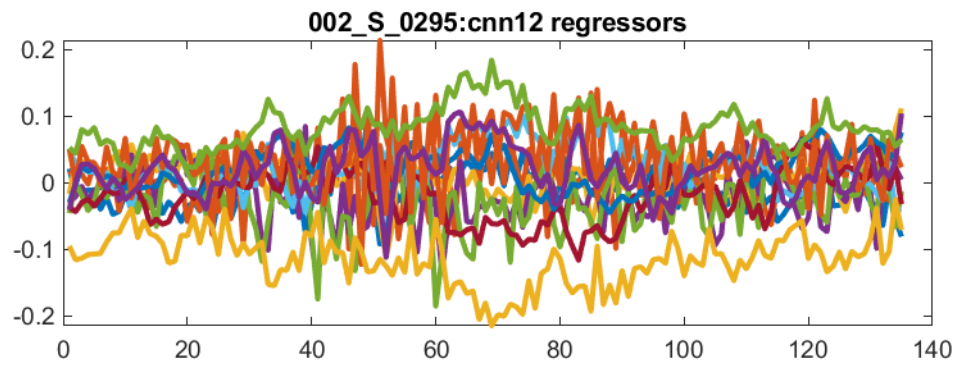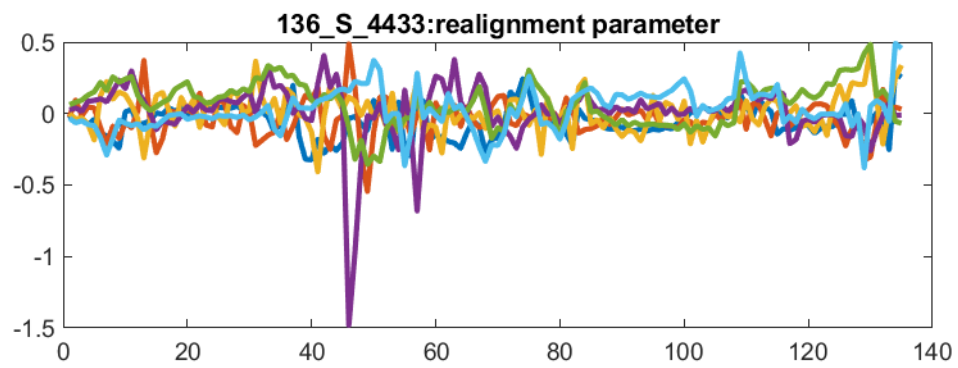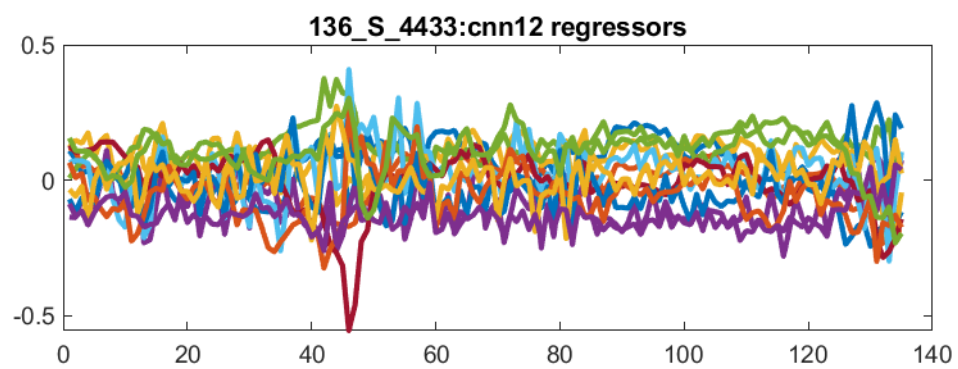

Supplement: Supplementary file 1 [file Data_Sheet_1.ZIP › supplementary/cnnRegressors.pdf]

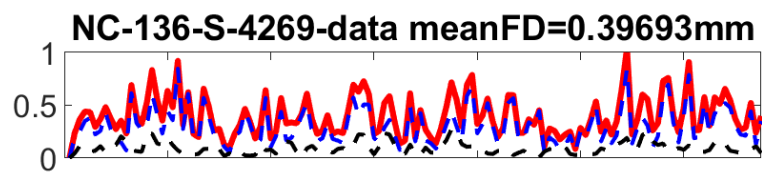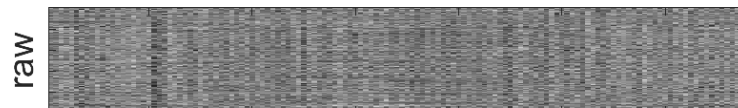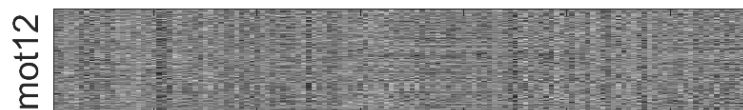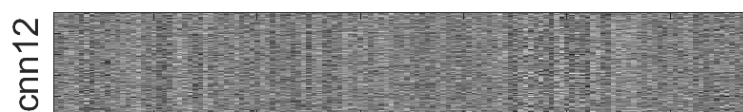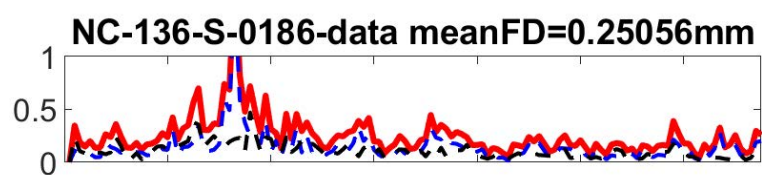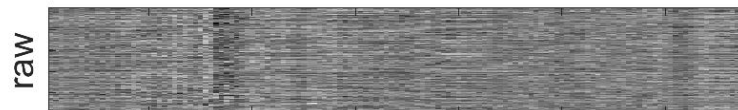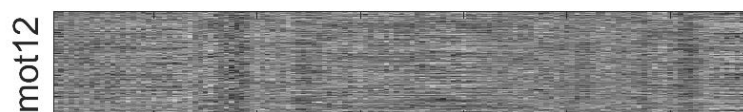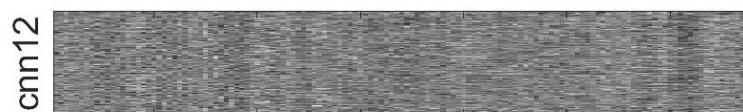

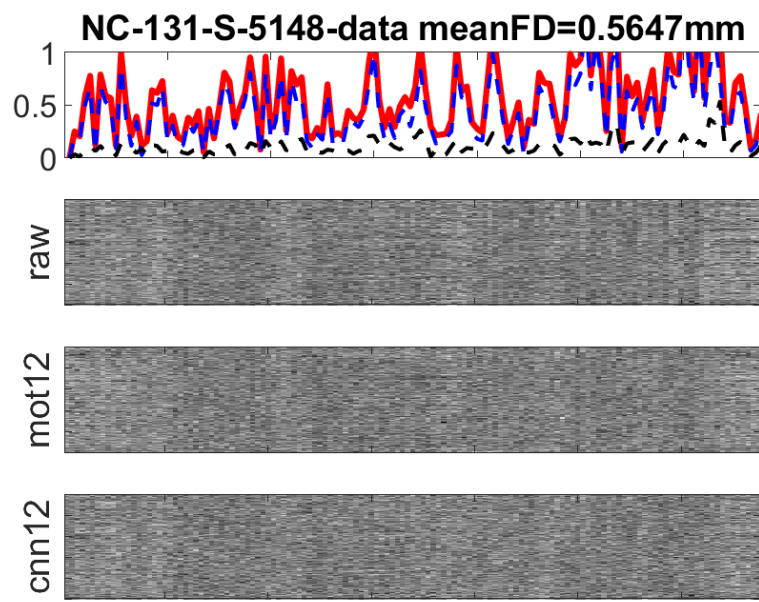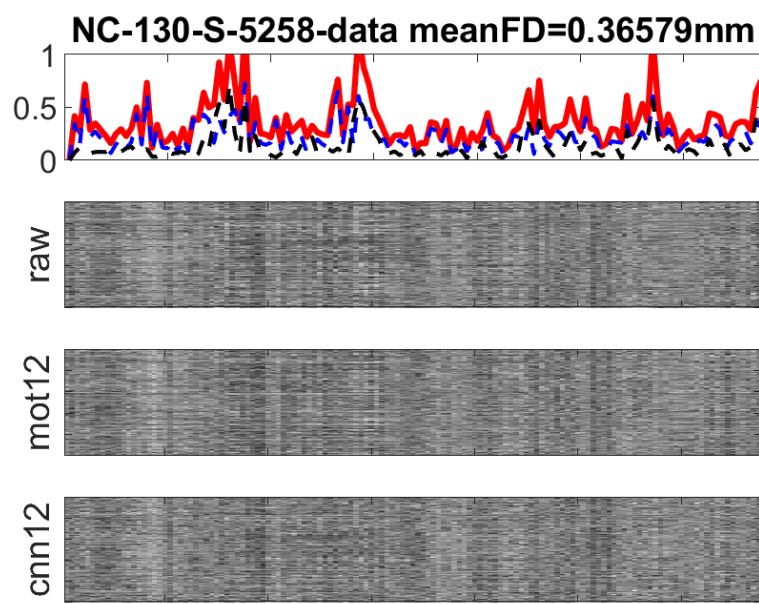

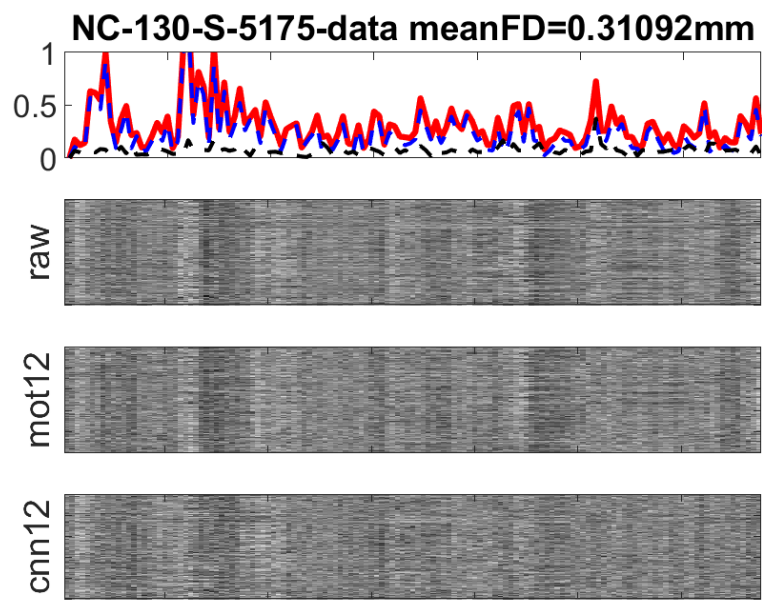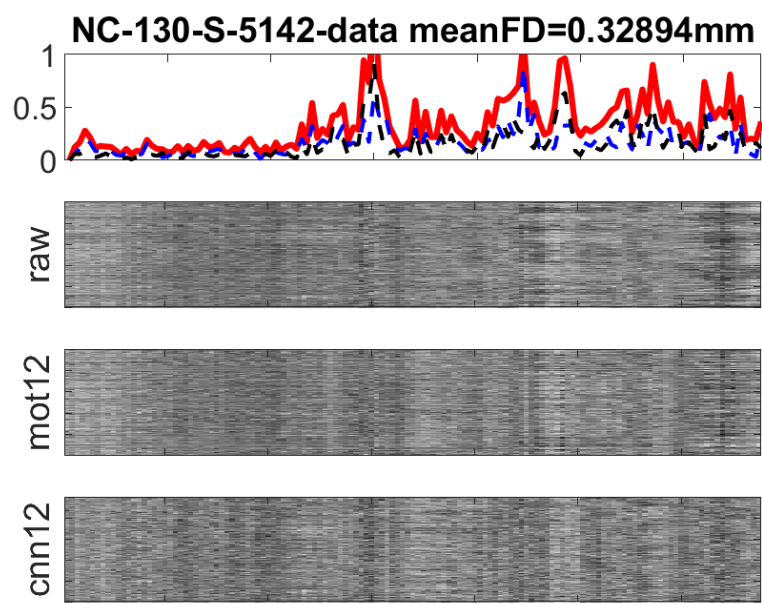

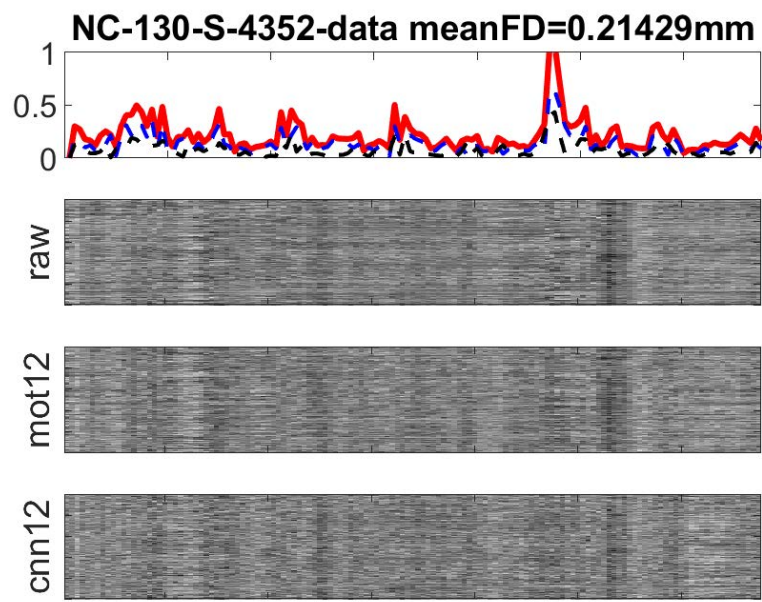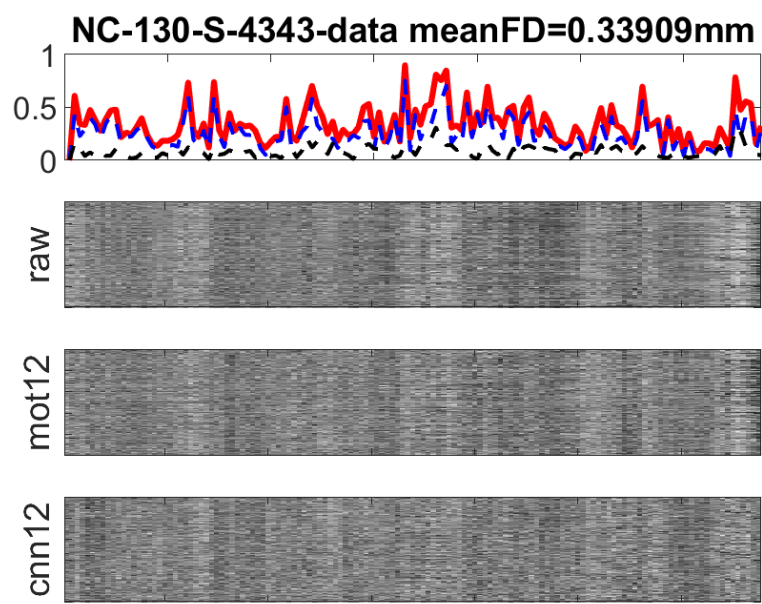

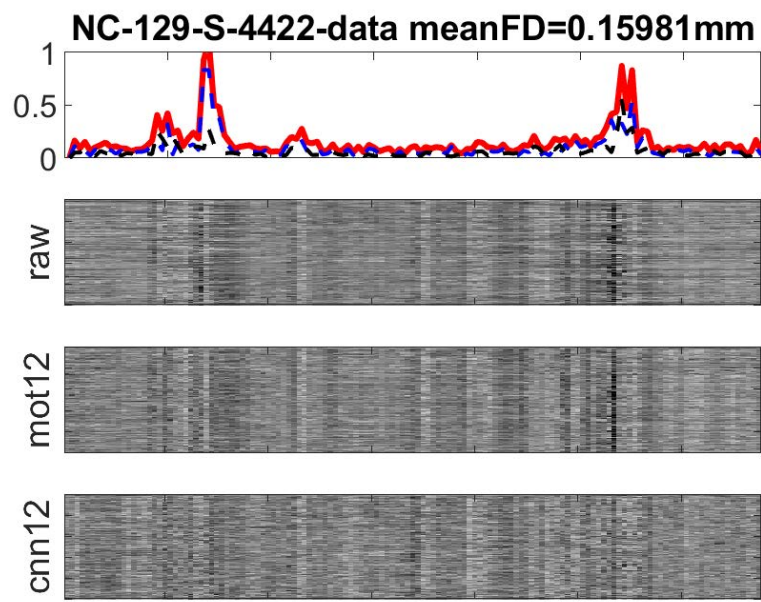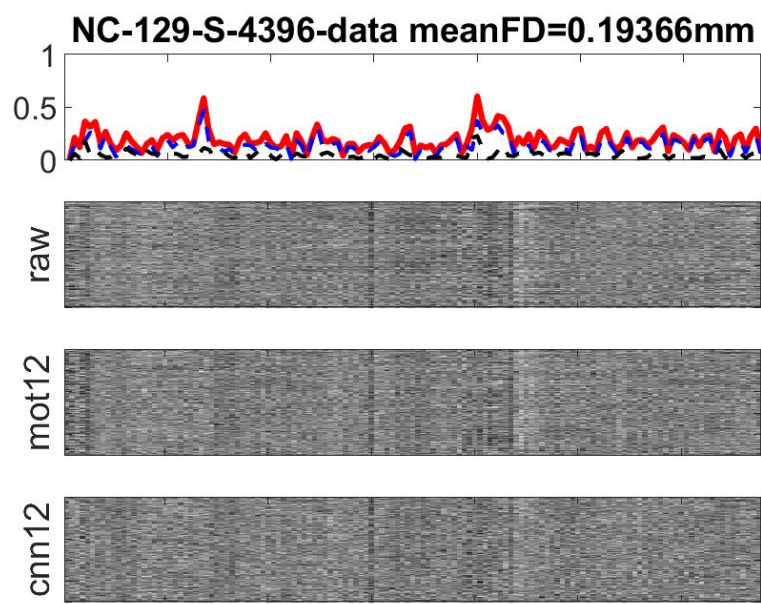

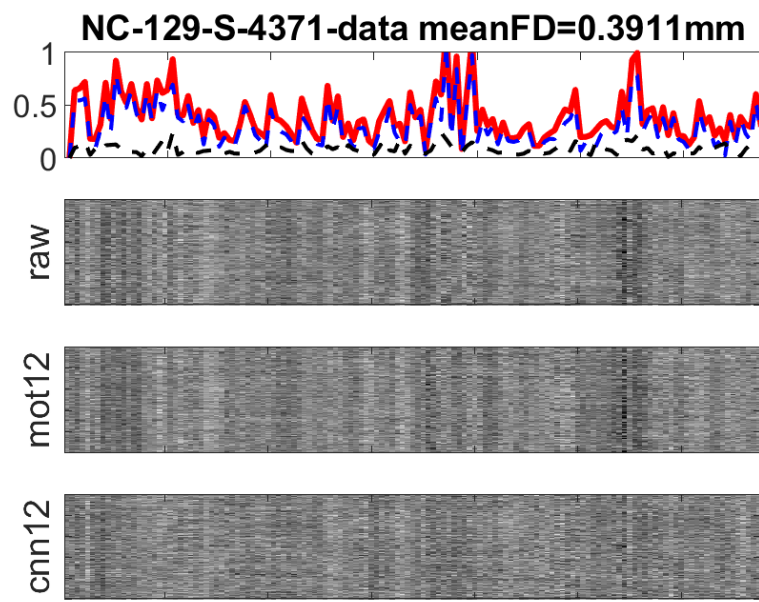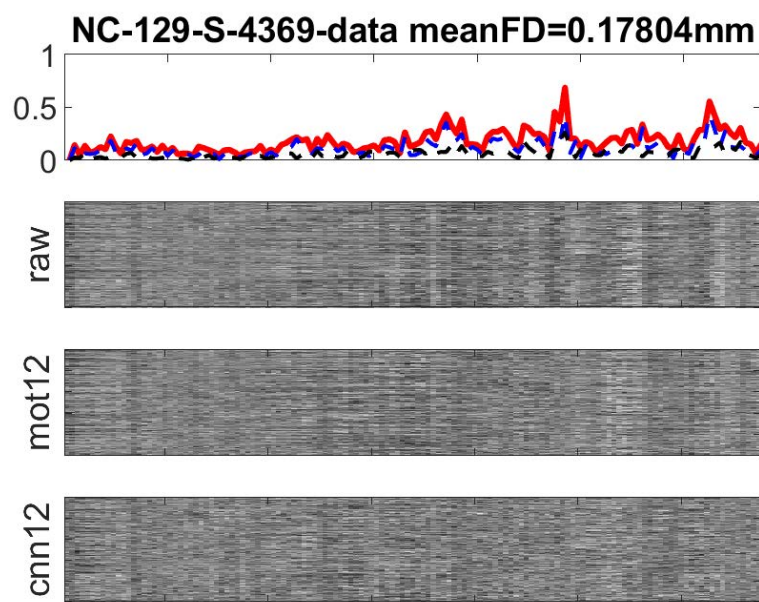

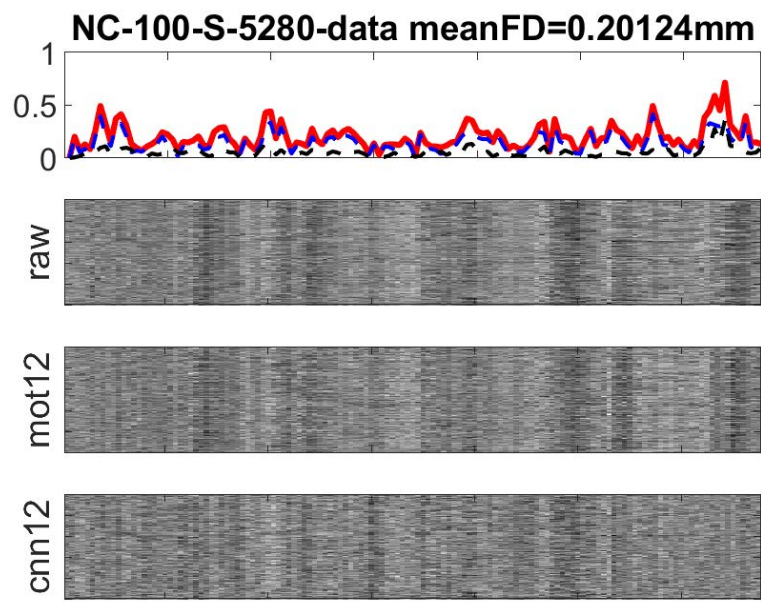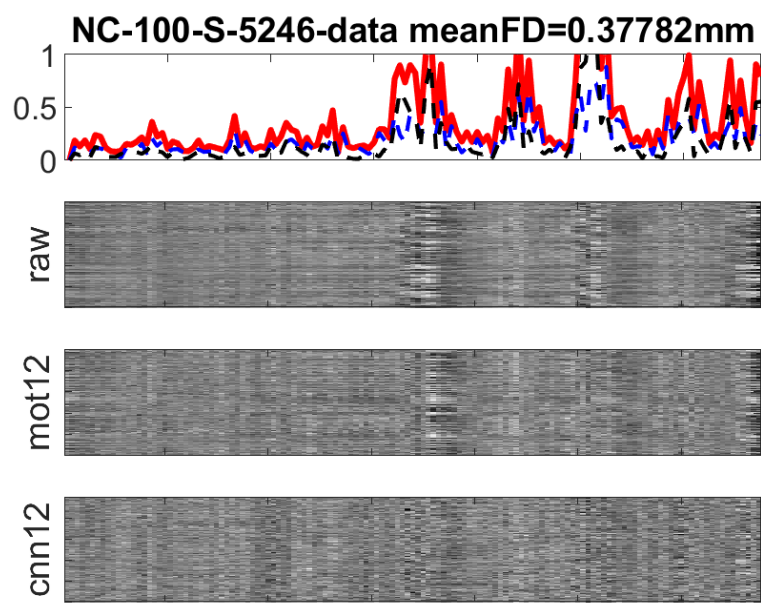

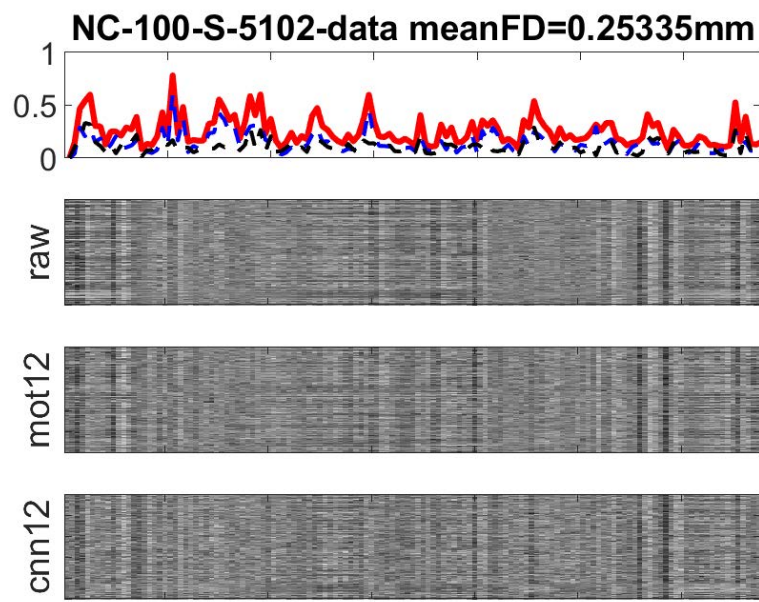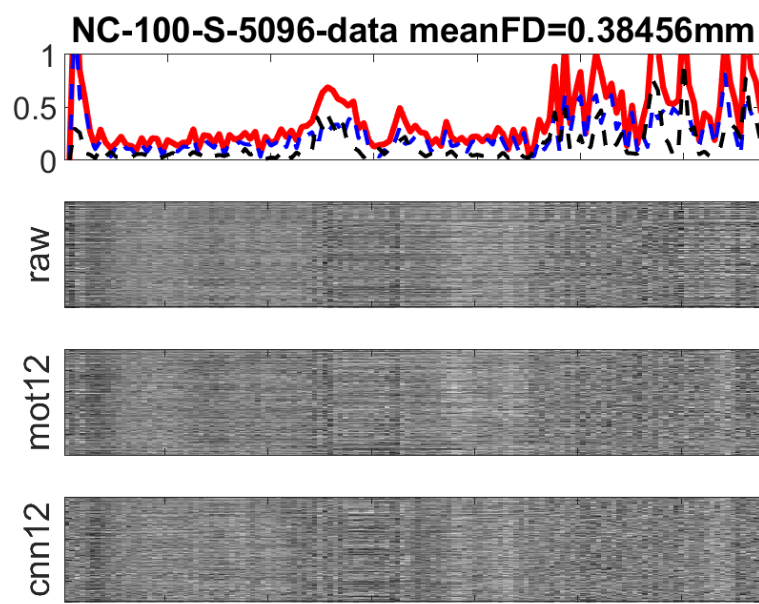

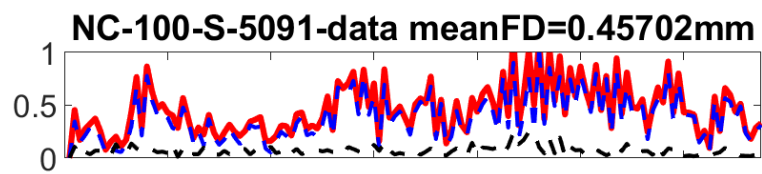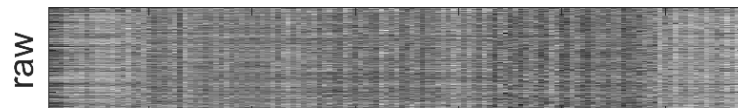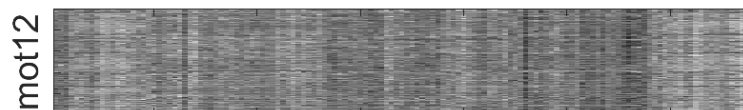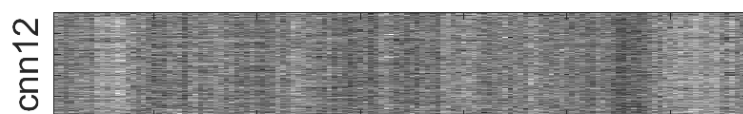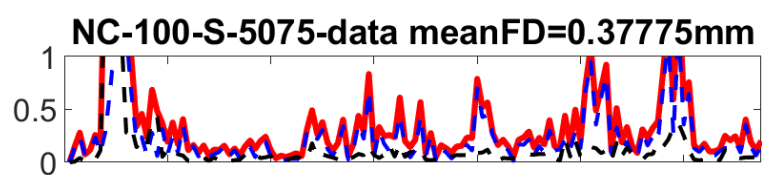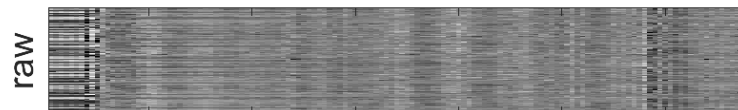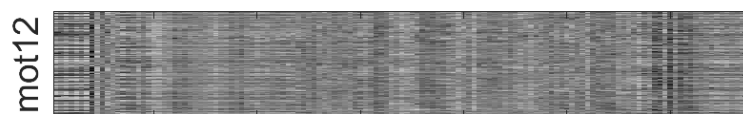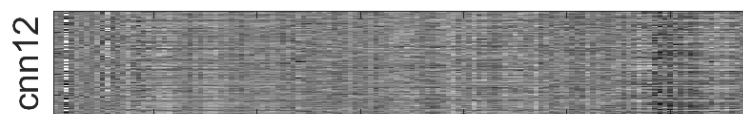

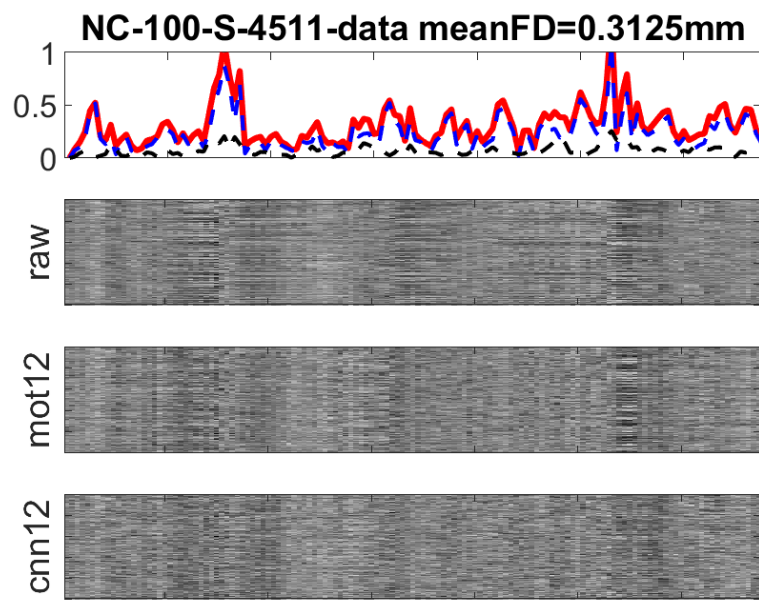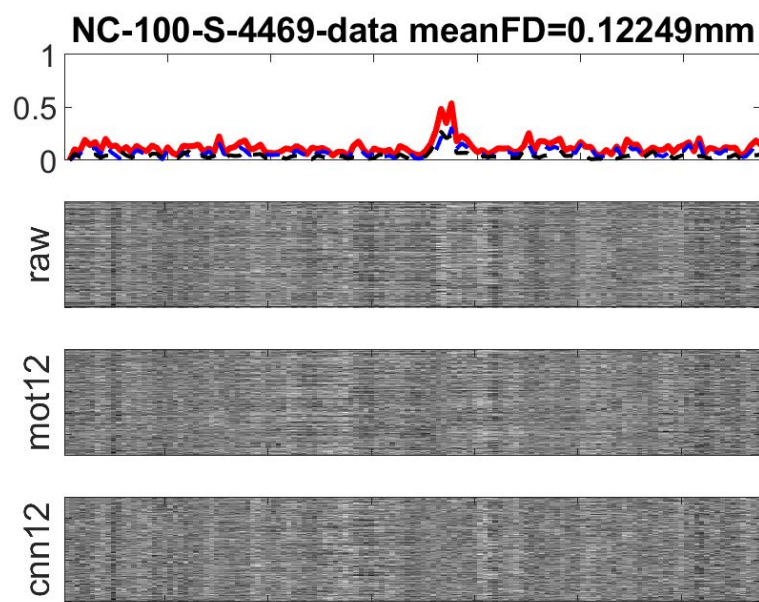

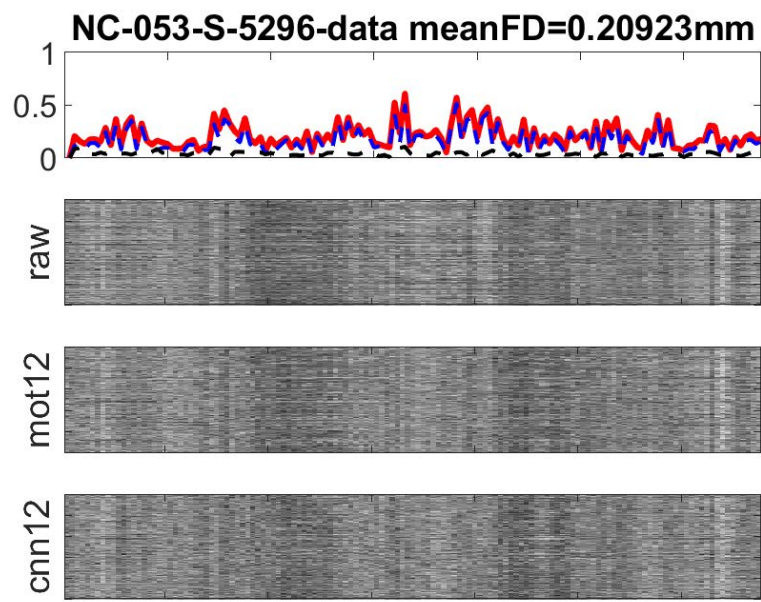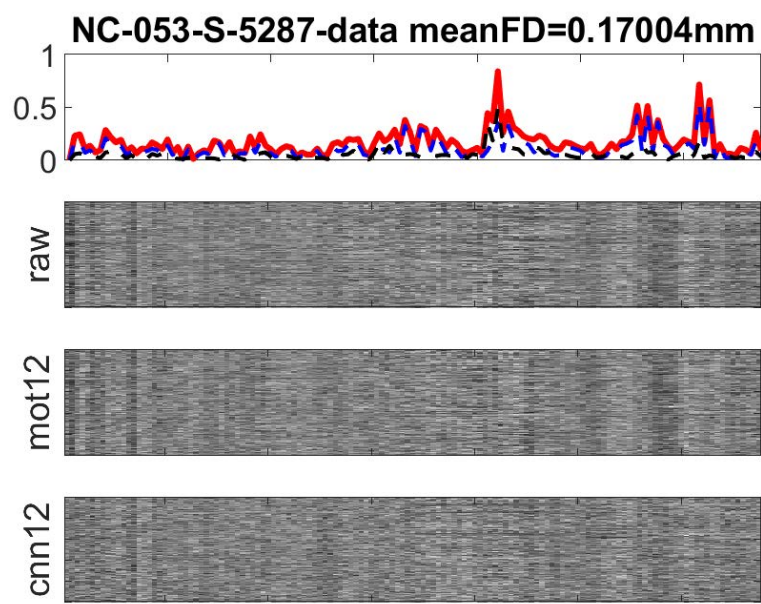

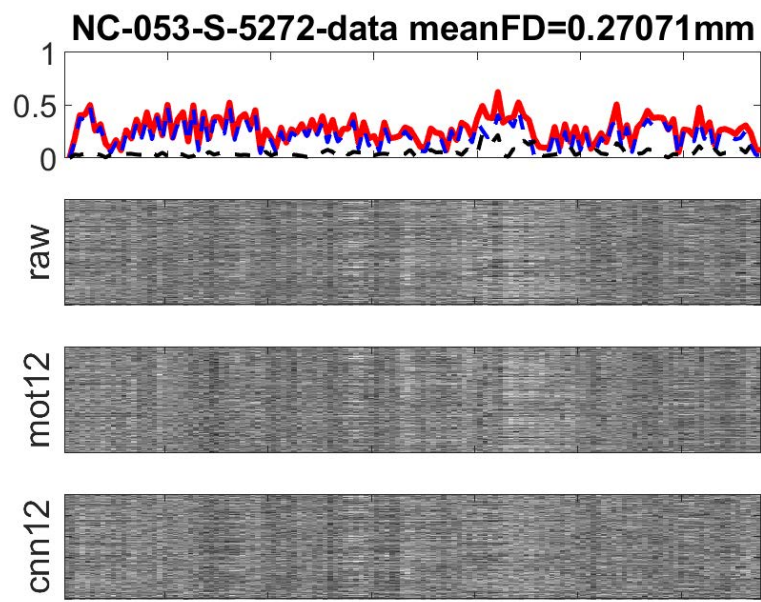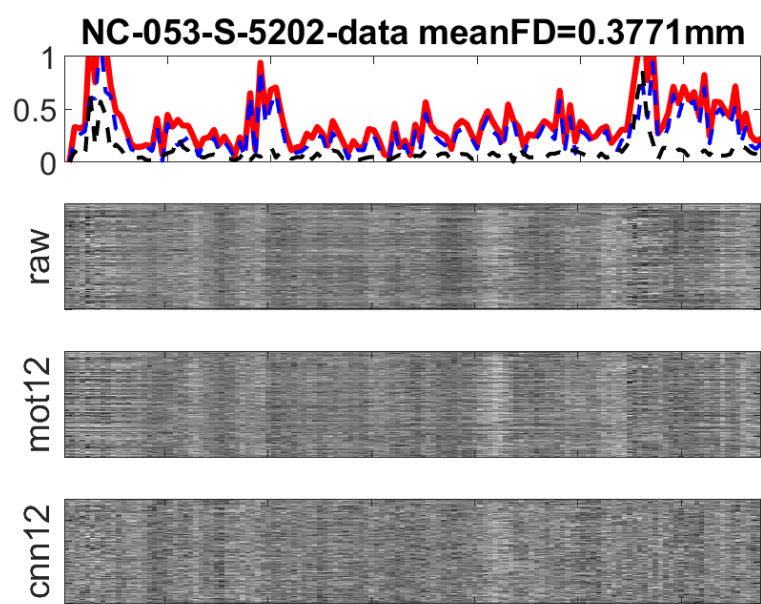

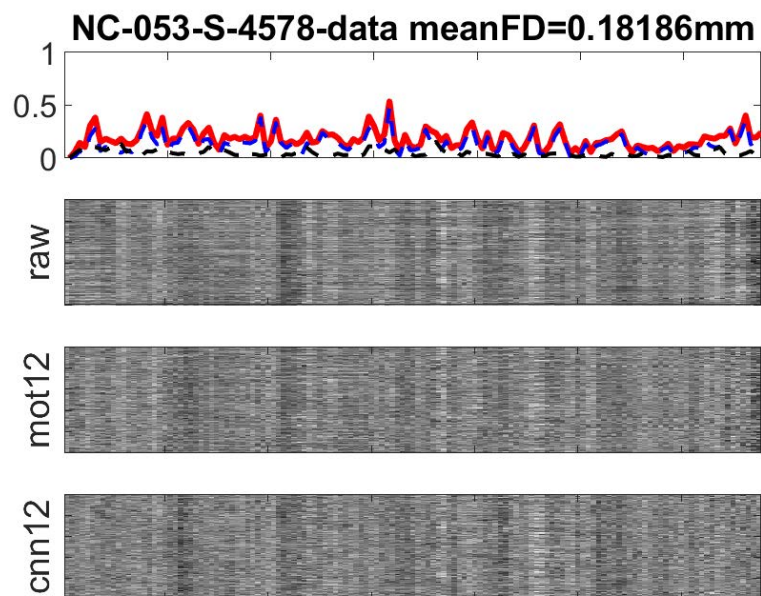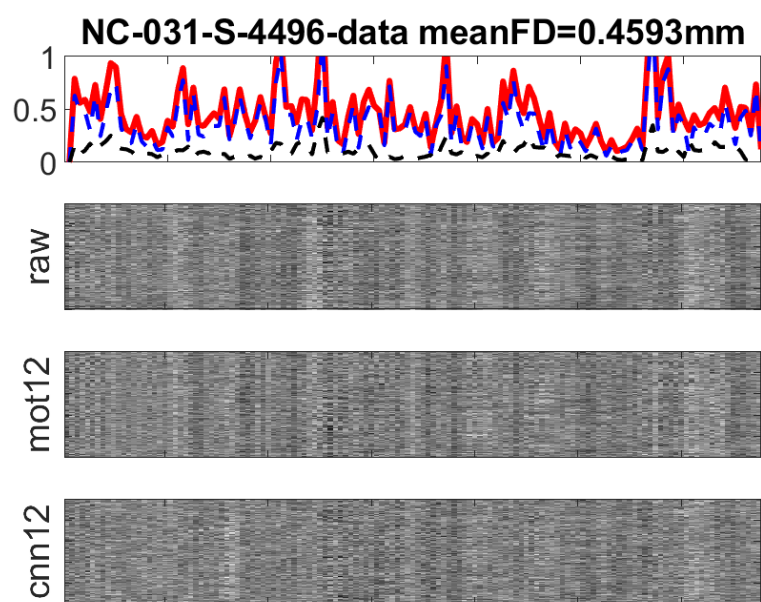

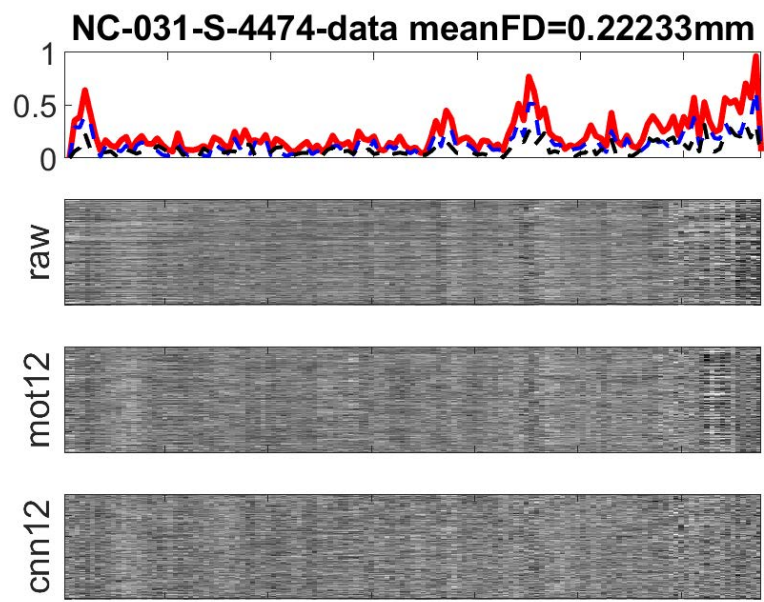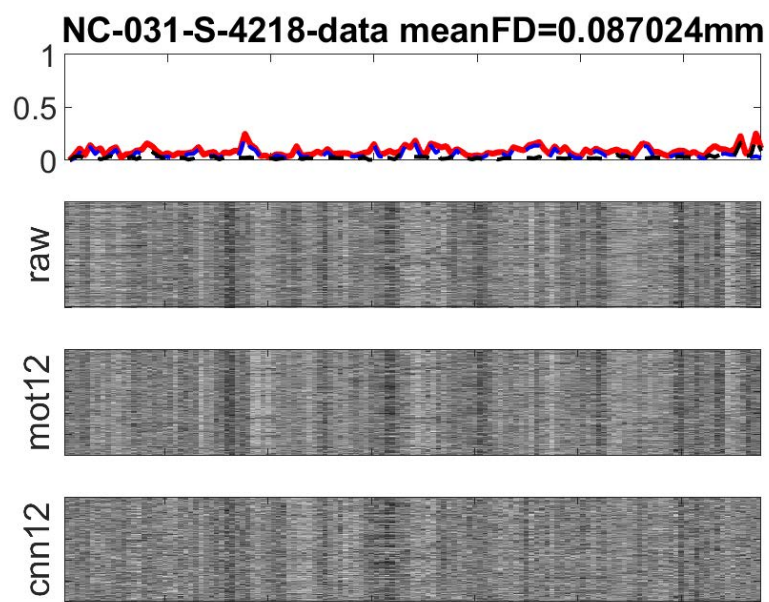

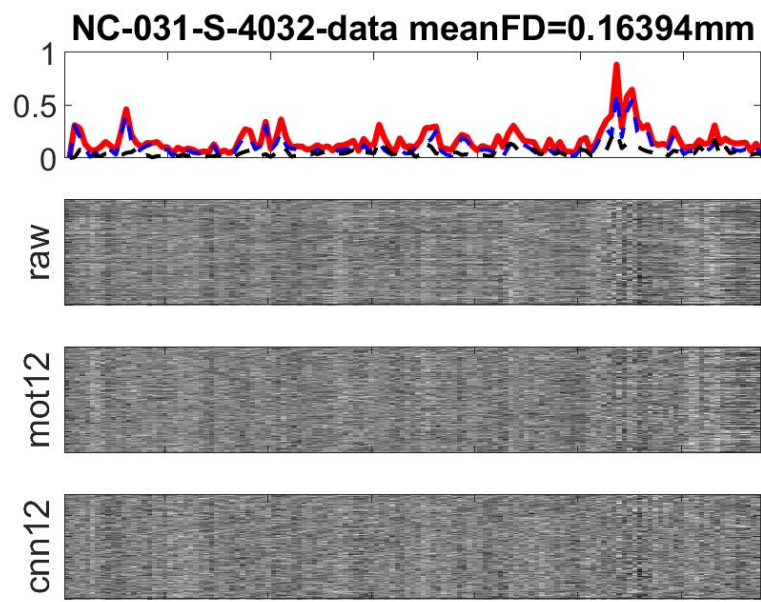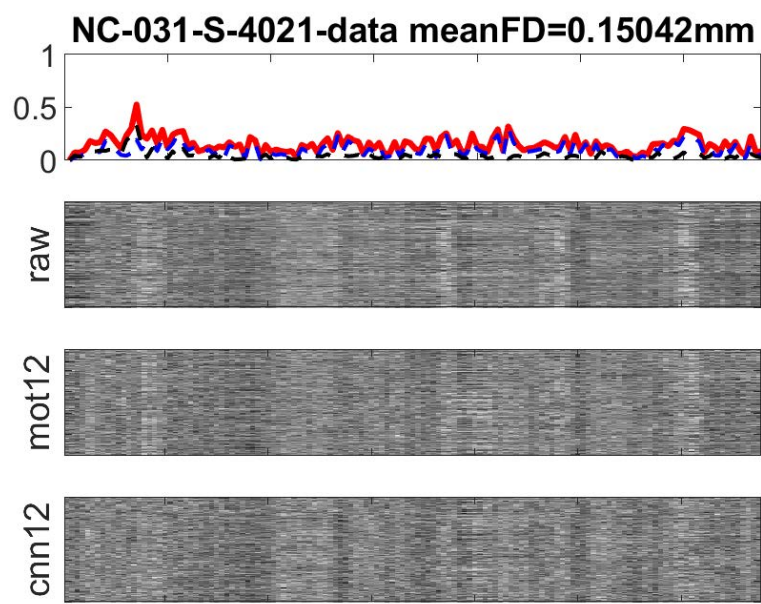

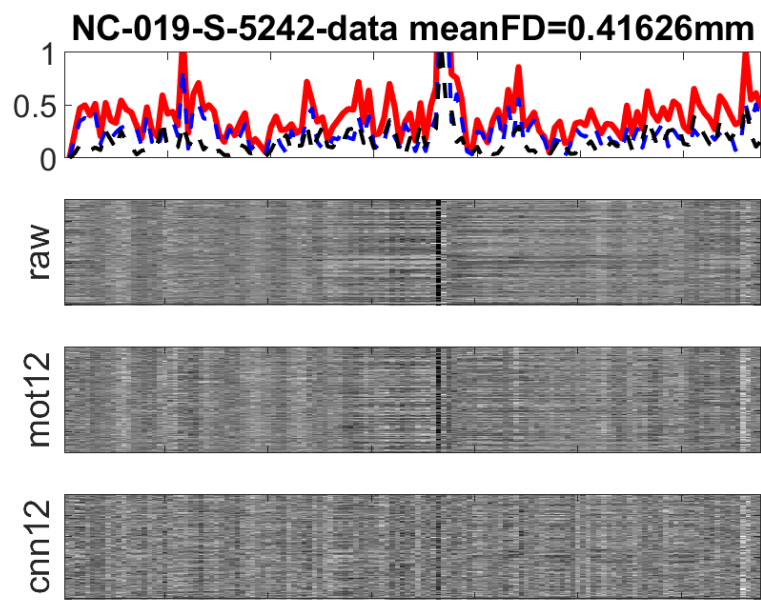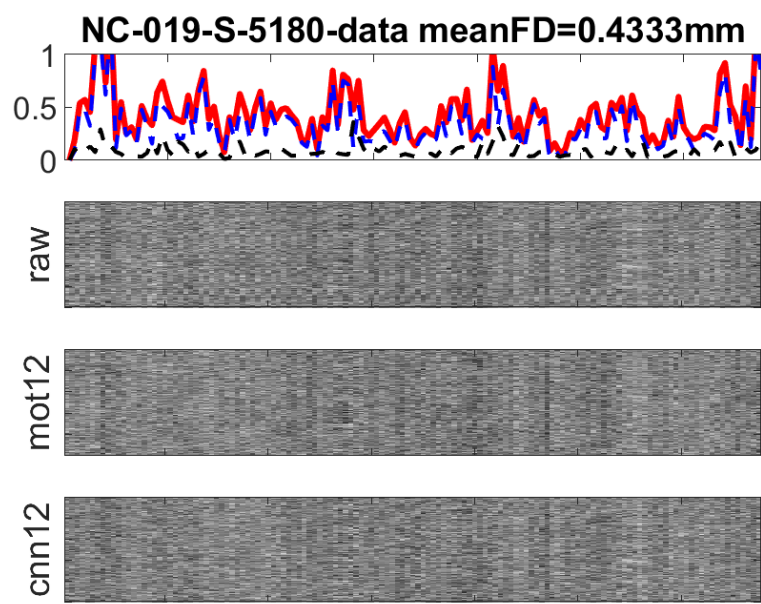

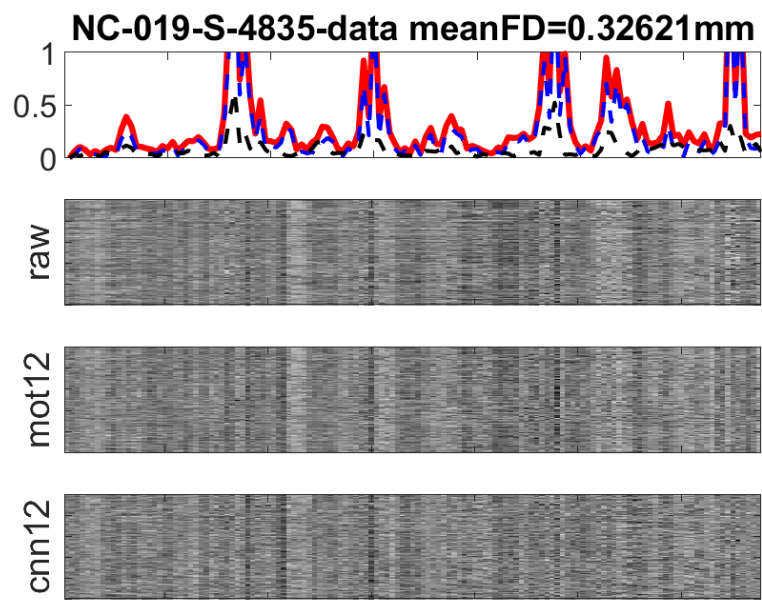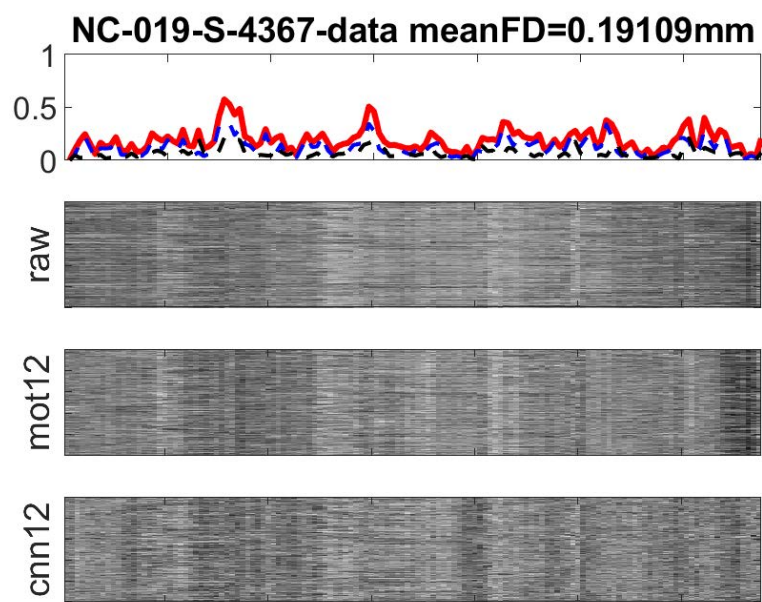

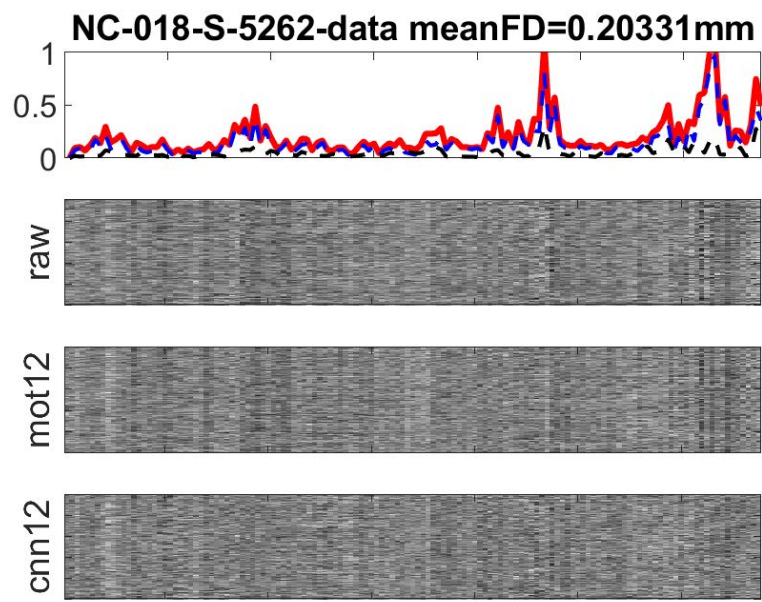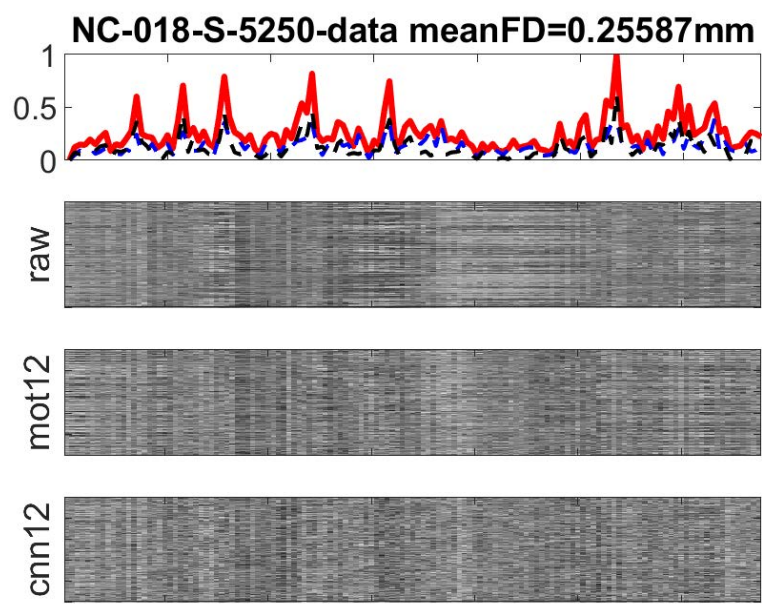

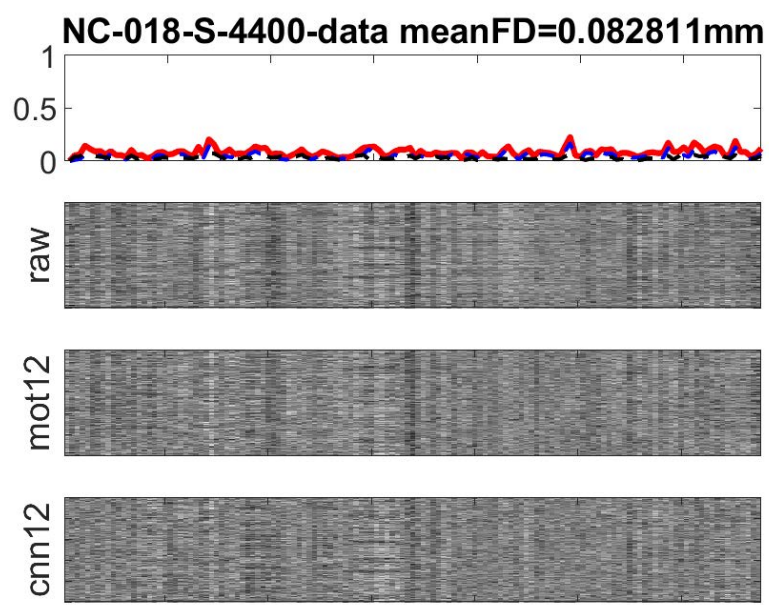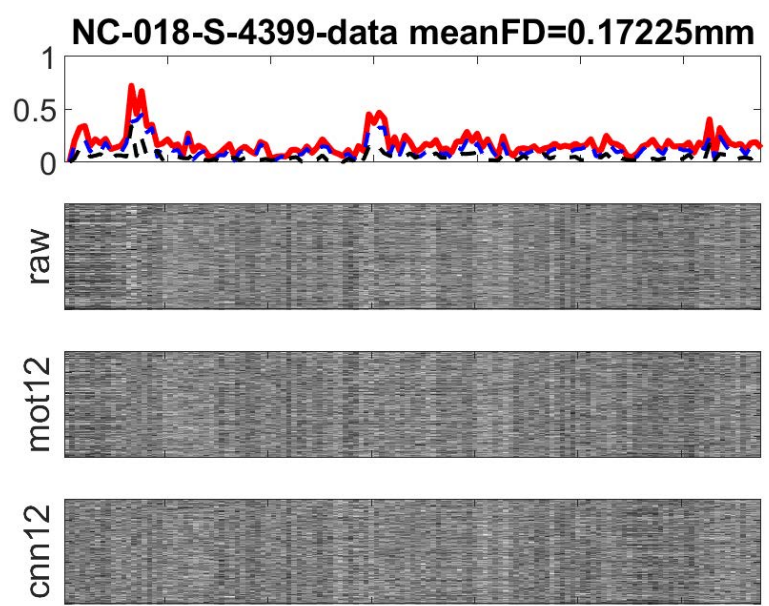

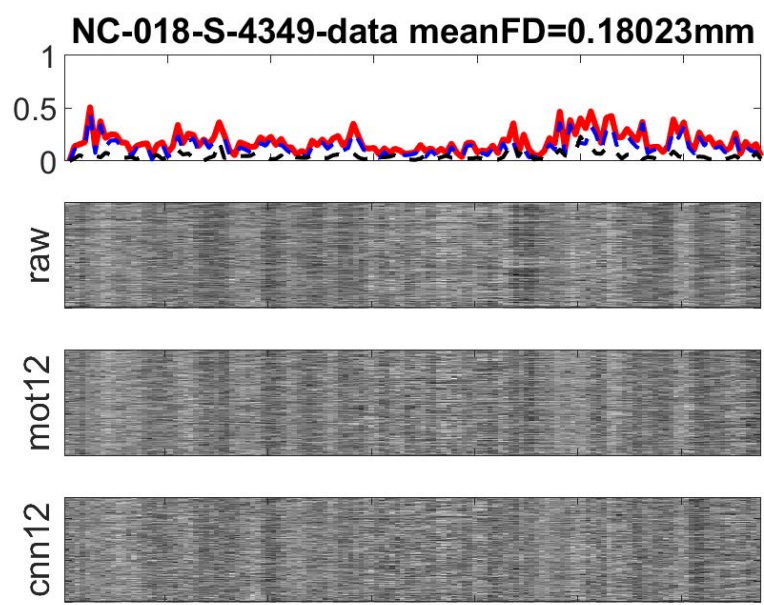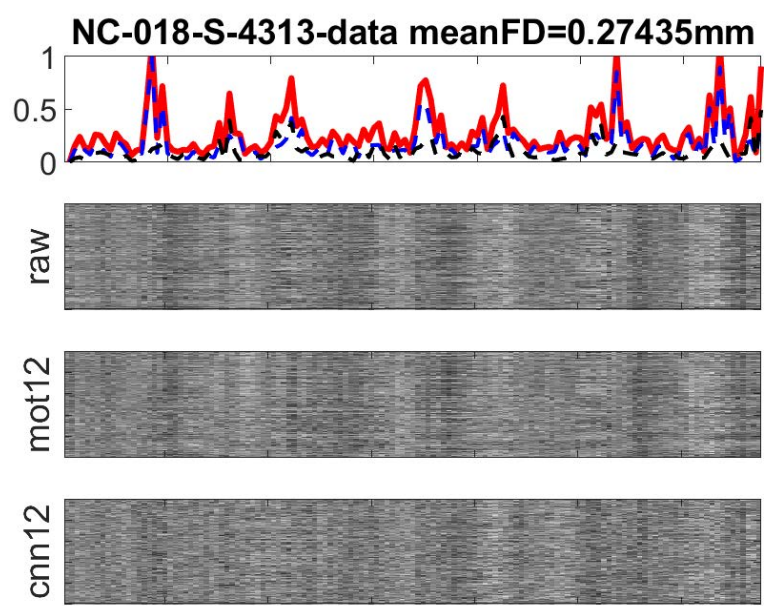

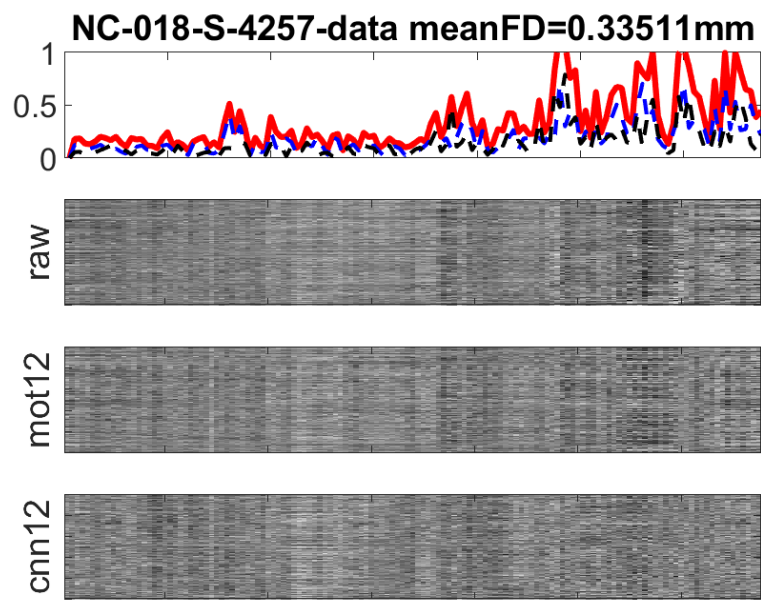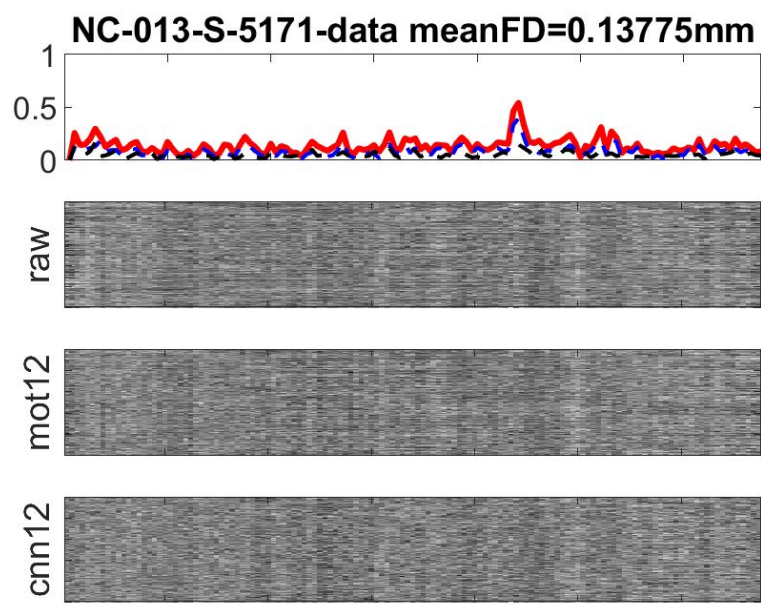

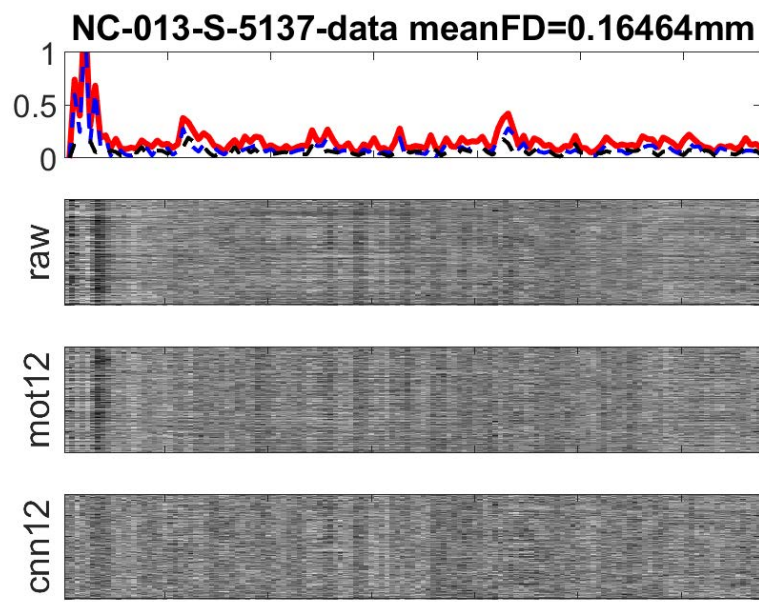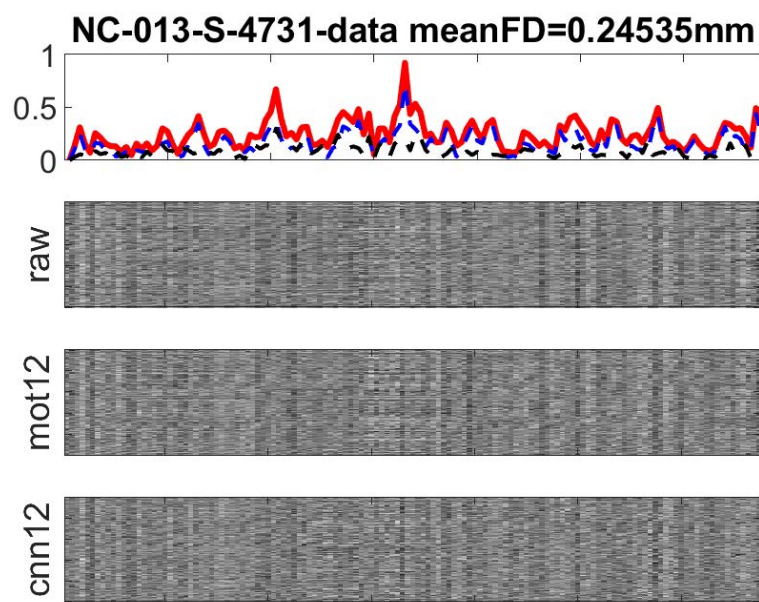

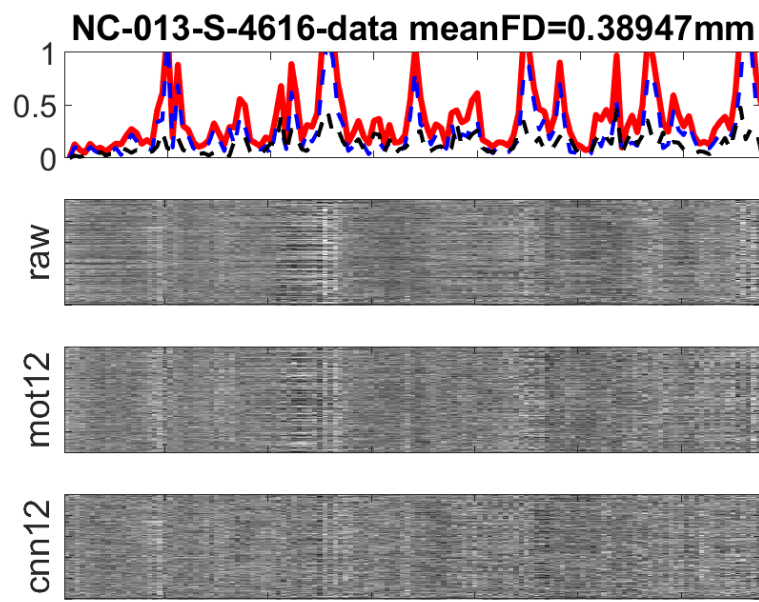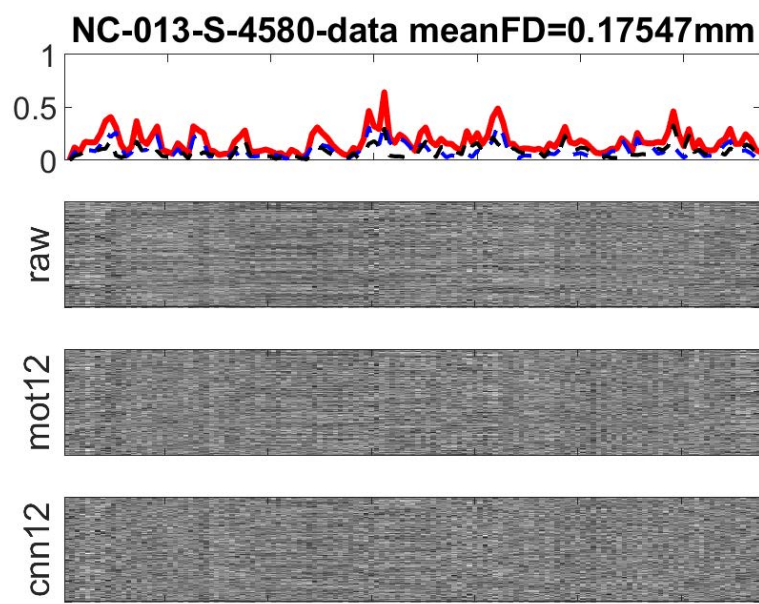

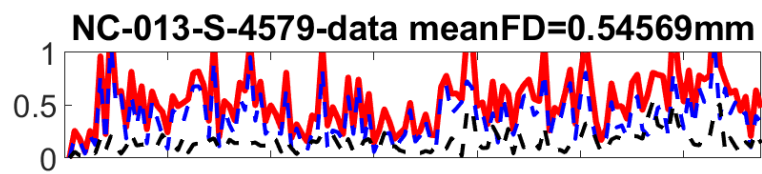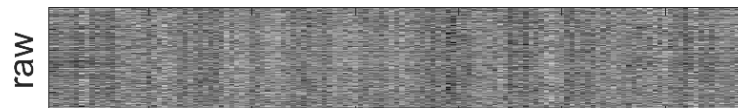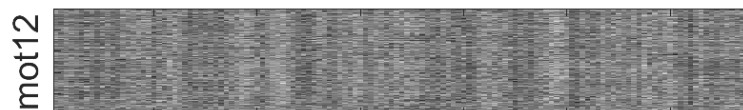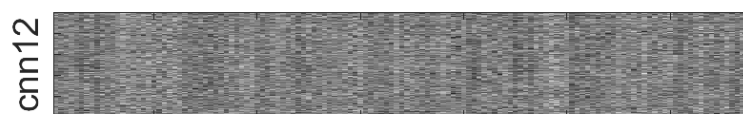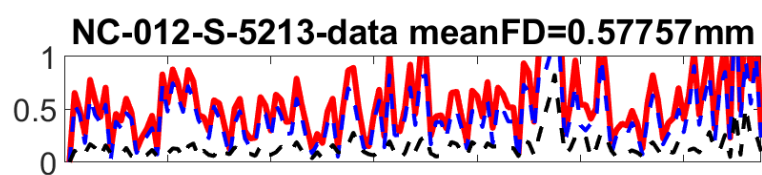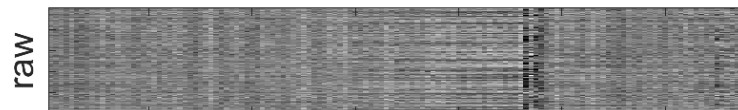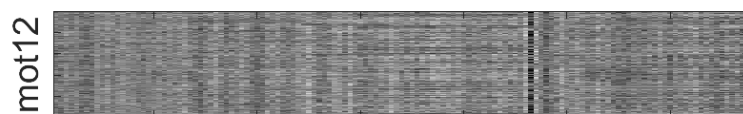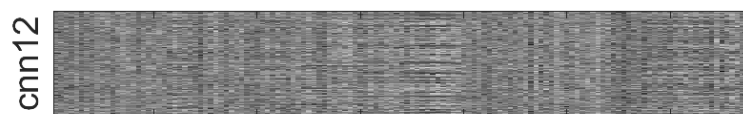

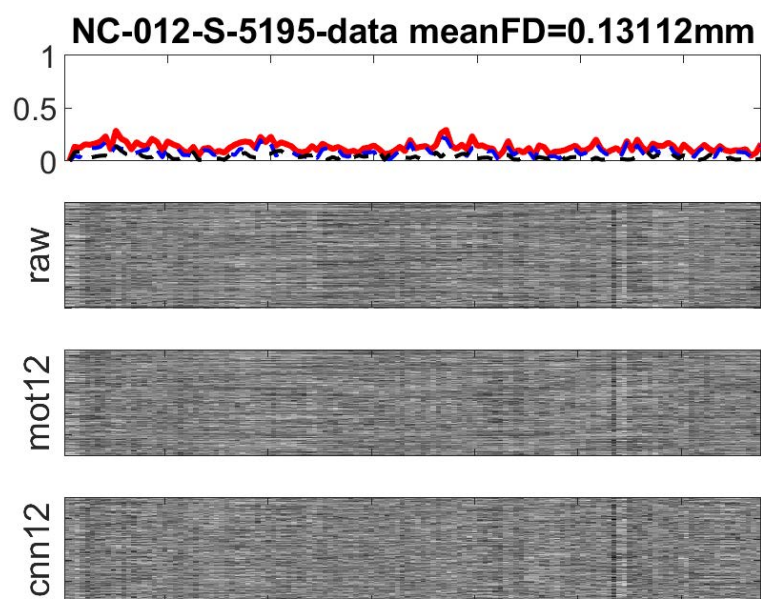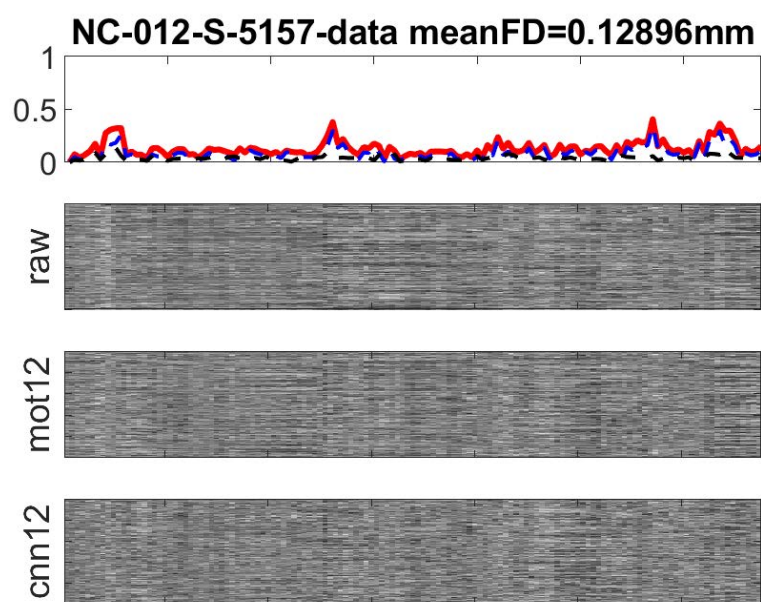

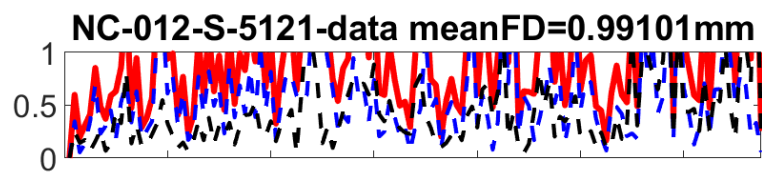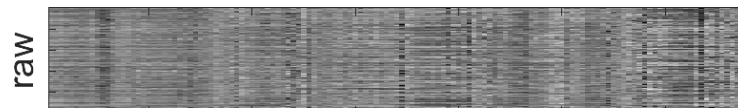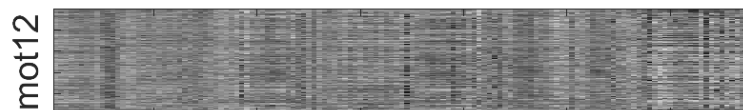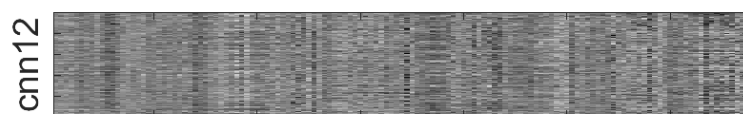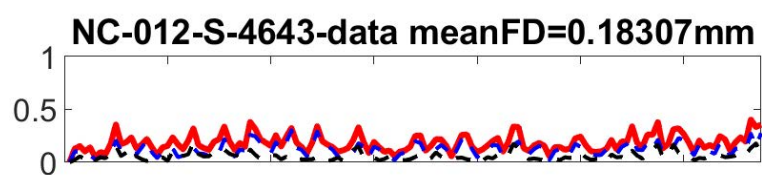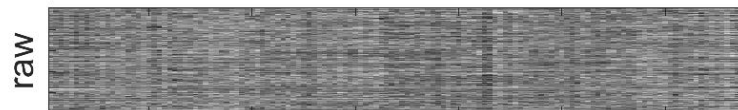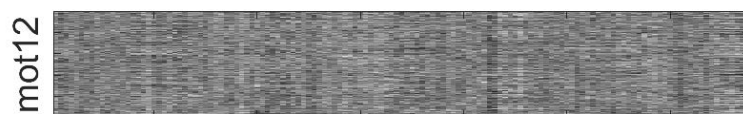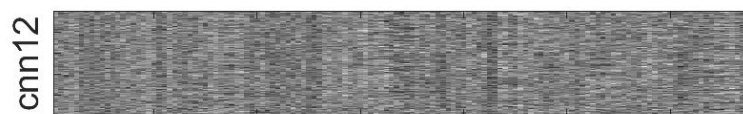

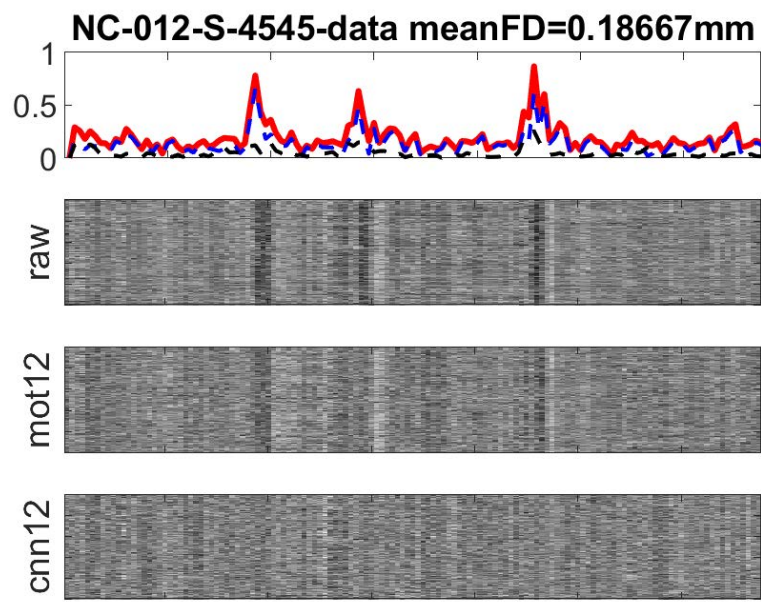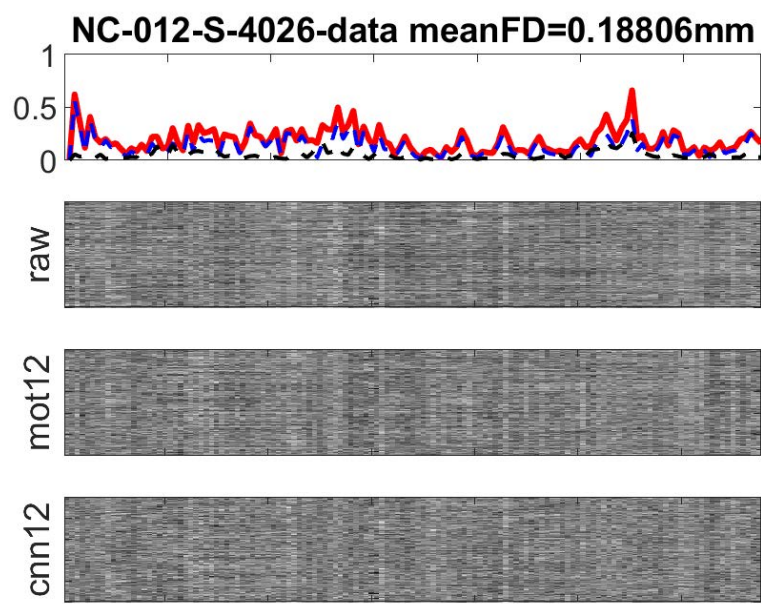

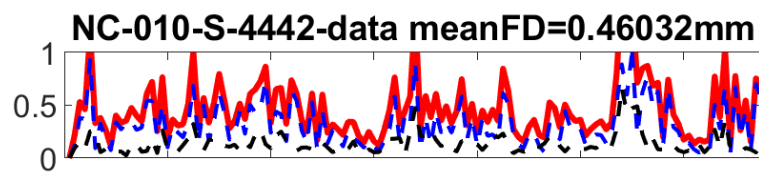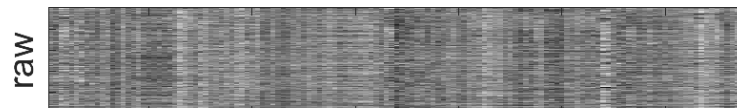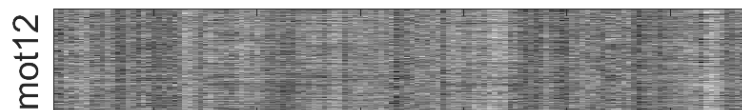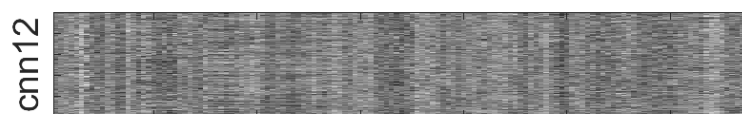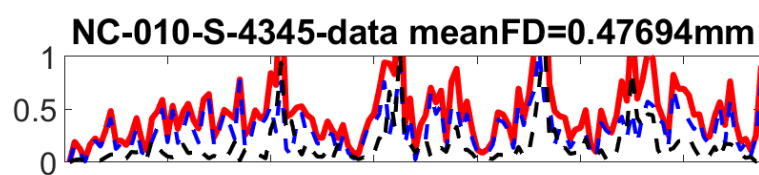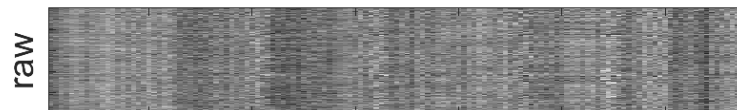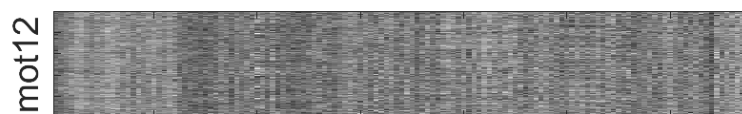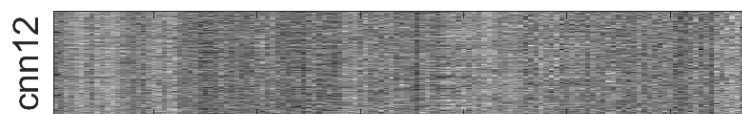

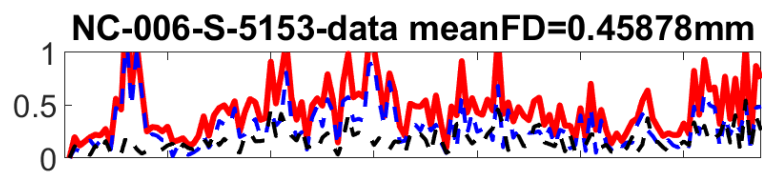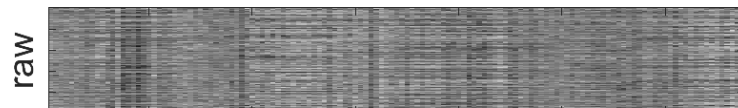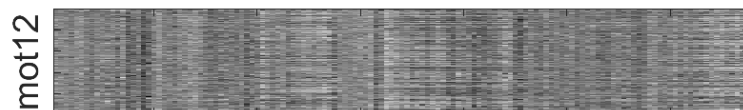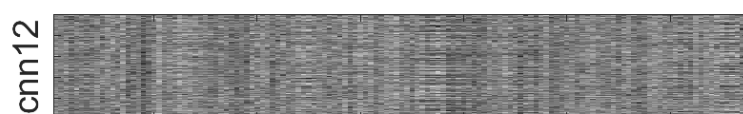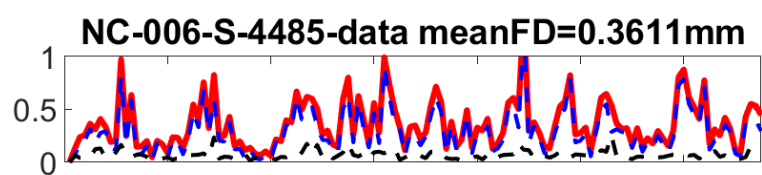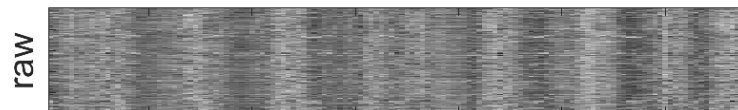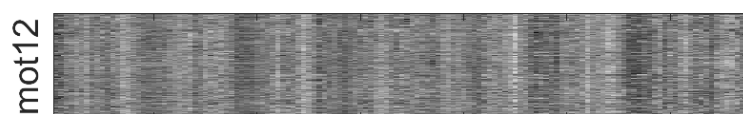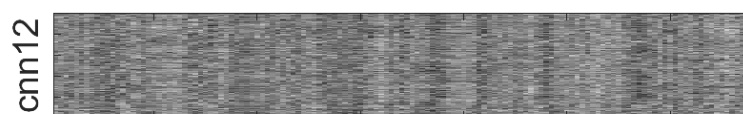

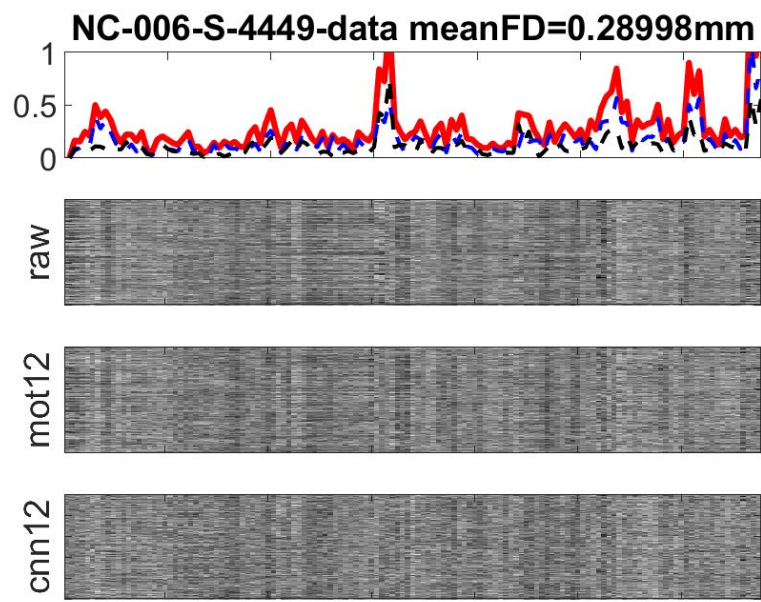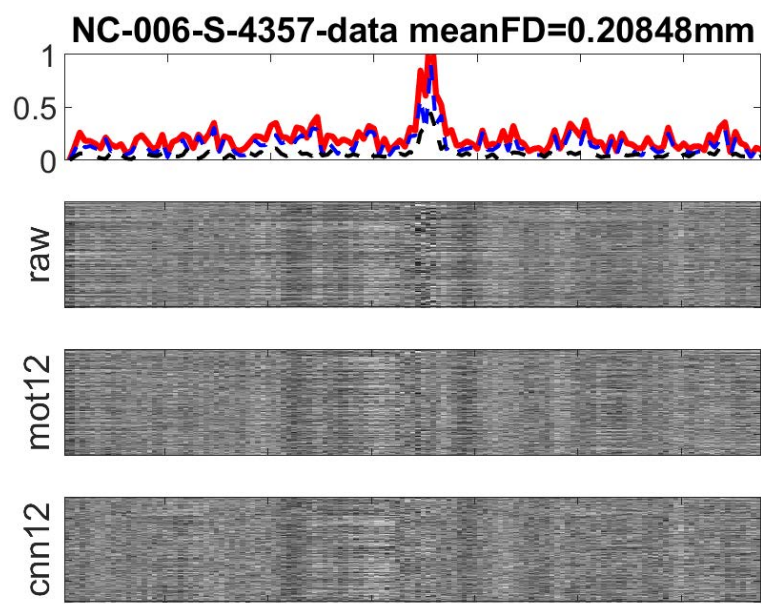

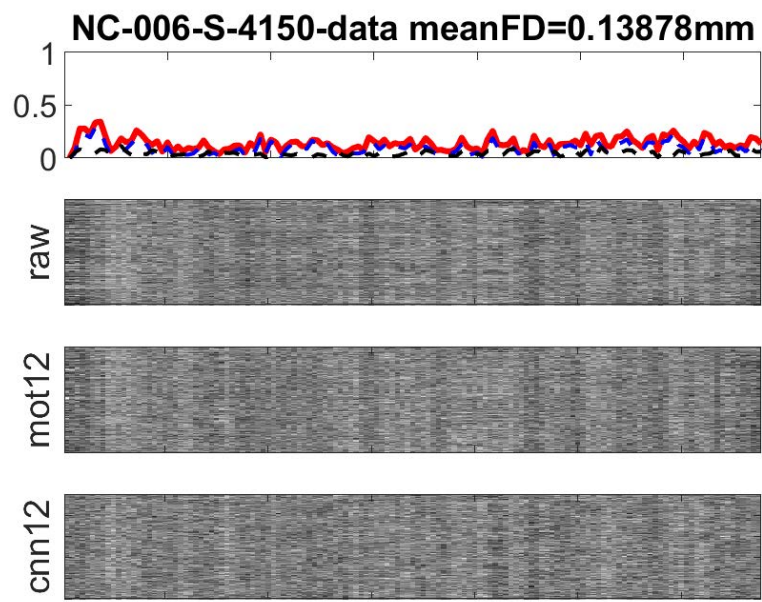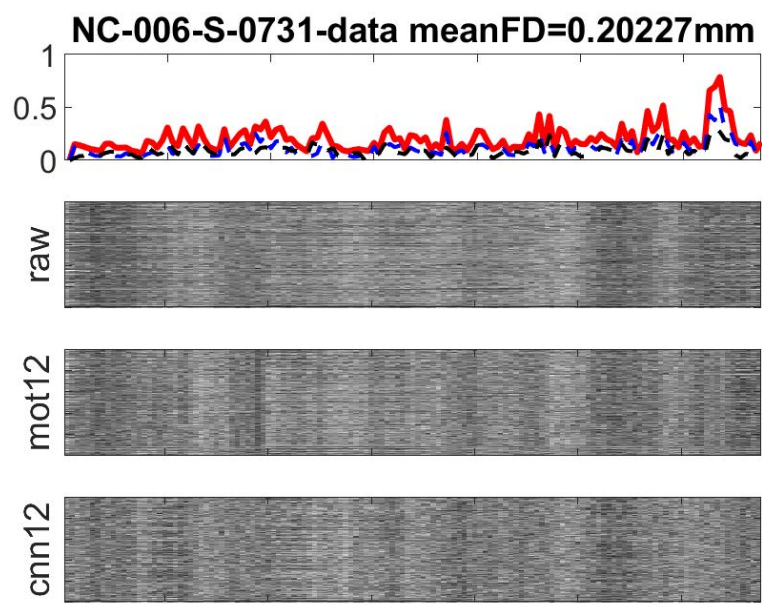

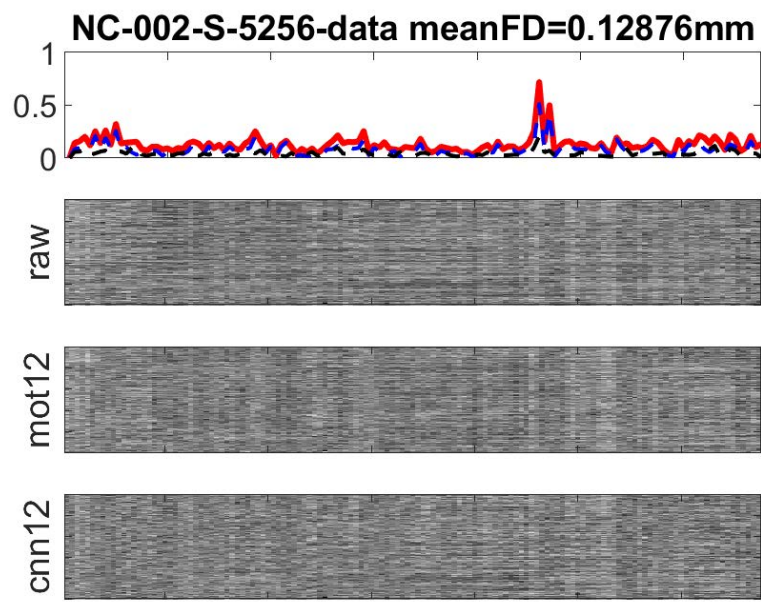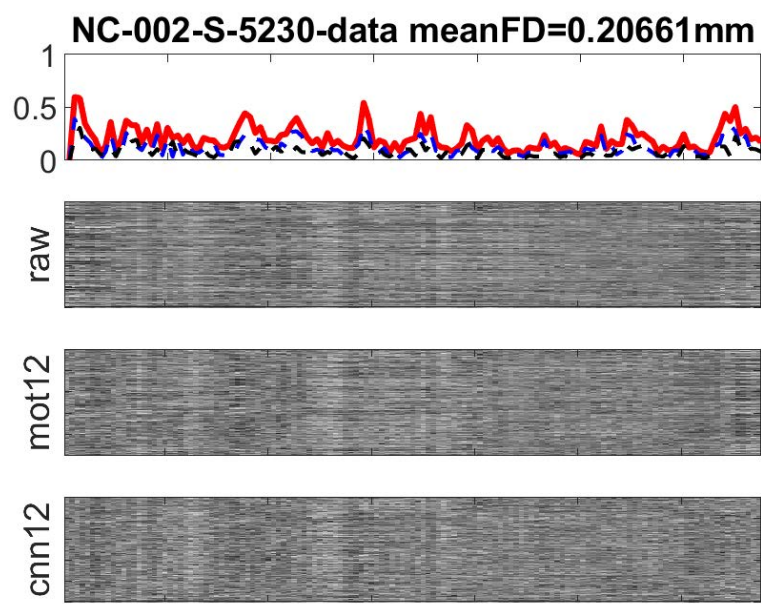

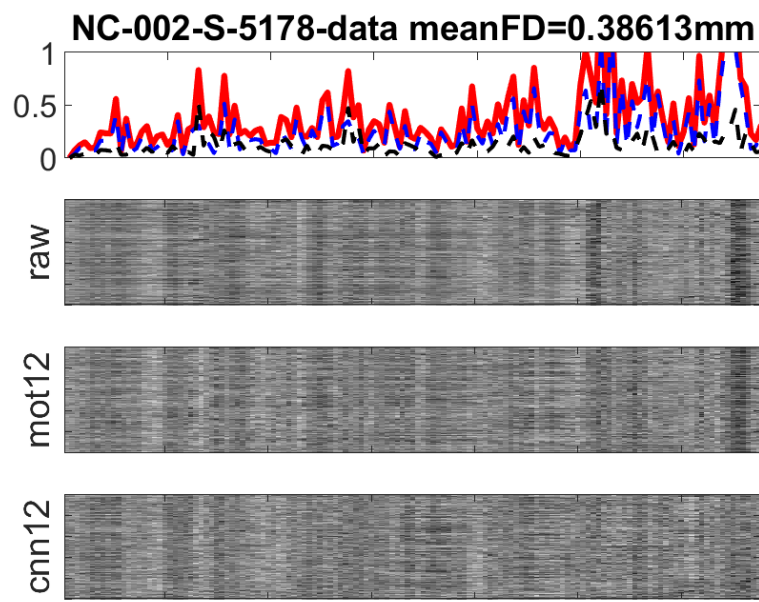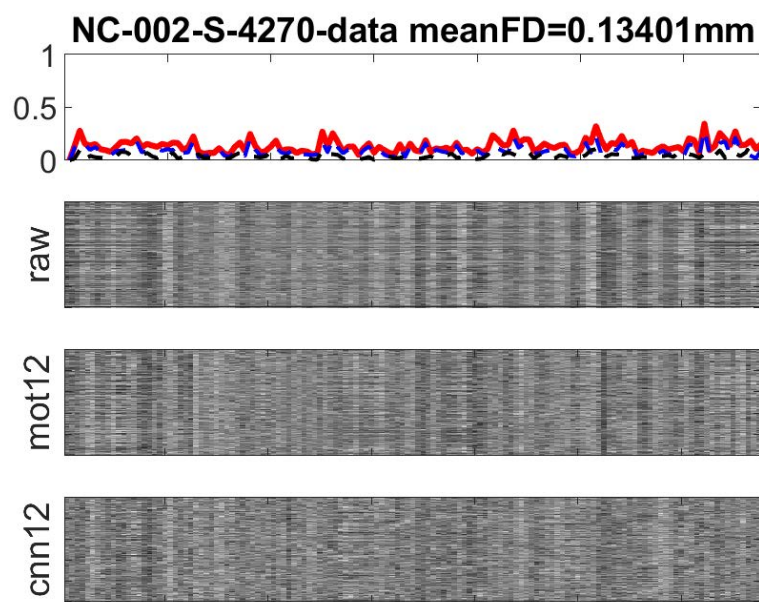

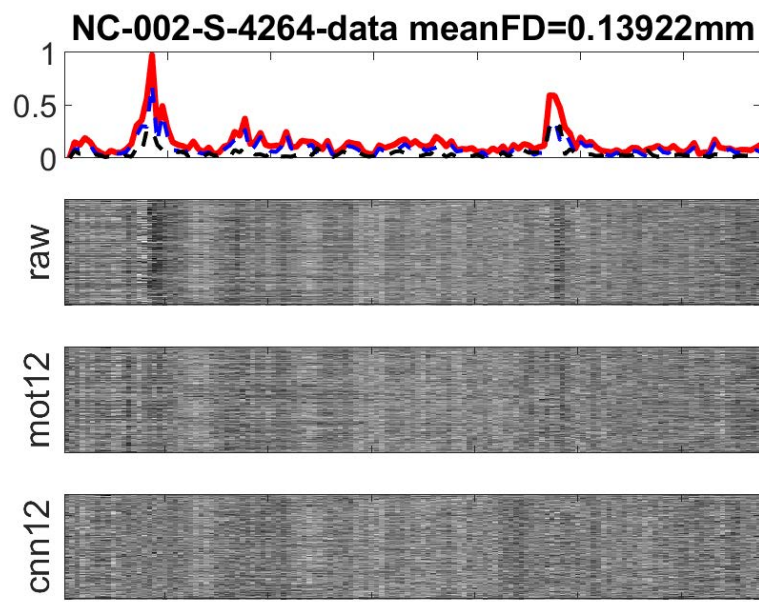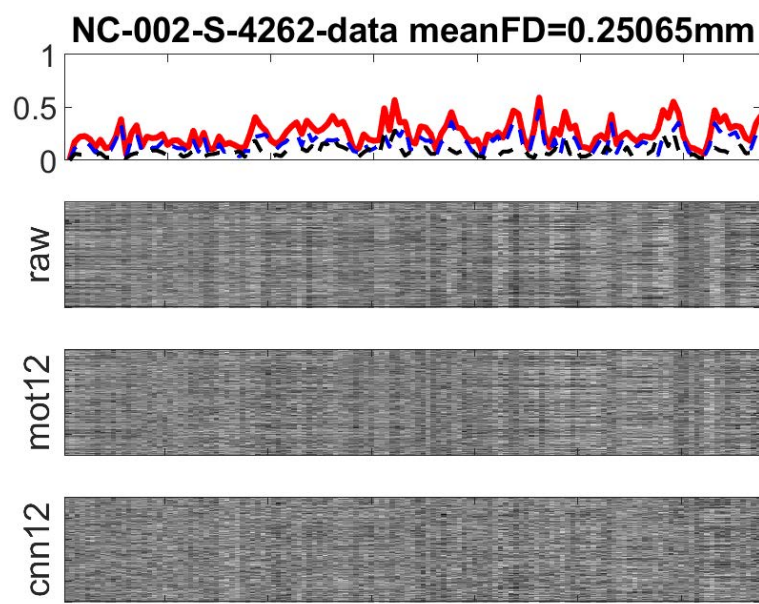

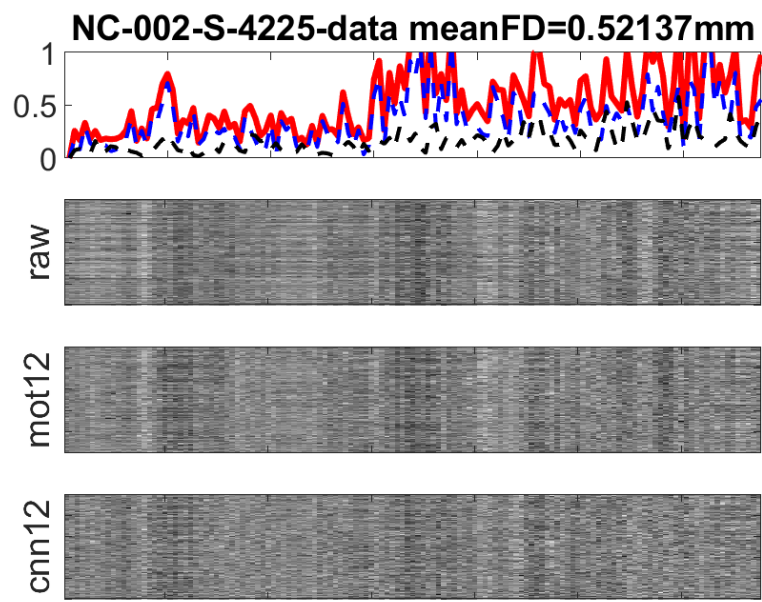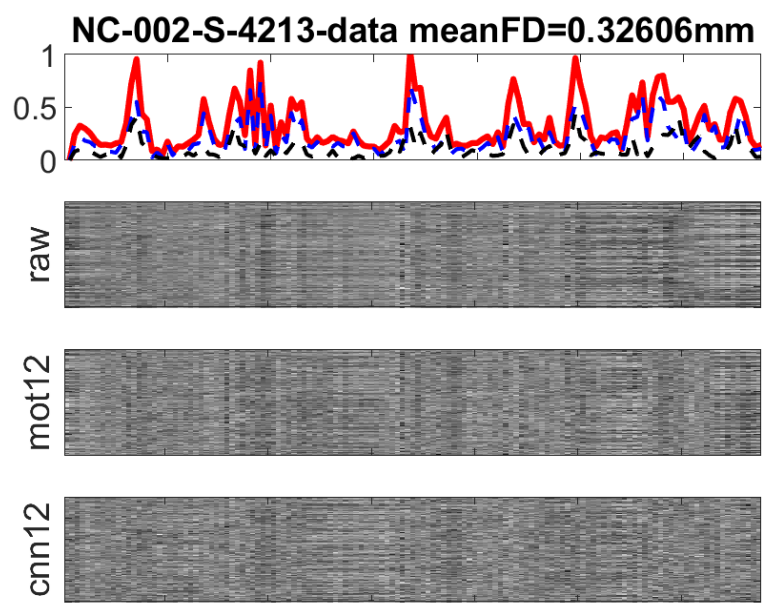

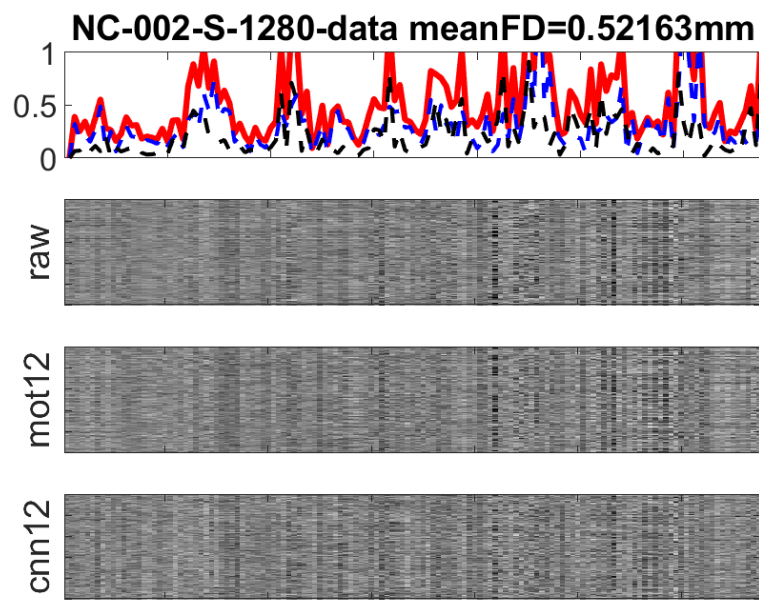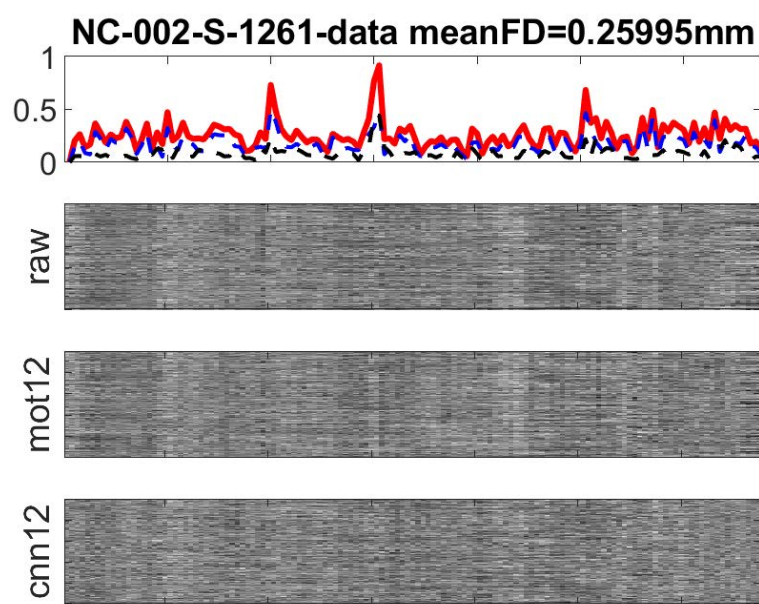

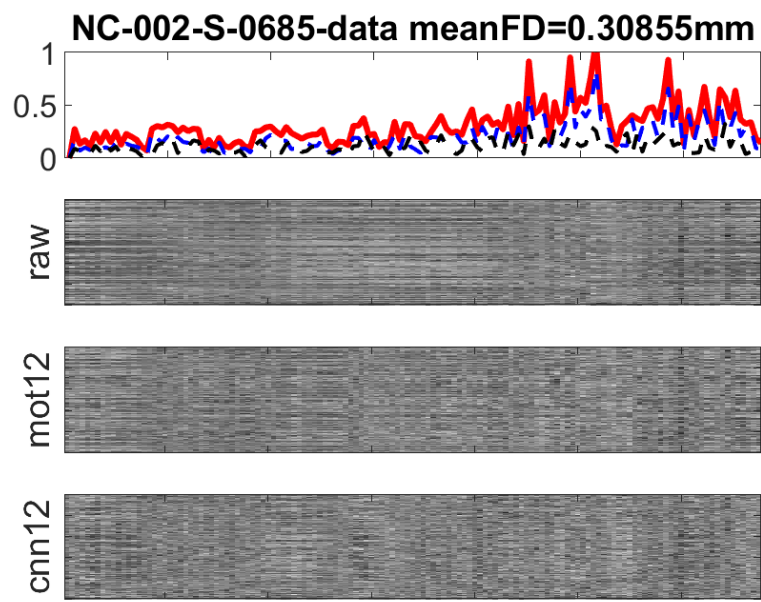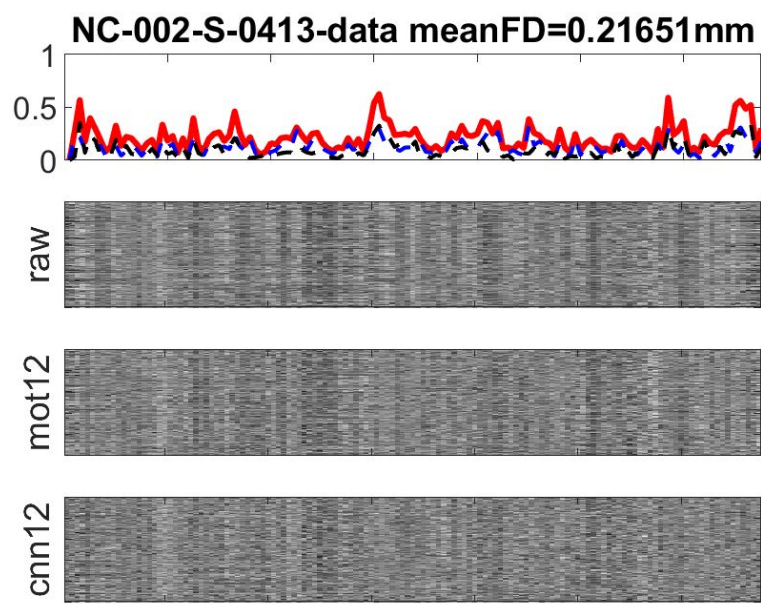

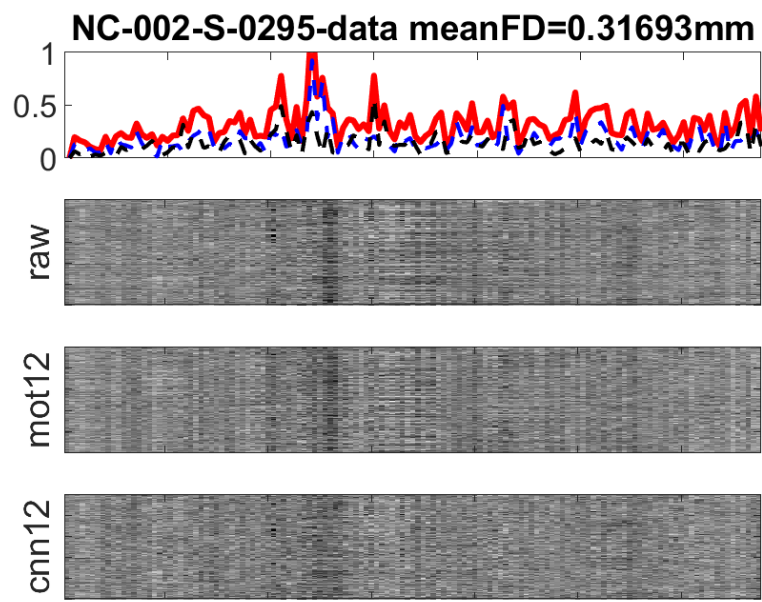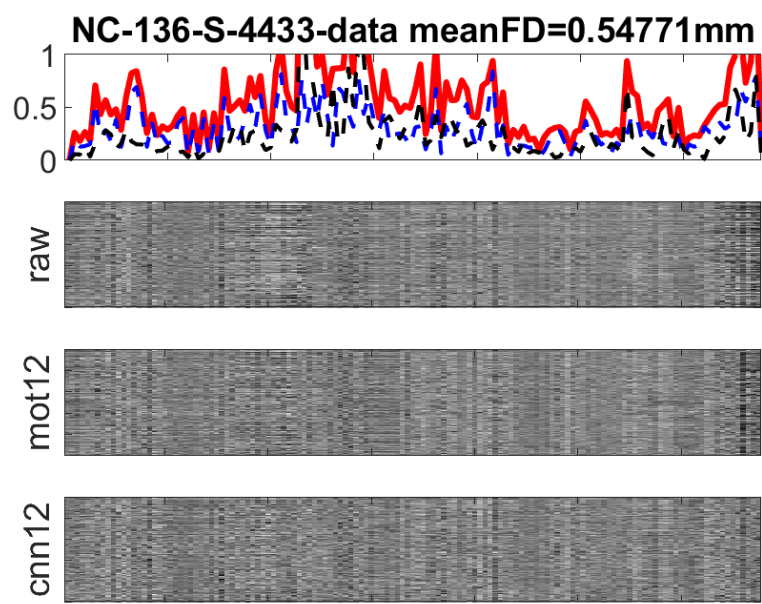

Supplement: Supplementary file 1 [file Data_Sheet_1.ZIP › supplementary/denoisedTS.pdf]
